# Comprehensive analysis of histophysiology, transcriptomics and metabolomics in goslings exposed to gossypol acetate: unraveling hepatotoxic mechanisms

**DOI:** 10.3389/fvets.2025.1527284

**Published:** 2025-01-21

**Authors:** Jun Yu, Haiming Yang, Jian Wang, Zixin Huang, Shi Chen, Hongchang Zhao, Jun Wang, Zhiyue Wang

**Affiliations:** ^1^Jiangsu Agri-Animal Husbandry Vocational College, Taizhou, China; ^2^College of Animal Science and Technology, Yangzhou University, Yangzhou, China

**Keywords:** hepatotoxic, gossypol acetate, goose, transcriptomics, metabolomics

## Abstract

Cottonseed meal is a promising alternative to soybean meal in poultry feed, but concerns over free gossypol limit its use. Although the general toxicity of free gossypol is well-known, its specific effects on the liver—the primary site where it accumulates—are less thoroughly studied, particularly at the molecular level. This study investigated the hepatotoxic effects of gossypol acetate (GA) on goslings through a comprehensive analysis combining morphology, transcriptomics, and metabolomics. Forty-eight 7-day-old male goslings with similar body weight (BW) were randomly assigned to two groups: a control group, receiving a saline solution (0.9%, 2.5 mL/kg BW), and a GA-treated group, administered GA at 50 mg/kg BW orally for 14 days. Histological analysis revealed signs of liver damage, including granular degeneration, hepatocyte enlargement, necrosis, and mitochondrial injury. Transcriptomic analysis identified 1,137 differentially expressed genes, with 702 upregulated and 435 downregulated. Key affected pathways included carbon metabolism, glycolysis/gluconeogenesis, pyruvate metabolism, propanoate metabolism, TCA cycle, fatty acid degradation, primary bile acid biosynthesis, tryptophan metabolism, cysteine and methionine metabolism, focal adhesion, and the PPAR signaling pathway. Metabolomic analysis revealed 109 differential metabolites, 82 upregulated and 27 downregulated, implicating disruptions in linoleic acid metabolism, arachidonic acid metabolism, cAMP signaling, and serotonergic synapse pathways. Overall, GA-induced hepatotoxicity involves impaired energy production, disrupted lipid metabolism, and abnormal liver focal adhesion, leading to liver cell dysfunction. These findings highlight the vulnerability of mitochondria and critical metabolic pathways, providing insights into the molecular mechanisms of GA toxicity and guiding future studies on mitigating GA-induced liver damage in goslings.

## 1 Introduction

The reduction and substitution of protein feeds, such as soybean meal, is a global concern driven by resource shortages and rising costs. Cottonseed meal, a by-product of the oil industry, has emerged as an attractive alternative protein source for poultry diets, including those of geese (1). However, free gossypol, the main antinutritional factor in cottonseed meal, poses significant risks to poultry growth and health. Gossypol is a polyphenolic compound that reduces protein digestibility by inhibiting pepsin and trypsin activity in the intestine and binding dietary iron (2). Upon entry into the poultry body, gossypol primarily accumulates in the liver, where it acts as a recognized hepatic toxin (3). Henry et al. (4) reported that feeding broiler chickens 800 and 1,600 mg/kg of gossypol caused perivascular lymphatic aggregation, biliary hyperplasia, and hepatic cholestasis. Similarly, a study on male meat ducks demonstrated that free gossypol in cottonseed meal, at levels ranging from 75 to 153 mg/kg of diet, induced cytoplasmic vacuolization of hepatocytes and bile duct epithelial cord hyperplasia, with increasing levels of free gossypol exacerbating liver damage (3). Our previous studies revealed that free gossypol levels above 56 mg/kg from cottonseed meal disrupts the redox balance in the liver of goslings, impairing the synthesis and metabolism of critical substances (5). Although the general toxicity of free gossypol is well-known, its specific effects on the liver—the primary organ for gossypol accumulation—remain less comprehensively understood, especially at the molecular level.

The liver is a vital organ containing numerous complex metabolites, including carbohydrates, amino acids, and lipids, which play essential roles in biosynthesis, metabolism, and detoxification. With the advancement of omics technology, these methods have become powerful tools for unveiling complex biological processes, especially in toxicological studies (6, 7). Transcriptomics allows comprehensive analysis of transcriptional sequences in specific tissues or organs through high-throughput sequencing (8), shedding light on the molecular mechanisms by which differential genes affect the host. Metabolomics, on the other hand, is used to detect changes in metabolites and their related pathways in response to various internal and external stimuli (9). The complex function of hepatic metabolites is well attuned to the advantages of the comprehensive and in-depth metabolomic analysis technology, which facilitates the analysis of changes in hepatic metabolites and provides a novel perspective for studying hepatic metabolism.

The hypothesis of this study was that exposure to gossypol acetate (GA), a form of free gossypol, induces toxic effects in the liver of goslings. Therefore, the study aimed to investigate the mechanisms of GA-induced hepatotoxicity in goslings through histomorphology, transcriptomics, and metabolomics.

## 2 Materials and methods

### 2.1 Birds and experimental design

The experimental design is described in detail in our previous published study (10). Briefly, 48 male goslings (Jiangnan White) at 7-day-olds with similar body weights (BW) were randomly distributed into two groups, each with 24 geese. The control group received an oral administration of saline solution (0.9%, 2.5 mL/kg BW). In contrast, the GA-treated (GA50) group received daily oral administrations of GA suspension at a dose of 50 mg/kg BW. Gossypol acetate (a form of free gossypol) was purchased from Shaanxi Bovlin Biotechnology Co, Ltd. (Xi'an, China) with a purity of ≥98.75%. The GA suspension was prepared by dissolving GA in 0.9% saline solution at 20 mg GA/mL. Every evening at 8:00 p.m., one person immobilized the goose while the other carefully instilled saline solution or GA suspension into the enlarged part of the esophagus using a round-tipped oral irrigating needle. The dosage was determined based on twice the daily intake of free gossypol established in our previous study on mildly intoxicated chicks (5), adjusted for the BW of 7-day-old chicks in the present study.

The diet was formulated primarily according to NRC (11) standards for geese and prior research from our laboratory (12, 13) (Supplementary Table S1). All birds were housed under identical environmental conditions, with a 24-h photoperiod and temperatures of 26–28°C from days 7 to 14 and an 18-h photoperiod of 24–26°C from days 15 to 21. The same formula feed and clean water were available *ad libitum* for all experimental geese. The experimental period lasted 14 days. Daily observations were conducted, and mortality and BW on death were recorded. Only one gosling in the GA50 group died during the entire trial period.

### 2.2 Sample collection

At 21 days of age, corresponding to the 14th days of GA administration, eight goslings with BW close to the average were selected from each treatment group for sampling. After neck bleeding and execution, four liver samples were collected from each bird. One sample was placed in pre-cooled 4% paraformaldehyde for light microscopy, another sample was placed in pre-cooled 2.5% glutaraldehyde (1–2 mm^3^) for transmission electron microscopy, the third sample was stored at −20°C for analysis of gossypol residues, and the last sample was quickly snap-frozen in liquid nitrogen and stored at −70°C for transcriptome and metabolome analysis.

### 2.3 Gossypol residue in liver

The total content of gossypol in liver tissue was assessed using high-performance liquid chromatography (HPLC), according to the methodology of our previous study (1).

### 2.4 Histomorphological analysis

Fixed liver tissue samples were dehydrated, embedded in paraffin wax, sectioned (5 μm thick), and stained with hematoxylin and eosin. The liver sections were scanned using a panoramic section scanner (Pannoramic DESK/MIDI/250/1000, 3 Dhistech, Budapest, Hungary) and visualized using the corresponding scan browsing software.

### 2.5 Transmission electron microscopy analysis

Liver tissues were fixed in 2.5% glutaraldehyde at 4°C for 4 h, then washed 3 times with 0.1 mol/L phosphate buffer at 4°C. The samples were further fixed in 1% osmium tetroxide at room temperature (20°C), dehydrated in a gradient of 70%−100% acetone, and embedded in EPON812 epoxy resin. Embedded samples were localized under a light microscope and then sectioned (60 nm thick) using an ultrathin microtome (Leica UC7, Leica Biosystems, Wetzlar, Germany). The sections were stained with uranyl acetate and lead citrate and visualized using a transmission electron microscope (HT7700, HITACHI, Tokyo, Japan).

### 2.6 Preparation of cDNA libraries and illumina sequencing for transcriptome analysis

Total RNA was extracted from the liver using the TRIzol reagent (Tiangen Biochemical Technology Co., Ltd., Beijing, China), following the manufacturer's instructions. The RNA integrity and purity were then assessed using an Agilent Bioanalyzer 2100 (Agilent Technologies, Santa Clara, CA, USA).

Subsequently, samples for transcriptome analysis were prepared using the NEBNext^®^ Ultra™ RNA Library Prep Kit for Illumina^®^ (San Diego, CA, USA). The mRNA was purified from total RNA using poly-T oligo-attached magnetic beads and then randomly fragmented in a Fragmentation Buffer. The first strand of cDNA was synthesized using a random hexamer primer and M-MuLV Reverse Transcriptase. The second strand of cDNA synthesis was subsequently performed using RNase H, buffer, dNTPs, and DNA Polymerase I. The purified double-stranded cDNA was end-repaired, A-tailed, and ligated to the sequencing junction. The 250–300 bp cDNA fragments were selected using AMPure XP beads (Beckman Coulter, Beverly, CA, USA). PCR amplification was performed, and the PCR product was purified by AMPure XP beads again to obtain the final library. After library construction, preliminary quantification was performed using a Qubit 2.0 Fluorometer. The libraries were diluted to 1.5 ng/μL, and the insert size was examined using an Agilent 2100 bioanalyzer system (Agilent Technologies, Santa Clara, CA, USA).

The cDNA library was subsequently sequenced on the Illumina sequencing platform (Illumina NovaSeq 6000, Illumina, San Diego, CA, USA), generating 150-bp paired-end reads. Raw reads were generated from the sequencing data, and the raw data (in fastq format) were processed using fastp software. In this step, clean data (clean reads) were obtained by removing reads containing adapter, ploy-N, and low-quality reads from raw data. Additionally, Q30 and GC content of the clean data were calculated to assess quality. All downstream analyses were based on these high-quality clean reads. Clean Reads were aligned to the Sichuan White goose reference genome using HISAT2 software (version 2.2.1), and the number of reads mapped to each gene was calculated using featureCounts (version 1.5.0-p3). Each gene's fragments per kilobase million (FPKM) value was computed based on the exon length. Gene expression levels were normalized using the DESeq2 package (version 1.20.0) in the R environment, and differentially expressed genes (DEGs) were identified with the |log_2_ fold change| > 0.485 and *P*-value < 0.05.

All DGEs were classified and annotated using Gene Ontology (GO) term enrichment analysis and Kytoto Encyclopedia of Genes and Genomes (KEGG) pathway enrichment analysis by the clusterProfiler R package (version 3.8.1). The analysis applied a significance threshold of adjusted *P*-value (padj) < 0.05. The enrichment analysis was based on the principle of hypergeometric distribution, and the results were enriched for all differential gene sets, upregulated differential gene sets, and downregulated differential gene sets for each differential comparison combination.

### 2.7 Quantitative reverse transcription PCR (qRT-PCR) validation

To verify the accuracy of the transcriptomic data, 6 DEGs were randomly selected for validation using qRT-PCR. The qRT-PCR was performed using a CFX96™ Real-Time System (BIO-RAD, Singapore) with Hieff qPCR SYBR Green Master Mix (Yeasen, Shanghai, China) according to the manufacturer's protocol. The relative mRNA expression levels were calculated using the 2^−Δ*ΔCt*^ method with β-actin as the housekeeping gene. The sequences of the specific primers used are listed in Supplementary Table S2, and melt curves and melt peaks of the primers are shown in Supplementary Figure S1.

### 2.8 Metabolite extraction, derivatization, and untargeted metabolomic analysis

Metabolite extraction was conducted following the method described by Warren et al. (14) with minor modifications. Liver samples (50 mg) were homogenized in 1,000 μL tissue extract composed of 75% 9:1 methanol (Sigma-Aldrich): chloroform (Sigma-Aldrich), and 25% H_2_O. The mixture was subjected to ultrasonication for 30 min at 20°C. After placing on ice for 30 min, the samples were centrifuged at 12,000 rpm at 4°C for 10 min. The supernatant was collected and concentrated to dryness in a vacuum concentrator. Then, 200 μL of 50% acetonitrile solution, containing 2-chloro-L-phenylalanine solution (4 ppm), was added to the dried metabolites to re-dissolve the samples, which were then filtered through a 0.22 μm filter membrane and used for LC-MS analysis.

The analyses were performed using a Vanquish UHPLC system (Thermo Fisher Scientific, Waltham, MA, USA) coupled with a Q Exactive Focus mass spectrometer (Thermo Fisher Scientific, Waltham, MA, USA) equipped with an electrospray ionization (ESI) source. Chromatographic separation was performed on an ACQUITY UPLC HSS T3 column (150 × 2.1 mm, 1.8 μm, Waters, Milford, MA, USA). A 2 μL sample was loaded onto the column, maintained at 40°C with a 0.25 mL/min flow rate. For LC-ESI (-)-MS analysis, the analytes were separated using (A) acetonitrile and (B) ammonium formate (5 mM) under the following gradient conditions: 0–1 min, 2% A; 1–9 min, 2%−50% A; 9–12 min, 50%−98% A; 12–13.5 min, 98% A; 13.5–14 min, 98%−2% A; and 14–17 min, 2% A. For LC-ESI (+)-MS analysis, the mobile phases consisted of (C) 0.1% formic acid in acetonitrile (v/v) and (D) 0.1% formic acid in water (v/v), with the following gradient: 0–1 min, 2% C; 1–9 min, 2%−50% C; 9–12 min, 50%−98% C; 12–13.5 min, 98% C; 13.5–14 min, 98%−2% C; and 14–20 min, 2% C. Mass spectrometry settings were as follows: spray voltage, 3.50 kV and −2.50 kV for ESI(+) and ESI(−), respectively; sheath gas pressure, 30 arb; aux gas flow, 10 arb; capillary temperature, 325°C; MS1 range, m/z 81–1000; MS1 resolving power, 70000 FWHM; number of data dependant scans per cycle, 3; MS/MS resolving power, 17500 FWHM; normalized collision energy, 30%; dynamic exclusion time, automatic.

The raw LC-MS data were converted to the mzXML format using ProteoWizard software (version 3.0.8789) and processed with the XCMS package (version 3.8.1) for feature detection, peak alignment, and retention-time correction. Metabolites were identified based on accuracy mass (< 30 ppm) and MS/MS data, which were matched against several databases, including HMDB, Massbank, LipidMaps, mzCloud, and KEGG. A robust LOESS signal correction was applied to correct any systematic bias for data normalization, as Gagnebin et al. (15) described. After normalization, only ion peaks with relative standard deviations of < 30% in quality control (QC) samples were retained to ensure reliable metabolite identification. Next, the data underwent multivariate statistical analysis, including principal component analysis (PCA), partial least squares discriminant analysis (PLS-DA), and orthogonal partial least-square discriminant analysis (OPLS-DA) using R ropls package (version 1.22.0). The variable importance on the projection (VIP) value obtained from OPLA-DA was used to screen for potential distinguishing metabolites. Univariate *t*-tests were conducted to compute statistical significance, with differentially abundant metabolites defined by the criteria: VIP > 1, *P*-value < 0.05. Metabolites of interest were further visualized using volcano plots based on log_2_ fold change (FC) and -log_10_ (*P*-value). Additionally, the KEGG database was used to evaluate the functions of these metabolites and their associated pathways using MetaboAnalyst 5.0. The analysis applied a significance threshold of padj < 0.05.

### 2.9 Statistical analysis

Every bird was treated as one experimental unit. Statistical assessments were conducted using the Student's *t*-tests, employing SPSS (version 21.0). Results were considered statistically significant at the *P* < 0.05 level. Data are displayed as mean ± SE.

## 3 Results

### 3.1 Hepatic gossypol residues

Total gossypol was not detected in the livers of the control group, whereas the total gossypol content in the livers of the GA50 group was 4.48 ± 0.14 mg/kg dry matter.

### 3.2 Hepatic histology

**Figure 1:**
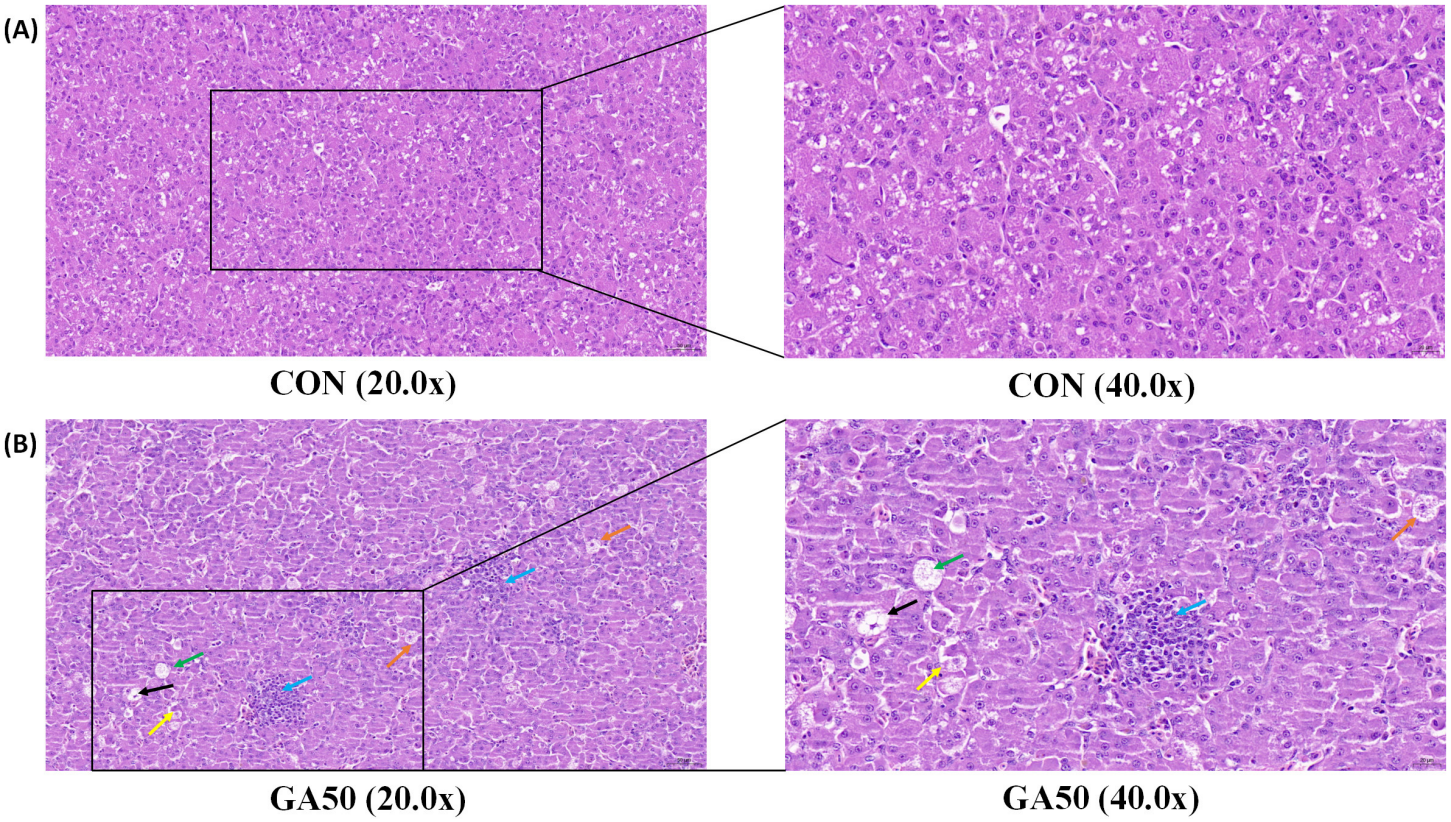
Hepatic histology of goslings from the control and gossypol acetate (GA) groups. The control **(A)** and the GA50 **(B)** groups were orally administered 0 and 50 mg/kg BW GA daily, respectively. Orange arrows indicate enlarged hepatocytes, yellow arrows indicate pale stained nuclei, green arrows indicate microscopic reddish granules in the cytoplasm, black arrows indicate the disappearance of the cytoplasm of hepatocytes, and blue arrows indicate lymphocytic infiltration.

As shown in Figure 1, the liver of goslings was characterized mainly by degeneration of hepatocyte granules, enlarged hepatocytes, pale-stained nuclei, microscopic reddish particles in the cytoplasm, and occasional disappearance of the cytoplasm of hepatocytes; a small number of hepatocytes were focally necrotic, with fragmented nuclei and disintegrated cytoplasm, accompanied by infiltration of lymphocytes.

### 3.3 Hepatic ultrastructure

**Figure 2:**
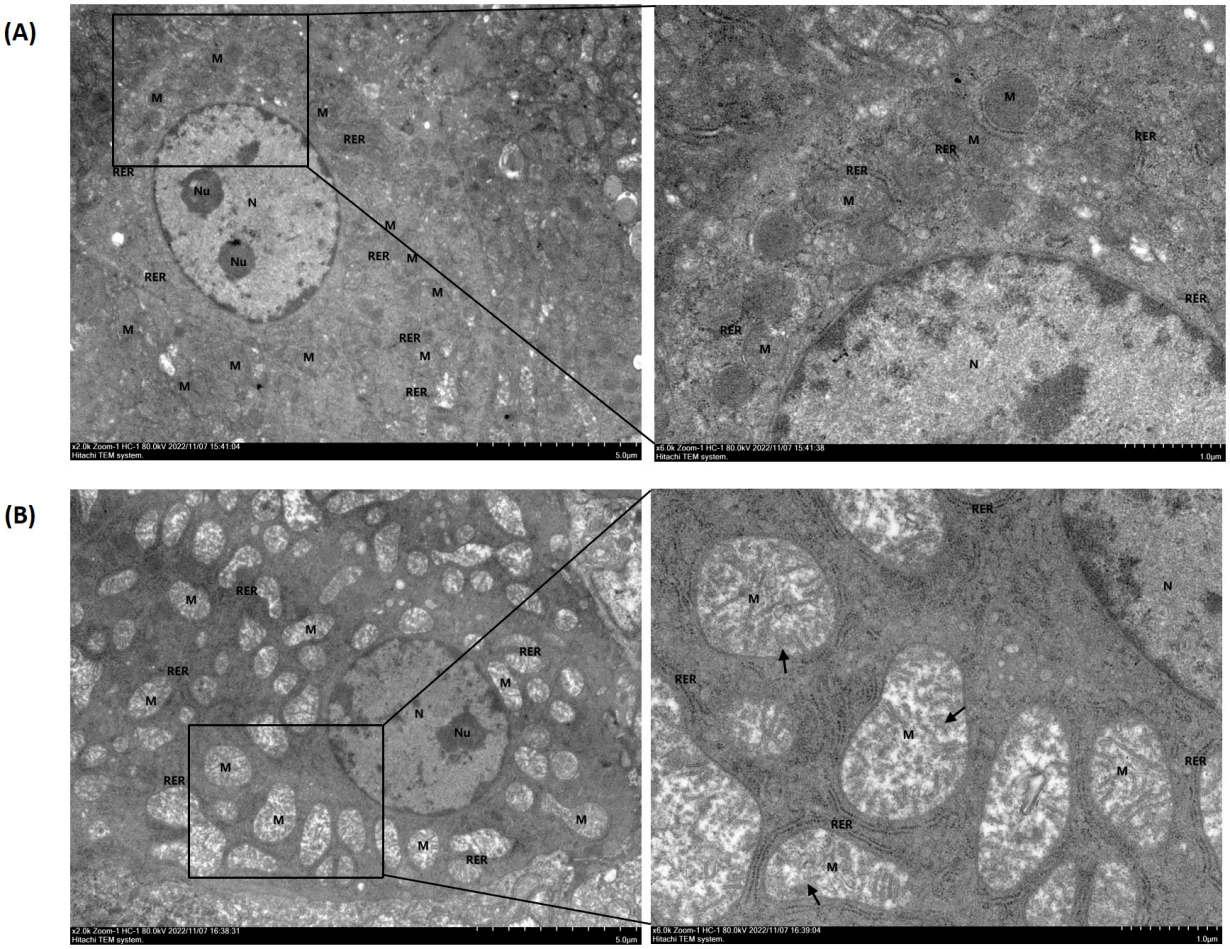
Hepatic ultrastructure of goslings in the control and gossypol acetate (GA) groups. The control **(A)** and the GA50 **(B)** groups were orally administered 0 and 50 mg/kg BW GA daily, respectively. The nucleus, nucleolus, mitochondria, and rough endoplasmic reticulum were labeled N, Nu, M, and RER, respectively. Black arrows indicate cristae that are broken, shortened, reduced, or flocculated.

As can be seen in Figure 2, after 14 days of administration of GA, mitochondria in the liver showed signs of damage, including moderate swelling, enlarged volume, matrix dissolution, and shallowness. The cristae were primarily broken, shortened, reduced, or flocculent; in some cases, individual membranes were damaged.

### 3.4 Liver transcriptome analysis

To investigate the changes in gene expression in the liver of goslings exposed to GA, the RNA-seq technique was utilized to compare the differences between the GA50 and the control group. After sequencing the sample cDNA, raw reads per sample ranged from 39,542,614 to 46,163,350. After filtering, the number of clean reads ranged from 38,037,932 to 44,854,284 per sample, with Q30 base percentages above 87%, meeting the criteria for subsequent data analysis (Supplementary Table S3). When comparing the filtered sequences with the reference genome of Sichuan White geese, the alignment rate of the eight samples ranged from 74.2% to 81.06%, and the unique alignment rate ranged from 72.02% to 78.65% (Supplementary Table S3). The comparison results indicated that the sequencing quality was high, and the data met the requirements for further analysis.

**Figure 3:**
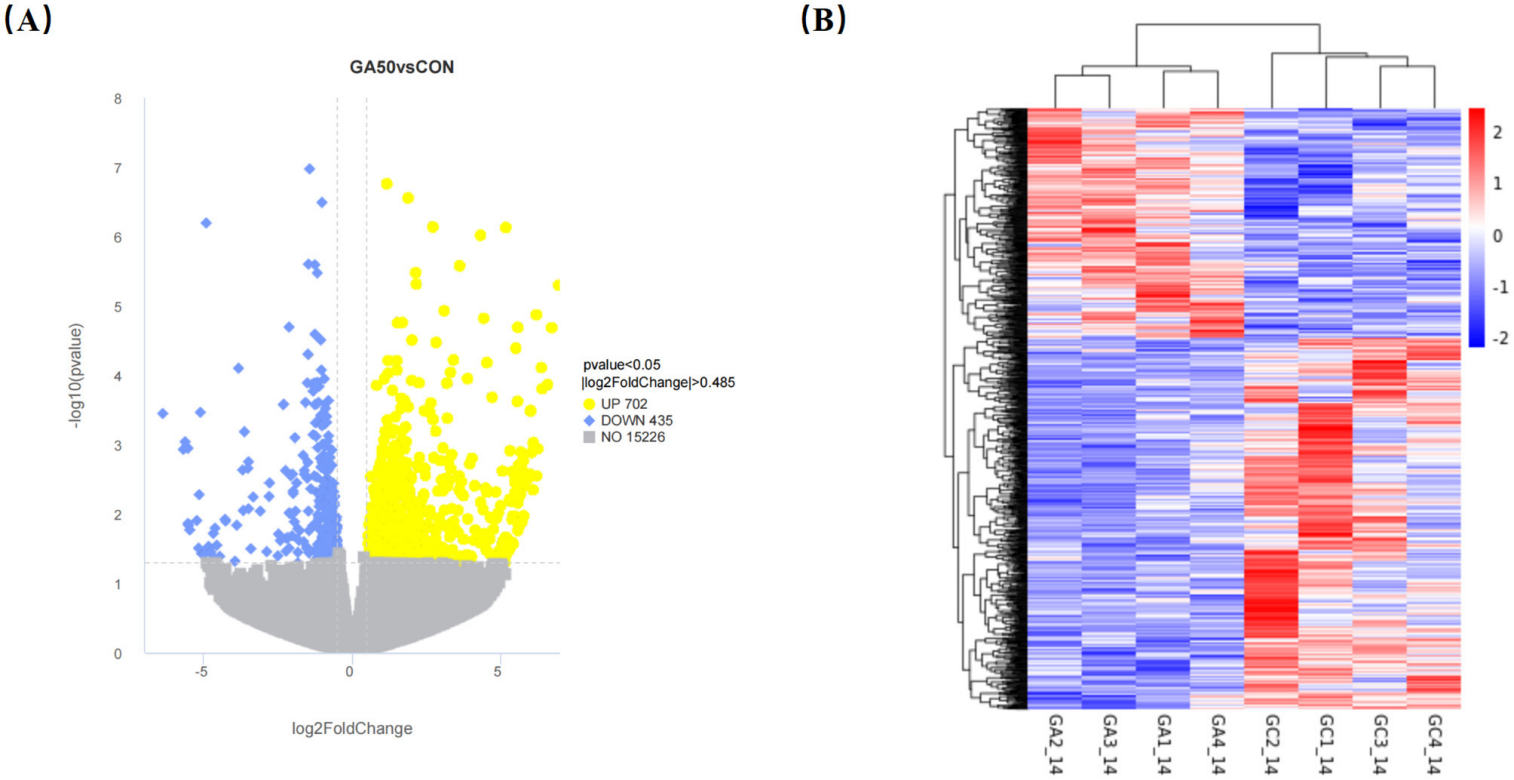
Volcano map **(A)** and cluster analysis **(B)** of differentially expressed genes in the liver of goslings. In the volcanic map, the *x*-axis is log_2_ FoldChange, and the *y*-axis is -log_10_ (*P*-value). The yellow dots represent the upregulated genes, the blue dots represent the downregulated genes, and the gray dots represent the non-differentially expressed genes. The dotted lines represent the threshold line of the differential gene screening standard. GA1_14, GA2_14, GA3_14, and GA4_14 belong to control group, and GC1_14, GC2_14, GC3_14, and GC4_14 belong to GA50 group.

**Figure 4:**
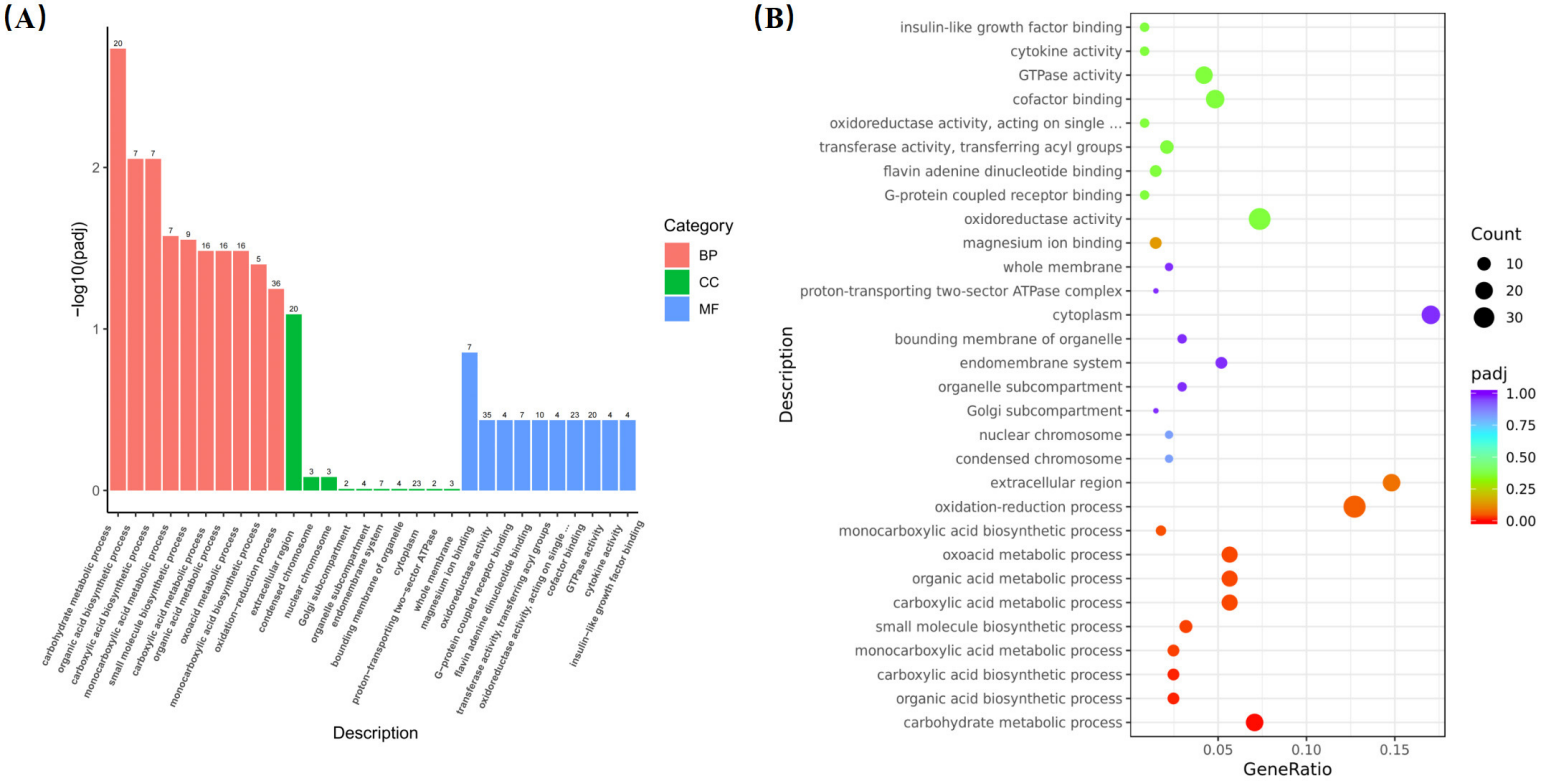
Go analysis of differentially expressed genes. **(A)** Annotation classification. **(B)** Scatter diagram of go enrichment analysis.

Transcriptome analysis revealed that 1,137 DEGs were identified in the liver between the GA50 and control group, with 702 genes upregulated and 435 genes downregulated (*P* < 0.05; Supplementary Table S4). DEGs from the different samples were further subjected to cluster analysis, and the results are displayed in Figure 3. To gain insight into the functional distribution of the DEGs, GO analysis was performed. GO is an international standardized gene functional classification system with three categories: molecular functions, cellular components, and biological processes. As shown in Figure 4, functional analysis of these DEGs through GO revealed that the most significant GO terms were primarily associated with biological processes (padj < 0.05). These processes included carbohydrate metabolism (GO:0005975), organic acid biosynthetic process (GO:0016053), carboxylic acid biosynthetic process (GO:0046394), monocarboxylic acid metabolic process (GO:0032787), small molecule biosynthesis process (GO:0044283), carboxylic acid metabolic process (GO:0019752), organic acid metabolic process (GO:0006082), oxoacid metabolic process (GO:0043436), and monocarboxylic acid biosynthetic process (GO:0072330).

Furthermore, the KEGG pathway enrichment analysis indicated that the DEGs involved multiple metabolic pathways. As illustrated in Table 1, these pathways, including carbon metabolism, glycolysis/gluconeogenesis, pyruvate metabolism, propanoate metabolism, TCA cycle, fatty acid degradation, primary bile acid biosynthesis, tryptophan metabolism, cysteine and methionine metabolism, focal adhesion, and the PPAR signaling pathway, were significantly affected (padj < 0.05).

**Table 1:** KEGG pathway enrichment analysis of differentially expressed genes (DEGs).

**Pathway database**	**Pathway**	**DEGs**	**geneName**		***P*-value**	***Q*-value**
			**Up-regulated**	**Down-regulated**		
Global and overview maps	Carbon metabolism	21	*ALDOC; HK3*	*ALDOB; EHHADH; DLAT; PDHB; PDHA1; MDH1; PSPH; SUCLG1; AGXT; PRPS2; ECHS1; TALDO1; SHMT2; MMUT; PFKL; GPI; SDHA; DLD; LOC106032592*	0.00014	0.00665
Carbohydrate metabolism	Glycolysis/gluconeogenesis	15	*ALDOC; HK3*	*LDHB; ALDOB; DLAT; PDHB; PDHA1; ALDH3A2; PFKL; G6PC1; LOC125181711; GPI; DLD; ALDH7A1; GALM*	0.00003	0.00344
	Pyruvate metabolism	11	*ACACB*	*LDHB; DLAT; PDHB; PDHA1; MDH1; ALDH3A2; GLO1; LOC125181711; DLD; ALDH7A1*	0.00005	0.00344
	Propanoate metabolism	9	*ACACB; ABAT*	*ACSS3; LDHB; EHHADH; SUCLG1; ECHS1; MMUT; DLD*	0.00097	0.02323
	Tricarboxylic acid (TCA) cycle	8		*DLAT; PDHB; PDHA1; MDH1; SUCLG1; LOC125181711; SDHA; DLD*	0.00166	0.03408
Lipid metabolism	Fatty acid degradation	10	*ACSL4*	*CPT2; EHHADH; ACADL; ACAA2; ECHS1; ACADM; ALDH3A2; ECI2; ALDH7A1*	0.00044	0.01272
	Primary bile acid iosynthesis	5	*LOC106039033; LOC106041408*	*AMACR; HSD17B4; SCP2*	0.00305	0.03997
Amino acid metabolism	Tryptophan metabolism	12		*EHHADH; KYNU; HAAO; LOC125182130; ECHS1; ALDH3A2; TDO2; KYAT3; ALDH8A1; DLD; ALDH7A1; AOX1*	0.00025	0.00883
	Cysteine and methionine metabolism	10	*DNMT3B*	*MAT2A; LDHB; MDH1; GCLC; CDO1; GCLM; LOC106042084; MTR; KYAT3*	0.00300	0.03997
Cellular community-eukaryotes	Focal adhesion	28	*SPP1*; *PDGFA*; *TLN1*; *RAPGEF1*; *COL6A1*; *VAV1*; *LAMC1*; *ERBB2*; *THBS4*; *PXN*; *RAC2*; *COL1A2*; *MYLK*; *VAV2*; *LAMA3*; *SRC*; *JUN*; *LOC125181877*; *PDGFD*; *COL6A2*; *CCND3*; *JUND*; *COL1A1*; *LOC125181834*; *ARHGAP35*	*AKT1*; *EGF*; *PMVK*	0.00229	0.03665
Endocrine system	PPAR signaling pathway	13	*LOC106039033*; *LOC106033870*; *ACSL4*; *SLC27A1*	*CPT2*; *EHHADH*; *ACADL*; *GK*; *ACADM*; *FABP5*; *SCP2*; *LOC125181711*; *LOC106040417*	0.00223	0.03665

To validate the accuracy of the transcriptome sequencing results, RT-qPCR was conducted on six randomly selected DEGs from the livers of GA50 and control goslings. Although the magnitude of expression differences varied between methods, the RT-qPCR results (*P* < 0.05) consistently showed similar trends in gene upregulation or downregulation compared to the transcriptome sequencing, confirming the reliability of the data (Supplementary Figure S2).

### 3.5 Liver untargeted metabolomic analysis

**Figure 5:**
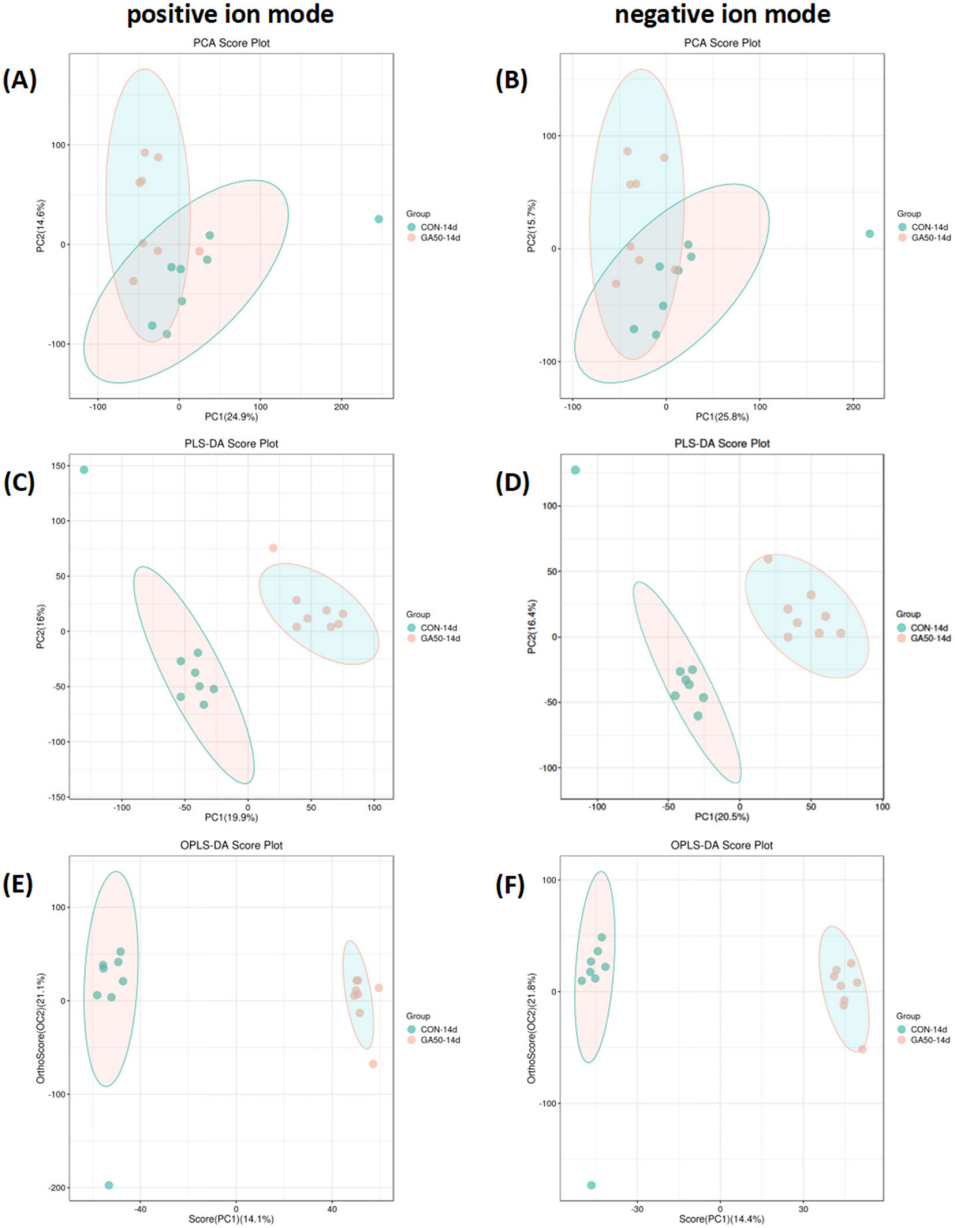
PCA **(A, B)**, PLS-DA **(C, D)**, and OPLS-DA **(E, F)** score plots in the liver of goslings. The abscissa represents the first principal component, PC1, and the ordinate represents the second principal component PC2; each point in the figure represents a sample, and colors represent different groups.

To assess the impact of GA on metabolite levels in goslings, an untargeted liver metabolomics analysis was conducted using HPLC-MS. The OPLS-DA score plot demonstrated a distinct separation between the GA50 and control groups, indicating significant differences in their metabolic profiles (Figure 5).

**Figure 6:**
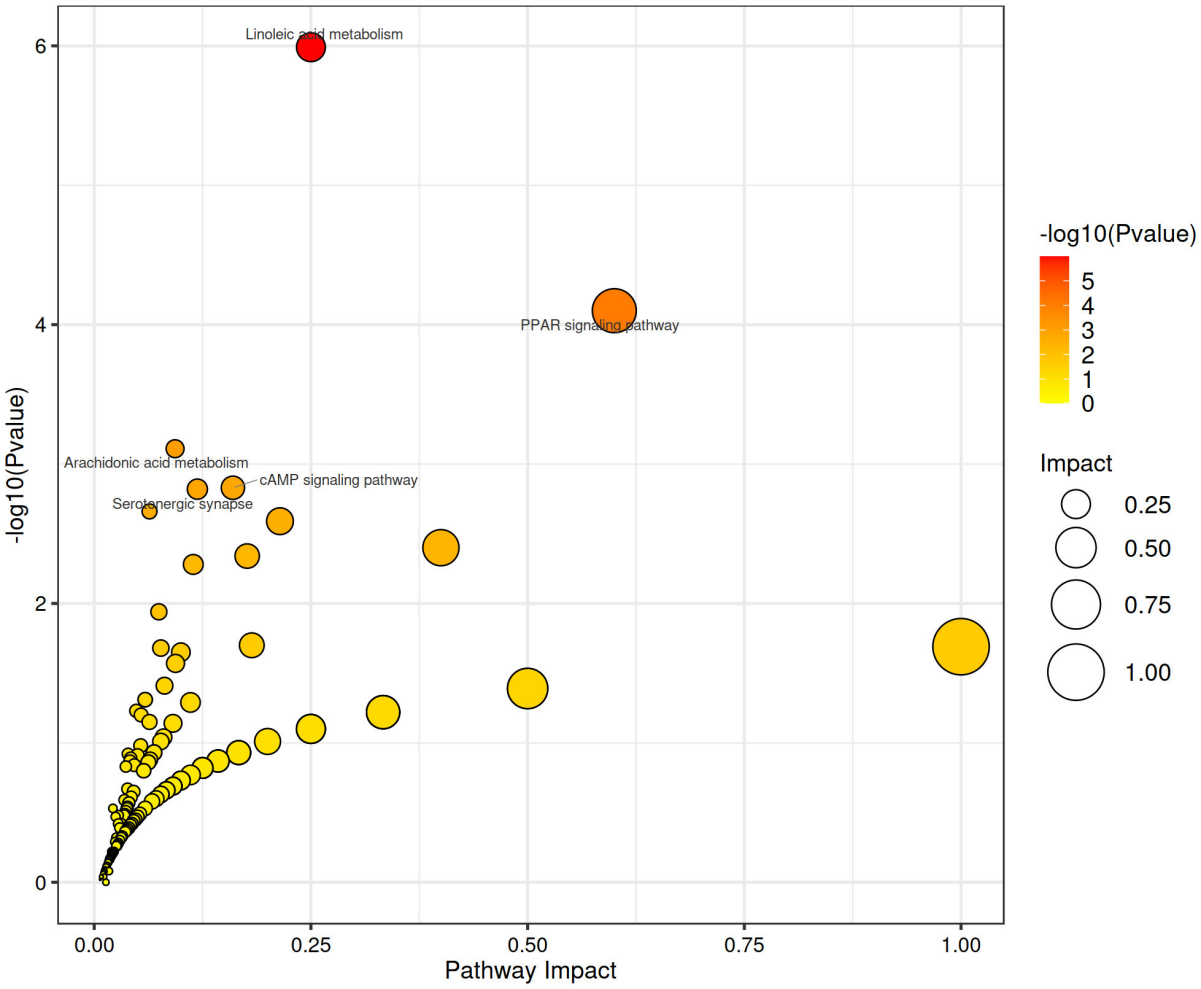
Functional enrichment analysis using KEGG. The *x*-axis represents the ratio of differential genes annotated to a specific KEGG pathway to the total number of differential genes, and the *y*-axis represents the corresponding KEGG pathways.

A total of 566 identifiable metabolic compounds were detected, covering a broad range of metabolites, including sugars, proteins, lipids, and their degradation products. Using specific screening criteria, 126 differential metabolites were identified between the GA50 and control groups, with 80 metabolites significantly upregulated and 46 significantly downregulated (Supplementary Table S5). KEGG pathway analysis revealed that GA exposure profoundly affected several metabolic pathways, including linoleic acid metabolism, PPAR signaling pathway, arachidonic acid metabolism, cAMP signaling pathway, and serotonergic synapse (padj < 0.05; Figure 6). These pathways are crucial for various metabolic and physiological processes, suggesting that GA induces broad metabolic disruptions in the liver.

## 4 Discussion

In poultry, gossypol predominantly accumulates in the liver, rendering it a potent hepatotoxin. Recent studies in meat ducks and geese have shown that free gossypol in cottonseed meal causes liver damage and metabolic disturbances, with a dose-dependent effect (3, 5). Similarly, El-Sharaky et al. (16) found that intraperitoneal injection of GA (5, 10, and 20 mg/kg BW) in male rats caused severe hepatocellular disintegration, necrosis, abnormal localization of nuclei, hepatic vein dilation, and congestion, along with vacuolization of fat cells. In contrast, He et al. (17) observed no histological damage in the liver, kidney, or oviduct tissues of laying hens fed free gossypol (28.35 mg/kg) or low-gossypol cottonseed meal. The hepatocytes in these hens displayed a normal histological appearance without fatty degeneration. These findings highlight that gossypol-induced liver damage varies based on factors such as species, gossypol dosage, and mode of administration. In the present study, oral administration of GA resulted in hepatocyte damage in goslings, primarily affecting the mitochondria. The damaged mitochondria appeared swollen and enlarged, with solubilized and shallower matrix, broken, shortened cristae, and occasional ruptured membranes. Since mitochondria are essential for energy production, their impairment can compromise hepatocyte energy metabolism, leading to widespread metabolic disruptions, particularly in protein and lipid metabolism. To further explore the mechanisms underlying GA-induced hepatotoxicity, transcriptomic and metabolomic analyses were conducted. These analyses provided insights into the molecular and metabolic pathways altered by GA exposure in goslings.

The response of animals to the toxic effects of gossypol is a complex physiological process that cannot be fully explained by the regulation of a single gene. Therefore, this comprehensive study employed RNA-seq technology to thoroughly investigate DEGs at the transcriptional level in the liver, aiming to elucidate the molecular mechanisms underlying gossypol's toxic effects. In this study, the effects of GA include global and overview maps, carbohydrate metabolism, lipid metabolism, amino acid metabolism, cellular community—eukaryotes and endocrine system. These findings suggest that GA administration disrupts hepatic basal substance metabolism, cellular interactions, and endocrine system functions in goslings.

Among the enriched pathways, the focal adhesion pathway has emerged as an increasingly important tool for evaluating and studying plant toxicity (18). Focal adhesions are specialized structures formed at the contact points between cells and the extracellular matrix. They serve multiple roles: some components of focal adhesions link membrane receptors to the actin cytoskeleton, thus coordinating cell structure and movement; other components function as signaling molecules, including protein kinases, phosphatases, enzyme substrates, and various connexins (19–21). The focal adhesion pathway is critical in regulating various cellular processes, such as cell migration, proliferation, differentiation, gene expression, and apoptosis (22). The ECM-receptor interaction pathway, a crucial upstream regulator of focal adhesion, plays a vital role in maintaining cellular structure and facilitating communication between the extracellular matrix (ECM) and cells. ECM communicates with cells primarily through the focal adhesion pathway (23). In this interaction, integrins serve as the principal transmembrane proteins, forming α,β heterodimers that bridge the ECM and the intracellular environment. These integrins possess large extracellular domains that bind various ECM proteins, while their short cytoplasmic tails interact with cytoskeletal signaling networks, ensuring coordinated cellular responses (21). Intracellularly, integrins are associated with key proteins like talin, filamin, and actinin, which form adhesion complexes. Talin is essential to the early assembly of adhesion complexes (24), as evidenced by its structure, which associates integrin β subunits above, actin below, and directly or indirectly with other adhesion-component proteins. This positioning makes talin a central component in the dynamic process of cell adhesion formation and disassembly. As talin is important for forming adhesion complexes, an increase in talin stimulates the formation of more adhesion complexes. Paxillin, a phosphotyrosine protein linked to cell adhesion, acts as a docking protein that facilitates the attachment of signaling molecules to specific cellular compartments and/or attaches specific combinations of signaling molecules to complexes to coordinate downstream signals (25). This coordination is essential for effective cellular communication and response to changes in the extracellular environment.

In this study, DEGs revealed that most of the genes in the ECM receptor interaction pathway linked to the focal adhesion pathway, such as secreted phosphoprotein 1 (SPP1), collagen type I α1/α2 chain (COL1A1/COL1A2), collagen type VI α1/α2 chain (COL6A1/COL6A2), laminin subunit α3/γ1 (LAMA3/LAMC1), thrombospondin 4 (THBS4), and laminin subunit β-2-like (LOC125181834), were significantly upregulated. Additionally, integrin-related genes located on the cell membrane, like ITGA5 (LOC125181877), and integrin-associated protein genes in the cytoplasm, including talin (TLN1) and paxillin (PXN), were also upregulated. These results suggests that GA stimulates the formation of more adhesion complexes. Further, when integrins bind to focal adhesion kinase and tensin, various associated signaling factors converge, such as sarcoma (SRC) kinase, Rho, Ras-related C3 botulinum toxin substrate (Rac), the Ras-mitogen-activated protein kinase (Ras/MAPK) cascade, and cortical proteins, collectively triggering focal adhesion formation (21). This study showed that the upregulation of SRC, Rho GTPase activating protein 35 (ARHGAP35), and Rac family small GTPase 2 (RAC2) gene expression enhances focal adhesion formation. Growth factors are known to modulate adhesion and stimulate cell migration, such as epidermal growth factor (EGF) (26) and platelet-derived growth factor (PDGF) (27). In this study, GA treatment resulted in the upregulation of PDGF family A and D (PDGFA and PDGFD) genes, while EGF gene expression was downregulated. This mirrors findings in studies on focal segmental glomerulonephritis (FSGS), which upregulated the focal adhesion pathway, with key genes like fibronectin 1 (FN1) and protein phosphatase 1 (PP1) being upregulated, whereas EGF and insulin-like growth factor 1 (IGF1) were downregulated, stabilizing the cellular structure via enhanced adhesion to the glomerular basement membrane (28). Significant upregulation of genes like vav guanine nucleotide exchange factor 1 and 2 (VAV1 and VAV2), transcription factor Jun (JUN), and Cyclin D3 (CCND3) suggests that GA accelerated cell proliferation through focal adhesion. Collectively, GA may influence focal adhesion by enhancing local adhesion, which in turn could lead to hepatotoxicity through abnormalities in the focal adhesion pathway.

When goslings' liver cells became toxic due to abnormalities in the focal adhesion pathway, the overall liver cell metabolism was significantly disrupted. Compared with the control group, the gene expression pattern of the GA-treated group revealed notable downregulation in energy metabolism pathways. These include the core glycolysis/gluconeogenesis pathway, as well as the pyruvate metabolism, the TCA cycle, and propionate metabolism, all of which are essential for energy production. Although specific genes like hexokinase 3 (HK3) and aldolase C (ALDOC) were upregulated, there was significant downregulation of key genes involved in glycolysis. These include glucose-6-phosphate isomerase (GPI), phosphofructokinase, liver type (PFKL), and aldolase B (ALDOB), indicating an impaired ability to convert glucose into pyruvate. Notably, PFKL, a key metabolic enzyme in the glycolysis pathway, is responsible for catalyzing the conversion of fructose-6-phosphate to fructose-1,6-diphosphate, and its activity is tightly regulated by fructose-6-phosphate and ATP levels (29). PFKL also acts as a negative regulator of reactive oxygen species bursts in phagocytes, and its knockdown leads to an increase in glucose flux into the pentose phosphate pathway, which promotes NADPH production and neutrophil activation (30). Additionally, significant downregulation in the pyruvate dehydrogenase complex [pyruvate dehydrogenase E1α1 and β subunit (PDHA1 and PDHB), dihydrolipoamide S-acetyltransferase (DLAT), and dihydrolipoamide dehydrogenase (DLD)], lactate dehydrogenase B (LDHB), and acetaldehyde dehydrogenase (ALDH3A2 and ALDH7A1) slowed the entry of carbon into the TCA cycle, reducing pyruvate metabolism and propionate metabolism efficiency. Since glucose catabolism through aerobic pathways is the primary energy source for animal physiological activities, this impairment is highly detrimental. In the TCA cycle, reduced expression of succinate-CoA ligase subunit α (SUCLG1), succinate dehydrogenase complex flavoprotein subunit A (SDHA), and malate dehydrogenase 1 (MDH1) further suggested that the goslings' energy supply was compromised. The gluconeogenesis pathway was also significantly affected, with the downregulation of genes encoding phosphoenolpyruvate carboxykinase 2 (LOC125181711) and glucose-6-phosphatase catalytic subunit 1 (G6PC1). These enzymes play critical roles in glucose production: LOC125181711 converts oxaloacetate to phosphoenolpyruvate, and G6PC catalyzes the hydrolysis of glucose-6-phosphate to glucose. A reduction in these enzymes' expression suggests that GA hampers gluconeogenesis, impairing the goslings' ability to maintain stable blood glucose levels, further exacerbating metabolic stress. This significant disruption in both glycolysis and gluconeogenesis pathways indicates that GA not only impairs energy production but also severely hinders the ability of the liver to regulate essential metabolic processes, leading to compromised liver function and overall toxicity.

As the central organ responsible for lipid metabolism, the liver regulates lipid uptake, synthesis, oxidative catabolism, and export. Fatty acids must be converted to active forms, such as lipoyl CoA, before undergoing oxidative catabolism. The enzyme long-chain lipoyl CoA synthetase (ACSL) activates long fatty acid chains in peroxisomes, forming lipoyl CoA and directing the fatty acids toward β-oxidation with the participation of ATP, HSCoA, and Mg^2+^ (31). Fatty acid β-oxidation within the mitochondria is the primary pathway for fatty acid catabolism. In this study, GA significantly upregulated the expression of long-chain acyl-CoA synthetase-4 (ACSL4), a key enzyme in fatty acid metabolism. However, several other key enzymes involved in fatty acid β-oxidation, such as carnitine palmitoyltransferase 2 (CPT2), medium-chain acyl-CoA dehydrogenase (ACADM), long-chain acyl-CoA dehydrogenase (ACADL), enoyl-CoA hydratase short-chain 1 (ECHS1), enoyl-CoA hydratase and 3-hydroxy acyl CoA dehydrogenase (EHHADH), and acetyl-CoA acyltransferase 2 (ACAA2), were significantly downregulated. These results suggest that although ACSL4 was upregulated, critical components of the β-oxidation pathway were impaired, resulting in reduced fatty acid degradation. The downregulation of these key β-oxidation enzymes indicates that gossypol acetate hinders efficient fatty acid catabolism, which limits energy production and the generation of intermediate metabolites required for cell growth. ACSL4 has been linked to various metabolism-related diseases (32, 33), and elevated levels of ACSL4 expression are associated with increased hepatic fat content, as seen in studies of human liver fat (34). In mouse models of non-alcoholic steatohepatitis, they were targeting hepatic ACSL4 improved liver health, highlighting its role in metabolic diseases (35). This study aligns with these findings, suggesting that GA disrupts fatty acid metabolism, leading to metabolic stress and potential liver damage.

Peroxisome proliferator-activated receptors (PPARs) are ligand-activated transcription factors involved in various biological processes, including lipid metabolism, adipocyte differentiation, thermogenesis, cell survival, ubiquitination, and glycolysis/gluconeogenesis. In this study, GA administration notably altered the PPAR signaling pathway, significantly affecting lipid metabolism and energy regulation. GA upregulated genes related to lipogenesis (LOC106033870), cholesterol metabolism (LOC106039033), and fatty acid transporters (ACSL4, SLC27A1). However, it downregulated genes involved in fatty acid oxidation (CPT2, EHHADH, ACADL, ACADM, SCP2) and gluconeogenesis (LOC125181711, GK), suggesting a shift toward lipid storage and a reduction in energy production through fatty acid oxidation and glucose regulation. This metabolic alteration resulted in insufficient energy supply and uncontrolled glucose homeostasis, negatively impacting the organism's overall energy balance. Also, GA disrupted primary bile acid biosynthesis, critical for cholesterol metabolism and fat digestion. Bile acids are synthesized from cholesterol in hepatocytes, primarily via the classic (or neutral) pathway, where cholesterol is first converted to 7α-hydroxycholesterol by the rate-limiting enzyme cholesterol 7α-hydroxylase (LOC106039033), followed by multiple reactions leading to the formation of primary bile acids and their conjugated forms (36). GA upregulated the expression of LOC106039033 and 3-β-hydroxysteroid dehydrogenase type 7-like (LOC106041408), which are critical enzymes in the cholesterol conversion process. However, genes related to α-methylacyl-CoA racemase (AMACR), hydroxysteroid 17β dehydrogenase 4 (HSD17B4), and steroid carrier protein 2 (SCP2) were significantly downregulated, leading to impaired primary bile acid synthesis. This disruption extends to other pathways, such as the 24-hydroxylase and 25-hydroxylase pathways, affecting the liver's ability to process fats and cholesterol efficiently, thus impairing metabolic balance.

Metabolomics represents the final step in the “omics” cascade, offering a snapshot of the current metabolic state of a biological system and reflecting the complex interaction between environmental factors and the organism's genetic, transcriptional, and proteomic activities (37). LC-MS, especially in UPLC combined with high-resolution mass spectrometry, is a powerful tool for studying metabolite composition and its dynamic changes within living organisms. This technique is precious because it detects a broad range of substances with relatively simple pre-treatment requirements. In this study, non-targeted metabolomics techniques were employed alongside transcriptomics to analyze the liver metabolites of goslings and explore the toxic effects of GA on the liver. A total of 126 differential metabolites were identified between the GA50 and control groups, of which 80 metabolites were significantly upregulated, and 46 were significantly downregulated. This comprehensive metabolomic analysis provides insight into how GA disrupts metabolic processes in the liver, reflecting the alterations in the biochemical pathways that occur due to exposure to the compound. The metabolite changes identified in this study can potentially reveal the mechanisms of GA-induced hepatotoxicity.

Amino acids are vital for protein synthesis and are integral to several key metabolic processes, including energy production, hormone synthesis, neurotransmission, and functioning as intermediates in the TCA cycle and gluconeogenesis. This study demonstrated that the differential metabolites induced by GA administration were significantly enriched in the cAMP signaling pathway and serotonergic synapse, with serotonin emerging as a critical metabolic factor. At the transcriptional level, GA was found to disrupt tryptophan metabolism in the liver, and similarly, untargeted metabolomics revealed that GA administration decreased serotonin levels. Serotonin is synthesized from tryptophan through a two-step process: tryptophan is first hydroxylated to 5-hydroxytryptophan by tryptophan hydroxylase and then undergoes decarboxylation to form serotonin (38). Serotonin, an indole derivative, is an important neurotransmitter in the body. About 90% of serotonin is produced by enterochromaffin cells in the gastrointestinal tract and transported throughout the body (39). Most previous research has focused on serotonin's role in the central nervous system. Wang et al. (40) demonstrated that increased serotonin activity or affinity in central transmitters can enhance serotonin release, coupling it with G-protein-coupled receptors. This interaction converts ATP to cAMP, directly modulating the downstream pathways to activate the serotonergic synapse, thus increasing serotonin and γ-aminobutyric acid expression, which can improve insomnia and reduce anxiety. cAMP is also a typical second messenger that responds to the binding of extracellular signals to cell surface receptors through its concentration changes. It regulates the activity of intracellular enzymes and non-enzymatic proteins, thus functioning as a signal-carrying and amplifying agent in the cellular signaling pathway (40). Research has also shown that serotonin levels are significantly lower in patients with cirrhosis compared to healthy individuals (41), mainly due to a defect in the platelet storage pool, which impairs the storage of serotonin (42). Moreover, specific serotonin receptors have been shown to influence mitochondrial biogenesis in non-neuronal cells like renal proximal tubule cells and cardiomyocytes (43, 44). Serotonin enhances mitochondrial respiratory capacity, oxidative phosphorylation efficiency, and ATP production (45). Thus, the reduction of serotonin content in the liver caused by GA may be associated with mitochondrial damage and a subsequent decrease in ATP synthesis, potentially contributing to impaired liver function.

In the present study, the differential metabolites between the GA-treated and the control groups were notably enriched in lipid metabolism, including linoleic acid and arachidonic acid metabolism, with most metabolites being downregulated. These results were consistent with our previous study (10), where GA administration reduced serum lipid metabolism markers such as total cholesterol, triglycerides, high-density lipoprotein cholesterol, and low-density lipoprotein cholesterol. Linoleic acid, a core metabolite in the linoleic acid metabolism pathway, was present in the GA-treated group at only 20% of the concentration in the control group. This decrease also extended to its downstream product, 13S-hydroxyoctadecadienoic acid, a compound involved in anti-inflammatory processes. Similar to these findings, Li et al. (46) reported that recurrent inflammation and cholestasis due to intrahepatic bile duct stones in humans may be responsible for disrupting the linoleic acid metabolic pathway. Li et al. (46) reported that disruptions in the linoleic acid metabolic pathway due to recurrent inflammation and cholestasis in humans could impair the anti-inflammatory effects of 13S-hydroxyoctadecadienoic acid. Similarly, GA-induced impairment in primary bile acid biosynthesis in this study likely affected linoleic acid metabolism, thus reducing the anti-inflammatory potential of these metabolites. Furthermore, since linoleic acid serves as the precursor to arachidonic acid, any disturbances in its metabolism can impact the arachidonic acid pathway, which is crucial for producing inflammatory mediators such as prostaglandins, prostacyclins, and thromboxanes. The study also highlighted that GA down-regulated metabolites such as prostaglandin E2 and 8-isoprostane. The decline in 8-isoprostane, a marker of lipid peroxidation, corresponds with reduced malondialdehyde content in the liver, as observed in previous studies (1). Prostaglandin E2 offers protection against various forms of liver injury, including promoting regeneration post-hepatic resection (47, 48). Therefore, the decreased levels of prostaglandin E2 likely hindered liver repair in the GA-treated group, exacerbating hepatic damage. In agreement with transcriptomic data, metabolomic analysis also showed that GA administration resulted in the downregulation of 9-cis-retinoic acid, 13S-hydroxyoctadecadienoic acid, and α-dimorphecolic acid. This downregulation of key ligands further suppressed the PPAR signaling pathway, a pathway essential for regulating fatty acid oxidation and gluconeogenesis. As PPARs belong to the nuclear hormone receptor superfamily, reduced PPAR signaling inhibits these processes, contributing to insufficient energy supply and dysregulated glucose metabolism within the organism. Thus, the combined disruptions in lipid and glucose metabolism caused by GA treatment ultimately impair the liver's metabolic capacity and energy homeostasis.

## 5 Conclusions

Overall, GA administration induced significant hepatocyte injury, with mitochondria emerging as the most prominently damaged organelles within the liver cells. Both transcriptomic and metabolomic analyses confirmed that GA treatment led to abnormalities in liver focal adhesion and the downregulation of critical metabolic pathways, including glycolysis/gluconeogenesis, the PPAR signaling pathway, and lipid metabolism. These disruptions resulted in a critical imbalance in the body's energy supply, further impairing the liver's metabolic function and overall cellular energy homeostasis.

## Data Availability

The data used in this study are publicly accessible through the NCBI online repository (Accession number: PRJNA1180732, PRJNA1181958, PRJNA1181960, and PRJNA1181962).
