# Supplementary material for: Comprehensive analysis of histophysiology, transcriptomics and metabolomics in goslings exposed to gossypol acetate: unraveling hepatotoxic mechanisms
Source: Front Vet Sci. 2025 Jan 21;12:1527284. doi: 10.3389/fvets.2025.1527284 (PMC11792171; doi:10.3389/fvets.2025.1527284)
Supplement: Supplementary file 1 [file Data_Sheet_1.zip › supplementary materials/Figure S2. Quantitative validation of some differentially expressed genes identified by liver transcriptome analysis..docx]

**Figure S2.** Quantitative validation of some differentially expressed genes identified by liver transcriptome analysis.
