# Supplementary material for: Comprehensive analysis of histophysiology, transcriptomics and metabolomics in goslings exposed to gossypol acetate: unraveling hepatotoxic mechanisms
Source: Front Vet Sci. 2025 Jan 21;12:1527284. doi: 10.3389/fvets.2025.1527284 (PMC11792171; doi:10.3389/fvets.2025.1527284)
Supplement: Supplementary file 1 [file Data_Sheet_1.zip › supplementary materials/Table S2. Primers used for quantitative real-time PCR analysis.docx]

**Table S2.** Primers used for quantitative real-time PCR analysis.

| Gene name | Primer sequence (5′–3′) | Product size (bp) | Gene Bank No. |
| --- | --- | --- | --- |
| ANXA2 | F:CACACCTGCAGAAAGTGTTTGA | 200 | XM_013177447.2 |
|  | R:GATCAGGACCTTGTCACGGG |  |  |
| PSTPIP1 | F:AGAGGTTCGGGTAAGCTTGG | 263 | XM_013194117.2 |
|  | R:AGACCCCATCTGCTTTCTCAAG |  |  |
| IL7R | F:TGGAGAAACGGGAAGACGTG | 137 | XM_013181777.2 |
|  | R:TGGTACCTCGGGCTTAACAAT |  |  |
| CCN1 | F:GAGTGGGTCTGCGATGAGAG | 161 | XM_048059279.1 |
|  | R:TCGGATCCAAACACAGGTAGC |  |  |
| SLC38A10 | F:GGAGATGATTCGCGTGGGAT | 228 | XM_048050304.1 |
|  | R:AGGACTGTTTCCACGTTGGG |  |  |
| LOC106044595 | F:CCTGGGAGTGAAAAACATCGC | 166 | XM_013194697.2 |
|  | R:CCAGTCACTTACACCACCTTTG |  |  |
| β-actin | F:GCACCCAGCACGATGAAAAT | 150 | XM_013174886.1 |
|  | R:GACAATGGAGGGTCCGGATT |  |  |
