# Supplementary material for: Comprehensive analysis of histophysiology, transcriptomics and metabolomics in goslings exposed to gossypol acetate: unraveling hepatotoxic mechanisms
Source: Front Vet Sci. 2025 Jan 21;12:1527284. doi: 10.3389/fvets.2025.1527284 (PMC11792171; doi:10.3389/fvets.2025.1527284)
Supplement: Supplementary file 1 [file Data_Sheet_1.zip › supplementary materials/Figure S1. The melt curves and melt peaks of the primers..docx]

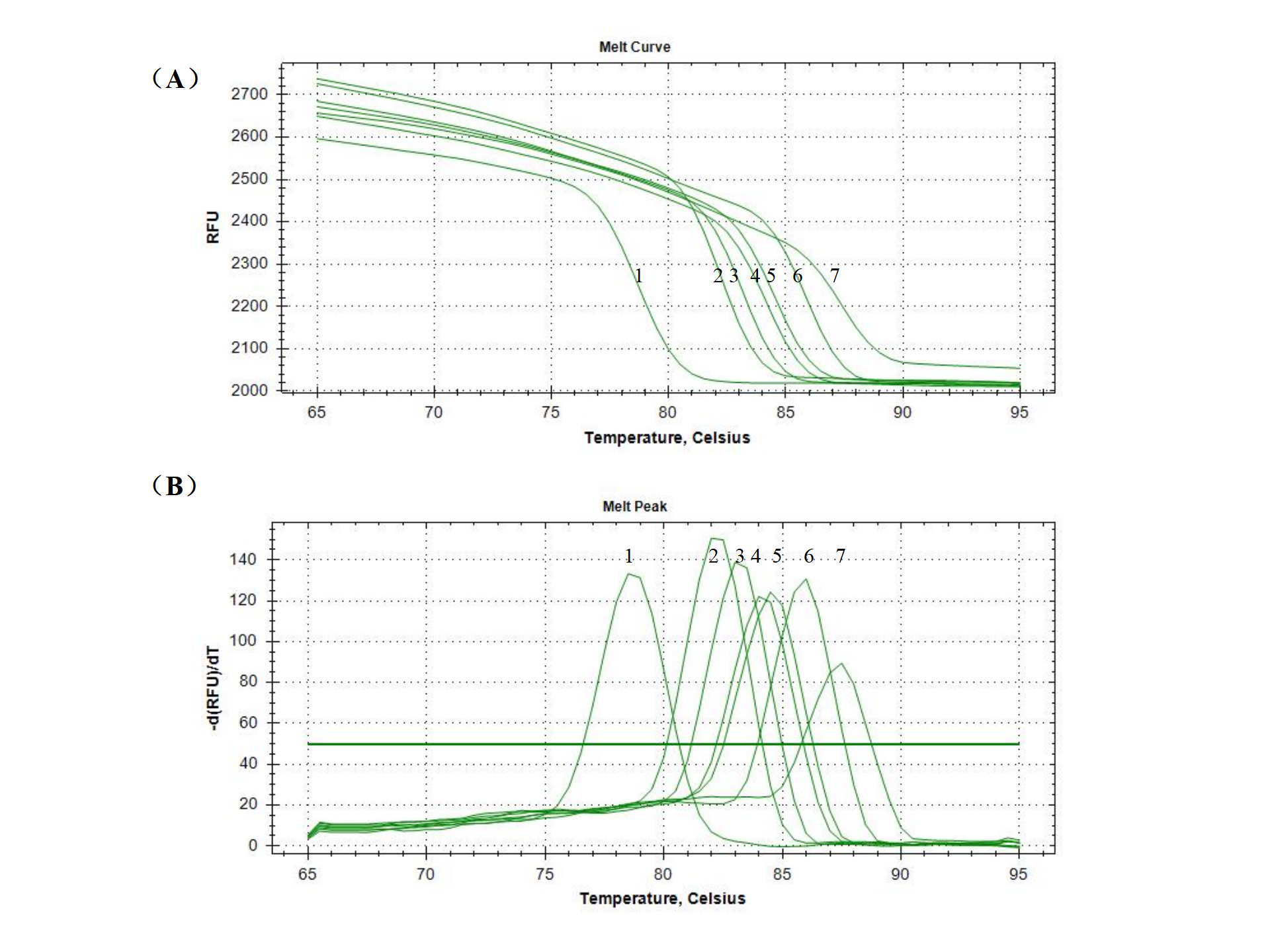


**Figure S1.** The melt curves (A) and melt peaks (B) of the primers. The primers in numerical order were *IL7R*, *ANXA2*, *PSTPIP1*, *LOC106044595*, *CCN1*, *β-actin* and *SLC38A10*.
