# Supplementary material for: Comprehensive analysis of histophysiology, transcriptomics and metabolomics in goslings exposed to gossypol acetate: unraveling hepatotoxic mechanisms
Source: Front Vet Sci. 2025 Jan 21;12:1527284. doi: 10.3389/fvets.2025.1527284 (PMC11792171; doi:10.3389/fvets.2025.1527284)
Supplement: Supplementary file 1 [file Data_Sheet_1.zip › supplementary materials/Table S4. List of differentially expressed genes in the liver of GA50 and control goslings.docx]

**Table S4.** List of differentially expressed genes in the liver of GA50 and control goslings.

| gene_name | GA50 | CON | log_2_FoldChange | pvalue | padj |
| --- | --- | --- | --- | --- | --- |
| *LOC106049823* | 218.65 | 633.86 | -1.54 | 2.03E-09 | 2.80E-05 |
| *IL7R* | 25.84 | 0.00 | 7.33 | 5.64E-08 | 0.000388017 |
| *LOC106044595* | 1000.86 | 2741.26 | -1.45 | 1.05E-07 | 0.000482257 |
| *BTG2* | 871.39 | 390.28 | 1.16 | 1.74E-07 | 0.000597586 |
| *SLC38A10* | 244.69 | 66.61 | 1.88 | 2.77E-07 | 0.000737325 |
| *EPHX2* | 879.91 | 1800.12 | -1.03 | 3.21E-07 | 0.000737325 |
| *TDRD15* | 1.67 | 45.75 | -4.95 | 6.37E-07 | 0.001130612 |
| *LOC106039033* | 105.87 | 16.13 | 2.71 | 7.28E-07 | 0.001130612 |
| *LOC106038117* | 48.23 | 1.35 | 5.17 | 7.39E-07 | 0.001130612 |
| *CCN1* | 65.58 | 3.23 | 4.31 | 9.61E-07 | 0.001322344 |
| *NR0B2* | 147.30 | 414.05 | -1.49 | 2.47E-06 | 0.002804868 |
| *PPDPFL* | 4680.40 | 11252.90 | -1.27 | 2.58E-06 | 0.002804868 |
| *SPECC1* | 59.01 | 4.74 | 3.62 | 2.65E-06 | 0.002804868 |
| *JCHAIN* | 623.02 | 142.56 | 2.13 | 3.33E-06 | 0.003099723 |
| *LOC106037570* | 3140.78 | 7186.84 | -1.19 | 3.38E-06 | 0.003099723 |
| *LOC106049124* | 736.74 | 166.49 | 2.15 | 4.86E-06 | 0.004080214 |
| *TMEM269* | 20.23 | 0.00 | 6.96 | 5.04E-06 | 0.004080214 |
| *LOC106049915* | 163.75 | 19.30 | 3.09 | 1.18E-05 | 0.008997735 |
| *LYPD6B* | 19.61 | 0.23 | 6.21 | 1.34E-05 | 0.009724521 |
| *LOC125182982* | 63.68 | 2.91 | 4.43 | 1.51E-05 | 0.010420334 |
| *NRSN1* | 162.46 | 50.56 | 1.68 | 1.74E-05 | 0.010936616 |
| *ANXA2* | 216.27 | 75.33 | 1.52 | 1.75E-05 | 0.010936616 |
| *CUNH2orf49* | 72.97 | 319.73 | -2.13 | 2.01E-05 | 0.011380889 |
| *P4HA3* | 33.66 | 0.78 | 5.58 | 2.03E-05 | 0.011380889 |
| *LOC106047137* | 17.05 | 0.00 | 6.72 | 2.07E-05 | 0.011380889 |
| *PDP2* | 76.47 | 184.22 | -1.27 | 2.56E-05 | 0.013580244 |
| *AMACR* | 573.89 | 1198.40 | -1.06 | 3.08E-05 | 0.015293808 |
| *LOC106049430* | 218.00 | 54.59 | 2.01 | 3.11E-05 | 0.015293808 |
| *PSTPIP1* | 47.01 | 6.62 | 2.82 | 3.37E-05 | 0.015974876 |
| *LOC125181225* | 2203.58 | 48.39 | 5.51 | 4.07E-05 | 0.018663257 |
| *PACSIN2* | 271.80 | 765.27 | -1.49 | 4.98E-05 | 0.022095159 |
| *LOC106048960* | 63.53 | 5.95 | 3.41 | 6.01E-05 | 0.024885014 |
| *SLC41A3* | 333.72 | 145.87 | 1.19 | 6.13E-05 | 0.024885014 |
| *GGT5* | 196.56 | 69.86 | 1.50 | 6.15E-05 | 0.024885014 |
| *LOC106040614* | 25.87 | 1.16 | 4.53 | 6.55E-05 | 0.025765461 |
| *IL22RA1* | 13.42 | 0.00 | 6.37 | 7.73E-05 | 0.029166728 |
| *LOC125184398* | 3.08 | 42.49 | -3.84 | 7.84E-05 | 0.029166728 |
| *LOC125180625* | 762.98 | 269.38 | 1.50 | 8.42E-05 | 0.029796566 |
| *ACSS3* | 680.34 | 1415.32 | -1.06 | 8.44E-05 | 0.029796566 |
| *RET* | 47.23 | 4.82 | 3.31 | 9.09E-05 | 0.031235248 |
| *RAB11FIP5* | 209.83 | 90.97 | 1.21 | 9.30E-05 | 0.031235248 |
| *MAP7D2* | 249.79 | 484.57 | -0.95 | 0.000111171 | 0.035034486 |
| *NMNAT2* | 28.88 | 1.96 | 3.88 | 0.00011186 | 0.035034486 |
| *ITPK1* | 294.32 | 139.81 | 1.07 | 0.000111972 | 0.035034486 |
| *SPP1* | 128.68 | 32.30 | 2.00 | 0.00011874 | 0.03583535 |
| *NDRG1* | 3202.35 | 7262.29 | -1.18 | 0.000125439 | 0.03583535 |
| *EZR* | 180.67 | 38.15 | 2.24 | 0.000128056 | 0.03583535 |
| *GGACT* | 138.87 | 395.50 | -1.51 | 0.000129416 | 0.03583535 |
| *RPP40* | 75.94 | 185.46 | -1.29 | 0.000129826 | 0.03583535 |
| *RGS1* | 62.36 | 7.01 | 3.19 | 0.000130149 | 0.03583535 |
| *TLCD4* | 119.83 | 313.19 | -1.38 | 0.00013436 | 0.036044318 |
| *LOC106038759* | 15.42 | 0.00 | 6.58 | 0.000136145 | 0.036044318 |
| *LOC106033870* | 3254.93 | 1848.34 | 0.82 | 0.000139481 | 0.03623083 |
| *MAT2A* | 110.01 | 239.17 | -1.12 | 0.000145291 | 0.037041146 |
| *LOC125184234* | 13.70 | 0.00 | 6.40 | 0.00015636 | 0.039138252 |
| *RASD1* | 282.20 | 110.68 | 1.35 | 0.000164135 | 0.040350879 |
| *LDHB* | 3132.71 | 7880.87 | -1.33 | 0.000167756 | 0.04051749 |
| *LOC106046477* | 17.06 | 0.64 | 4.71 | 0.000208122 | 0.049400247 |
| *C7* | 441.47 | 142.15 | 1.64 | 0.000214013 | 0.049937486 |
| *PDZK1* | 143.00 | 43.84 | 1.70 | 0.000227156 | 0.051795757 |
| *ALDOB* | 100169.83 | 175972.76 | -0.81 | 0.000232092 | 0.051795757 |
| *EVL* | 135.67 | 40.43 | 1.76 | 0.00023351 | 0.051795757 |
| *LOC106032597* | 12.68 | 0.23 | 5.57 | 0.00023764 | 0.051795757 |
| *NIT2* | 398.36 | 886.13 | -1.16 | 0.000246693 | 0.051795757 |
| *LOC125180682* | 825.44 | 2477.75 | -1.59 | 0.000249749 | 0.051795757 |
| *HNF1B* | 123.17 | 19.67 | 2.65 | 0.00025046 | 0.051795757 |
| *CPT2* | 484.15 | 902.96 | -0.90 | 0.00025653 | 0.051795757 |
| *LOC106040078* | 165.33 | 378.11 | -1.20 | 0.000256718 | 0.051795757 |
| *AATF* | 69.35 | 166.69 | -1.27 | 0.000263324 | 0.051795757 |
| *CCDC78* | 26.58 | 134.03 | -2.33 | 0.000263362 | 0.051795757 |
| *CSNK1G1* | 54.59 | 14.89 | 1.88 | 0.000284986 | 0.055259152 |
| *LOC106049821* | 171.05 | 54.79 | 1.64 | 0.000297139 | 0.056752114 |
| *HACL1* | 389.35 | 729.36 | -0.90 | 0.00030093 | 0.056752114 |
| *LANCL3* | 10.38 | 0.00 | 6.01 | 0.000320946 | 0.059708984 |
| *LOC106049126* | 98.56 | 18.54 | 2.42 | 0.000326345 | 0.059750584 |
| *LOC106043780* | 10.33 | 0.00 | 6.00 | 0.00032985 | 0.059750584 |
| *SI* | 0.60 | 19.80 | -5.13 | 0.000341092 | 0.060751996 |
| *NCS1* | 47.03 | 7.57 | 2.62 | 0.000344204 | 0.060751996 |
| *PLAAT1* | 0.00 | 16.84 | -6.39 | 0.000353921 | 0.061676316 |
| *MRPL48* | 122.21 | 257.60 | -1.07 | 0.000377812 | 0.065016663 |
| *LOC106033185* | 2568.56 | 4682.52 | -0.87 | 0.000390544 | 0.066378075 |
| *LOC106041090* | 270.01 | 99.34 | 1.44 | 0.000396442 | 0.066558711 |
| *EXOSC2* | 103.73 | 218.12 | -1.07 | 0.000402836 | 0.066817336 |
| *SIDT1* | 58.79 | 6.33 | 3.20 | 0.000414572 | 0.067945308 |
| *GNG4* | 51.85 | 7.75 | 2.72 | 0.000435926 | 0.07060462 |
| *LSP1* | 280.30 | 124.98 | 1.17 | 0.000443427 | 0.070984364 |
| *RABGGTB* | 450.11 | 818.27 | -0.86 | 0.000475296 | 0.074827691 |
| *EHHADH* | 608.39 | 1443.04 | -1.25 | 0.000478306 | 0.074827691 |
| *LRR1* | 986.81 | 1946.49 | -0.98 | 0.000493093 | 0.076274307 |
| *DLAT* | 1145.28 | 2170.87 | -0.92 | 0.000505619 | 0.077342858 |
| *BCL7A* | 138.76 | 285.23 | -1.04 | 0.000531213 | 0.080364956 |
| *PDGFA* | 251.55 | 72.64 | 1.80 | 0.000556348 | 0.083252689 |
| *ACOT11* | 146.57 | 56.03 | 1.39 | 0.000607422 | 0.08991798 |
| *LOC106045795* | 404.52 | 128.18 | 1.66 | 0.000632312 | 0.092273125 |
| *B3GNT5* | 33.90 | 4.73 | 2.81 | 0.000638923 | 0.092273125 |
| *LOC106032071* | 164.37 | 76.69 | 1.10 | 0.000643439 | 0.092273125 |
| *SIMC1* | 1.99 | 22.98 | -3.66 | 0.000653778 | 0.092789296 |
| *FHL3* | 206.74 | 83.67 | 1.31 | 0.000677269 | 0.095142466 |
| *RNF213* | 272.41 | 108.98 | 1.32 | 0.000685303 | 0.09529862 |
| *PRXL2A* | 316.80 | 795.40 | -1.33 | 0.000712724 | 0.098120655 |
| *LOC125184635* | 8395.01 | 14760.72 | -0.81 | 0.000752068 | 0.102512115 |
| *CUNH12orf73* | 51.05 | 120.20 | -1.23 | 0.000763615 | 0.103065613 |
| *ST8SIA6* | 19.83 | 76.03 | -1.94 | 0.000798331 | 0.106705128 |
| *LOC125180885* | 337.70 | 161.82 | 1.06 | 0.000858623 | 0.11366027 |
| *STC2* | 394.26 | 125.38 | 1.65 | 0.000878287 | 0.115155965 |
| *PDHB* | 2162.12 | 3811.67 | -0.82 | 0.000892672 | 0.115599798 |
| *RNF152* | 142.94 | 40.77 | 1.81 | 0.000902213 | 0.115599798 |
| *KASH5* | 0.30 | 16.62 | -5.65 | 0.000906863 | 0.115599798 |
| *NSG1* | 11.07 | 0.00 | 6.10 | 0.000937004 | 0.118346211 |
| *AHSG* | 9956.35 | 20042.21 | -1.01 | 0.000969677 | 0.121359454 |
| *MRPS27* | 146.72 | 280.76 | -0.94 | 0.001006216 | 0.124797941 |
| *PTPRU* | 168.28 | 61.60 | 1.44 | 0.001031596 | 0.126803407 |
| *OSGIN2* | 65.60 | 23.51 | 1.48 | 0.001080409 | 0.131628205 |
| *ASPSCR1* | 188.98 | 429.34 | -1.19 | 0.001102901 | 0.132752723 |
| *SLC16A6* | 35.76 | 4.36 | 3.05 | 0.001108924 | 0.132752723 |
| *LOC125180016* | 0.00 | 9.41 | -5.55 | 0.001126228 | 0.132911179 |
| *RUNX3* | 61.33 | 18.22 | 1.75 | 0.001139633 | 0.132911179 |
| *TLN1* | 452.95 | 226.20 | 1.00 | 0.001146618 | 0.132911179 |
| *HOMER1* | 12.26 | 0.00 | 6.26 | 0.001149212 | 0.132911179 |
| *LOC106039471* | 0.00 | 10.50 | -5.70 | 0.00115852 | 0.132911179 |
| *NPDC1* | 147.33 | 58.36 | 1.33 | 0.001181792 | 0.134460561 |
| *LOC106047695* | 1238.44 | 1996.62 | -0.69 | 0.001192862 | 0.134607666 |
| *LOC106033340* | 17.91 | 0.45 | 5.32 | 0.001226786 | 0.13731027 |
| *RRAD* | 8.27 | 0.00 | 5.68 | 0.00127879 | 0.141237077 |
| *VGLL3* | 10.56 | 0.00 | 6.04 | 0.001291602 | 0.141237077 |
| *FGFR3* | 422.34 | 143.59 | 1.56 | 0.001300745 | 0.141237077 |
| *LOC125182135* | 10.55 | 0.00 | 6.03 | 0.001302906 | 0.141237077 |
| *PROSER2* | 210.67 | 82.86 | 1.35 | 0.001341872 | 0.144324671 |
| *TENT5C* | 27.47 | 2.66 | 3.35 | 0.001389158 | 0.146828365 |
| *PDHA1* | 472.47 | 934.41 | -0.99 | 0.001390987 | 0.146828365 |
| *SEPTIN9* | 249.16 | 124.80 | 1.00 | 0.001397146 | 0.146828365 |
| *NQO1* | 150.92 | 481.82 | -1.68 | 0.001428601 | 0.148829503 |
| *LOC106033760* | 4734.84 | 8381.62 | -0.82 | 0.00143781 | 0.148829503 |
| *ACAD11* | 489.24 | 907.80 | -0.89 | 0.001507541 | 0.153802828 |
| *AKT1* | 205.64 | 349.62 | -0.77 | 0.001524272 | 0.153802828 |
| *LOC106047531* | 127.23 | 26.60 | 2.26 | 0.001538895 | 0.153802828 |
| *IGLL1* | 2822.53 | 421.64 | 2.74 | 0.001541349 | 0.153802828 |
| *LOC106039386* | 1007.93 | 1765.94 | -0.81 | 0.001556675 | 0.153802828 |
| *KYNU* | 270.85 | 572.98 | -1.08 | 0.001560729 | 0.153802828 |
| *FGL2* | 152.87 | 60.06 | 1.36 | 0.001564059 | 0.153802828 |
| *LOC106037140* | 18.17 | 1.13 | 4.03 | 0.001577124 | 0.153987691 |
| *LOC106037597* | 34.70 | 3.61 | 3.24 | 0.001689785 | 0.163296642 |
| *LOC106049913* | 360.04 | 45.74 | 2.98 | 0.001696188 | 0.163296642 |
| *NIPAL2* | 8.70 | 0.00 | 5.76 | 0.001724672 | 0.164885815 |
| *LOC125181878* | 59.84 | 683.47 | -3.51 | 0.001753452 | 0.166481169 |
| *LOC125180698* | 47.01 | 134.77 | -1.50 | 0.001772918 | 0.167176417 |
| *CASKIN2* | 54.85 | 16.09 | 1.76 | 0.001785324 | 0.167201026 |
| *IRF8* | 358.87 | 187.25 | 0.94 | 0.001806235 | 0.16801647 |
| *DHX29* | 112.47 | 229.02 | -1.03 | 0.001821005 | 0.168253502 |
| *CXCR6* | 63.67 | 17.83 | 1.84 | 0.001839596 | 0.168838075 |
| *LOC106033377* | 593.17 | 1046.55 | -0.82 | 0.001874136 | 0.170869044 |
| *MAB21L4* | 14.15 | 0.94 | 3.96 | 0.001890739 | 0.171248708 |
| *CAPRIN2* | 7.84 | 0.00 | 5.61 | 0.001958003 | 0.176181872 |
| *IKZF1* | 39.81 | 8.67 | 2.20 | 0.0019868 | 0.17715833 |
| *FHL2* | 201.74 | 65.76 | 1.62 | 0.001998526 | 0.17715833 |
| *MDH1* | 867.28 | 1405.16 | -0.70 | 0.00200746 | 0.17715833 |
| *LEAP2* | 211.71 | 430.96 | -1.03 | 0.002065268 | 0.18082777 |
| *SEPTIN5* | 324.63 | 140.00 | 1.21 | 0.002100043 | 0.18082777 |
| *GCLC* | 907.11 | 1879.05 | -1.05 | 0.002100272 | 0.18082777 |
| *LOC125179698* | 127.43 | 52.71 | 1.28 | 0.002101579 | 0.18082777 |
| *PLEKHA2* | 132.17 | 58.07 | 1.19 | 0.002130893 | 0.181649889 |
| *DZIP1* | 68.14 | 14.25 | 2.24 | 0.002139494 | 0.181649889 |
| *ACACB* | 8.48 | 0.00 | 5.71 | 0.002174466 | 0.181649889 |
| *NPNT* | 63.48 | 19.37 | 1.71 | 0.002184921 | 0.181649889 |
| *MYCL* | 1.69 | 18.97 | -3.53 | 0.002185672 | 0.181649889 |
| *LOC106048290* | 61.69 | 11.56 | 2.44 | 0.002192854 | 0.181649889 |
| *LOC106049914* | 497.28 | 50.05 | 3.31 | 0.002203496 | 0.181649889 |
| *MC5R* | 1316.00 | 718.22 | 0.87 | 0.002306981 | 0.187932078 |
| *RHEB* | 725.21 | 1278.76 | -0.82 | 0.002307004 | 0.187932078 |
| *ABHD3* | 7.68 | 37.84 | -2.26 | 0.002326672 | 0.188052056 |
| *LOC125182799* | 1.29 | 15.56 | -3.71 | 0.002343407 | 0.188052056 |
| *MLST8* | 27.51 | 85.20 | -1.62 | 0.002366735 | 0.188052056 |
| *LMOD1* | 310.36 | 102.52 | 1.60 | 0.002377612 | 0.188052056 |
| *LOC106039282* | 7.87 | 0.00 | 5.60 | 0.002385507 | 0.188052056 |
| *BHLHA15* | 8.90 | 0.23 | 5.06 | 0.002390434 | 0.188052056 |
| *SMCR8* | 100.61 | 37.40 | 1.43 | 0.00243782 | 0.189461718 |
| *POU6F1* | 11.04 | 0.23 | 5.38 | 0.0024423 | 0.189461718 |
| *LECT2* | 4521.99 | 1921.69 | 1.23 | 0.002449639 | 0.189461718 |
| *GRB10* | 153.99 | 281.12 | -0.87 | 0.002488809 | 0.191415867 |
| *LOC106048984* | 216.49 | 59.57 | 1.86 | 0.002597596 | 0.198123367 |
| *CLU* | 215.14 | 68.46 | 1.66 | 0.002604803 | 0.198123367 |
| *MLKL* | 9.20 | 0.00 | 5.84 | 0.0026551 | 0.198337803 |
| *LOC106049654* | 90.47 | 33.23 | 1.44 | 0.002664946 | 0.198337803 |
| *PPL* | 10.19 | 0.00 | 5.99 | 0.002666315 | 0.198337803 |
| *LOC125180956* | 94.71 | 293.85 | -1.63 | 0.002677027 | 0.198337803 |
| *SLC7A3* | 9.59 | 38.42 | -2.02 | 0.002679657 | 0.198337803 |
| *ZMIZ2* | 243.79 | 115.12 | 1.08 | 0.002696205 | 0.198495506 |
| *LOC106030514* | 7.39 | 0.00 | 5.52 | 0.002716174 | 0.198901981 |
| *PAPSS2* | 300.08 | 154.37 | 0.95 | 0.002747363 | 0.199467661 |
| *CORO2A* | 36.63 | 6.65 | 2.46 | 0.002752877 | 0.199467661 |
| *SOX11* | 17.17 | 1.37 | 3.59 | 0.002808514 | 0.20112305 |
| *LOC125179809* | 11.93 | 0.00 | 6.21 | 0.002825279 | 0.20112305 |
| *PPP1R14D* | 68.36 | 168.10 | -1.30 | 0.002828199 | 0.20112305 |
| *EREG* | 7.24 | 30.03 | -2.08 | 0.002834189 | 0.20112305 |
| *PSPH* | 10452.01 | 24161.78 | -1.21 | 0.002885834 | 0.20112305 |
| *LOC106049361* | 3301.71 | 2155.10 | 0.62 | 0.002890834 | 0.20112305 |
| *CASTOR2* | 37.62 | 9.52 | 1.98 | 0.002895283 | 0.20112305 |
| *DPEP1* | 172.72 | 44.12 | 1.97 | 0.002901456 | 0.20112305 |
| *CUNH12orf75* | 241.30 | 109.63 | 1.14 | 0.002919825 | 0.20112305 |
| *KLF6* | 62.93 | 22.83 | 1.46 | 0.002921814 | 0.20112305 |
| *LOC125183060* | 7.77 | 0.00 | 5.58 | 0.00295952 | 0.202705007 |
| *TMEM156* | 91.18 | 24.41 | 1.91 | 0.002996535 | 0.203998715 |
| *LOC106049157* | 7.23 | 0.00 | 5.49 | 0.003031832 | 0.203998715 |
| *ACADL* | 1599.73 | 2868.78 | -0.84 | 0.00304754 | 0.203998715 |
| *NR0B1* | 24.53 | 72.18 | -1.53 | 0.003051223 | 0.203998715 |
| *SDCBP2* | 240.24 | 70.03 | 1.78 | 0.003052498 | 0.203998715 |
| *FOS* | 48.87 | 12.53 | 1.99 | 0.003109573 | 0.204171738 |
| *DHFR* | 365.20 | 659.54 | -0.85 | 0.00311053 | 0.204171738 |
| *LHFPL5* | 30.76 | 3.70 | 3.10 | 0.003113644 | 0.204171738 |
| *LOC106036868* | 9.85 | 0.00 | 5.92 | 0.003114409 | 0.204171738 |
| *SLC5A9* | 31.19 | 3.94 | 3.00 | 0.003211931 | 0.208788238 |
| *VEPH1* | 8.89 | 0.00 | 5.78 | 0.003228464 | 0.208788238 |
| *EYA1* | 46.22 | 4.84 | 3.27 | 0.003230326 | 0.208788238 |
| *GSTO1* | 197.05 | 384.68 | -0.97 | 0.003362585 | 0.216321087 |
| *LOC125180693* | 14.86 | 1.23 | 3.67 | 0.003417162 | 0.218809614 |
| *PKP2* | 78.21 | 23.80 | 1.72 | 0.003488935 | 0.222371164 |
| *UBE2QL1* | 7.22 | 0.00 | 5.49 | 0.003540319 | 0.222400338 |
| *ABCG8* | 17.92 | 64.83 | -1.88 | 0.003546649 | 0.222400338 |
| *MXRA5* | 4.05 | 29.48 | -2.81 | 0.003556121 | 0.222400338 |
| *SEMA3F* | 111.69 | 42.33 | 1.40 | 0.003557457 | 0.222400338 |
| *RTKN* | 95.60 | 28.35 | 1.75 | 0.003570166 | 0.222400338 |
| *EGLN3* | 664.78 | 1021.61 | -0.62 | 0.00363388 | 0.225349637 |
| *rna-NC_023832.1_2303..3912* | 207838.15 | 61234.07 | 1.76 | 0.003680858 | 0.227239313 |
| *SUN2* | 41.68 | 12.19 | 1.77 | 0.003807633 | 0.234016455 |
| *LOC106031293* | 111.67 | 51.31 | 1.13 | 0.003892871 | 0.237743523 |
| *ATP10A* | 11.21 | 0.64 | 4.12 | 0.003907481 | 0.237743523 |
| *LOC106029573* | 1408.42 | 796.55 | 0.82 | 0.003932086 | 0.237743523 |
| *SOX9* | 144.49 | 45.12 | 1.69 | 0.003954156 | 0.237743523 |
| *LOC106045149* | 21.01 | 2.26 | 3.20 | 0.003967685 | 0.237743523 |
| *LDB2* | 92.03 | 181.05 | -0.98 | 0.00397189 | 0.237743523 |
| *VPS29* | 210.82 | 358.64 | -0.77 | 0.00398935 | 0.237754918 |
| *WNT16* | 8.70 | 0.00 | 5.76 | 0.004071804 | 0.241551055 |
| *CD24* | 81.82 | 24.63 | 1.74 | 0.004088138 | 0.241551055 |
| *NAGA* | 420.36 | 145.24 | 1.53 | 0.004143539 | 0.243778221 |
| *HSD17B4* | 3204.32 | 5226.64 | -0.71 | 0.004225988 | 0.24745897 |
| *LOC125180957* | 20.20 | 0.90 | 4.49 | 0.004242051 | 0.24745897 |
| *DEF6* | 41.19 | 11.14 | 1.89 | 0.004289966 | 0.249198137 |
| *LOC106029505* | 41.18 | 108.76 | -1.40 | 0.004309077 | 0.249256563 |
| *HEXIM1* | 471.52 | 287.85 | 0.71 | 0.004351795 | 0.250674332 |
| *ARID3C* | 11.06 | 0.00 | 6.09 | 0.004470617 | 0.255767986 |
| *FIGNL1* | 13.67 | 1.16 | 3.60 | 0.00447738 | 0.255767986 |
| *SMARCC1* | 300.62 | 161.86 | 0.89 | 0.004529697 | 0.25768737 |
| *REG4* | 48.87 | 5.84 | 3.06 | 0.004575005 | 0.258513966 |
| *NCKAP1L* | 47.79 | 12.61 | 1.93 | 0.004605609 | 0.258513966 |
| *SLC7A5* | 155.68 | 81.57 | 0.94 | 0.004629542 | 0.258513966 |
| *GK* | 1711.26 | 2927.05 | -0.77 | 0.004630374 | 0.258513966 |
| *S1PR4* | 29.27 | 4.02 | 2.88 | 0.004638116 | 0.258513966 |
| *LOC106035754* | 33.09 | 131.69 | -2.00 | 0.004676671 | 0.259611807 |
| *HAAO* | 547.87 | 899.09 | -0.72 | 0.004696795 | 0.259681836 |
| *LOC106037917* | 1037.48 | 1620.69 | -0.64 | 0.004777815 | 0.261816605 |
| *PMM2* | 110.95 | 231.66 | -1.05 | 0.004784557 | 0.261816605 |
| *LOC106045206* | 7.65 | 0.26 | 4.84 | 0.004804459 | 0.261816605 |
| *ZBTB16* | 194.45 | 74.94 | 1.38 | 0.004827391 | 0.261816605 |
| *VILL* | 15.50 | 0.68 | 4.54 | 0.00484996 | 0.261816605 |
| *HLF* | 92.27 | 211.11 | -1.20 | 0.004859192 | 0.261816605 |
| *STK17A* | 170.42 | 75.85 | 1.17 | 0.004889571 | 0.261816605 |
| *LOC125182488* | 7.28 | 0.00 | 5.48 | 0.004894072 | 0.261816605 |
| *CLIC3* | 8.15 | 0.00 | 5.65 | 0.004906565 | 0.261816605 |
| *KIF21A* | 368.78 | 685.92 | -0.90 | 0.004931607 | 0.262136811 |
| *MYPN* | 12.86 | 51.64 | -1.99 | 0.005032411 | 0.264862947 |
| *NLE1* | 57.51 | 124.10 | -1.11 | 0.005033104 | 0.264862947 |
| *GNG7* | 396.78 | 230.59 | 0.79 | 0.005051362 | 0.264862947 |
| *DEGS2* | 19.91 | 1.58 | 3.68 | 0.00505985 | 0.264862947 |
| *LFNG* | 86.05 | 32.06 | 1.44 | 0.005095213 | 0.265703758 |
| *SIGLEC1* | 69.75 | 23.86 | 1.55 | 0.005116395 | 0.265801526 |
| *CXCR4* | 57.07 | 10.78 | 2.40 | 0.00517087 | 0.265913966 |
| *HSD17B1* | 8.20 | 0.00 | 5.67 | 0.00517314 | 0.265913966 |
| *RAPGEF1* | 137.58 | 57.97 | 1.25 | 0.005176505 | 0.265913966 |
| *RING1* | 556.71 | 279.90 | 0.99 | 0.005208285 | 0.265930159 |
| *CXCL12* | 652.33 | 292.54 | 1.16 | 0.005215453 | 0.265930159 |
| *DCDC2* | 96.10 | 37.07 | 1.38 | 0.005239136 | 0.266151997 |
| *AIF1L* | 1996.62 | 1116.92 | 0.84 | 0.00528991 | 0.266243368 |
| *LOC125180933* | 0.00 | 7.30 | -5.18 | 0.005297736 | 0.266243368 |
| *LOC106039201* | 346.64 | 592.85 | -0.77 | 0.005300478 | 0.266243368 |
| *CAPSL* | 250.98 | 85.94 | 1.55 | 0.005367825 | 0.266243368 |
| *PTPRS* | 115.29 | 49.81 | 1.22 | 0.005387072 | 0.266243368 |
| *CAMK2D* | 153.19 | 71.61 | 1.10 | 0.005406969 | 0.266243368 |
| *COL6A1* | 172.58 | 60.53 | 1.52 | 0.005413951 | 0.266243368 |
| *SLC22A31* | 14.96 | 1.13 | 3.74 | 0.00543174 | 0.266243368 |
| *ARHGAP45* | 229.97 | 109.37 | 1.07 | 0.005437483 | 0.266243368 |
| *BDH1* | 87.84 | 35.86 | 1.30 | 0.005439692 | 0.266243368 |
| *CREM* | 339.92 | 170.63 | 1.00 | 0.005453667 | 0.266243368 |
| *CDO1* | 2842.71 | 4270.63 | -0.59 | 0.00552467 | 0.26817457 |
| *PSMC3* | 296.27 | 501.40 | -0.76 | 0.005532184 | 0.26817457 |
| *POU2AF1* | 8.02 | 0.00 | 5.63 | 0.005560115 | 0.268582815 |
| *LOC125184403* | 330.81 | 193.83 | 0.77 | 0.005585998 | 0.268889357 |
| *LOC106048135* | 3.45 | 24.77 | -2.82 | 0.005605524 | 0.268889357 |
| *AEBP1* | 93.60 | 26.79 | 1.82 | 0.005650509 | 0.270106113 |
| *LOC125182833* | 1.91 | 18.62 | -3.36 | 0.005704339 | 0.271735774 |
| *IGFBP1* | 184.20 | 86.62 | 1.09 | 0.005790421 | 0.274885267 |
| *LOC106049209* | 55.79 | 14.67 | 1.95 | 0.0058383 | 0.276205783 |
| *PDP1* | 101.81 | 37.79 | 1.42 | 0.005859674 | 0.276267581 |
| *LOC106036374* | 796.92 | 1286.64 | -0.69 | 0.005957062 | 0.279900571 |
| *LOC106043531* | 64.23 | 26.53 | 1.27 | 0.006053259 | 0.282334543 |
| *RIMS4* | 46.43 | 12.97 | 1.83 | 0.006061099 | 0.282334543 |
| *LOC106049607* | 1305.56 | 699.60 | 0.90 | 0.006070387 | 0.282334543 |
| *LOC125184224* | 6.39 | 0.00 | 5.30 | 0.006167657 | 0.285750895 |
| *TM6SF2* | 145.36 | 269.89 | -0.89 | 0.006185354 | 0.285750895 |
| *NFAM1* | 19.67 | 3.86 | 2.34 | 0.006273108 | 0.288835706 |
| *COL17A1* | 6.43 | 0.00 | 5.31 | 0.006397892 | 0.293159673 |
| *CFD* | 532.39 | 235.53 | 1.18 | 0.006416185 | 0.293159673 |
| *ABHD17A* | 423.60 | 205.72 | 1.04 | 0.006430902 | 0.293159673 |
| *RAB8A* | 40.08 | 87.18 | -1.12 | 0.006458692 | 0.293335259 |
| *NMRK1* | 891.68 | 1593.71 | -0.84 | 0.006506604 | 0.293335259 |
| *HIC2* | 98.26 | 45.25 | 1.13 | 0.006520849 | 0.293335259 |
| *MRPS25* | 282.20 | 455.53 | -0.69 | 0.006539701 | 0.293335259 |
| *HSD3B1* | 544.29 | 268.03 | 1.02 | 0.006568481 | 0.293335259 |
| *LOC106041938* | 298.84 | 150.35 | 0.99 | 0.006571616 | 0.293335259 |
| *GPRIN1* | 7.65 | 0.00 | 5.58 | 0.006607903 | 0.293335259 |
| *LOC106032856* | 93.06 | 39.13 | 1.25 | 0.006622363 | 0.293335259 |
| *MAP1LC3C* | 6.58 | 0.00 | 5.36 | 0.006642289 | 0.293335259 |
| *MGAT3* | 49.66 | 11.63 | 2.10 | 0.006647825 | 0.293335259 |
| *ROS1* | 18.01 | 0.90 | 4.33 | 0.006710611 | 0.29515968 |
| *ATP1A1* | 283.26 | 132.83 | 1.10 | 0.006859444 | 0.29753459 |
| *ST3GAL3* | 280.33 | 147.44 | 0.93 | 0.006876645 | 0.29753459 |
| *RAB6A* | 447.50 | 691.94 | -0.63 | 0.00689571 | 0.29753459 |
| *AP1S3* | 518.43 | 962.19 | -0.89 | 0.006897545 | 0.29753459 |
| *LOC106039718* | 95.24 | 37.47 | 1.34 | 0.006899627 | 0.29753459 |
| *PHOSPHO1* | 51.84 | 109.86 | -1.08 | 0.006904513 | 0.29753459 |
| *SNED1* | 77.06 | 29.23 | 1.40 | 0.006915891 | 0.29753459 |
| *CLXN* | 7.66 | 0.00 | 5.57 | 0.006961855 | 0.297612495 |
| *ASMTL* | 143.56 | 271.78 | -0.92 | 0.006986744 | 0.297612495 |
| *HSF1* | 79.36 | 33.62 | 1.23 | 0.006995492 | 0.297612495 |
| *VAV1* | 103.27 | 40.22 | 1.36 | 0.007005898 | 0.297612495 |
| *CNN2* | 161.73 | 87.52 | 0.88 | 0.007073368 | 0.297612495 |
| *AIMP1* | 207.77 | 346.30 | -0.74 | 0.00707568 | 0.297612495 |
| *B4GALT2* | 158.49 | 64.98 | 1.29 | 0.007084062 | 0.297612495 |
| *LOC106044971* | 53.89 | 13.51 | 2.02 | 0.007090644 | 0.297612495 |
| *TRIM14* | 380.68 | 620.01 | -0.70 | 0.007164006 | 0.299013526 |
| *SLC6A6* | 270.87 | 139.10 | 0.96 | 0.007175084 | 0.299013526 |
| *LOC106048600* | 599.90 | 344.94 | 0.80 | 0.007189183 | 0.299013526 |
| *ARHGDIB* | 339.91 | 197.72 | 0.78 | 0.007278288 | 0.301807796 |
| *LOC106041932* | 46.08 | 10.50 | 2.15 | 0.007351045 | 0.303909435 |
| *GCLM* | 949.11 | 1723.01 | -0.86 | 0.007455402 | 0.307300971 |
| *NOTCH2* | 146.94 | 66.33 | 1.15 | 0.007560891 | 0.308744185 |
| *TNFRSF6B* | 125.15 | 48.71 | 1.36 | 0.007571583 | 0.308744185 |
| *LOC106029562* | 28.04 | 8.25 | 1.78 | 0.007579553 | 0.308744185 |
| *SUCLG1* | 576.86 | 882.08 | -0.61 | 0.007580122 | 0.308744185 |
| *LOC106048286* | 8.46 | 0.23 | 4.99 | 0.007645222 | 0.309992101 |
| *NT5C1B* | 89.70 | 35.40 | 1.35 | 0.007677356 | 0.309992101 |
| *LOC106040595* | 42.41 | 8.69 | 2.28 | 0.007678311 | 0.309992101 |
| *TOP3B* | 177.89 | 307.18 | -0.79 | 0.007822517 | 0.312415402 |
| *TTC39B* | 46.15 | 100.78 | -1.12 | 0.007834227 | 0.312415402 |
| *CUEDC1* | 197.97 | 91.97 | 1.11 | 0.007878937 | 0.312415402 |
| *GALNT11* | 82.13 | 38.47 | 1.10 | 0.007937116 | 0.312415402 |
| *LOC106037030* | 7873.49 | 12459.93 | -0.66 | 0.007937307 | 0.312415402 |
| *PLEC* | 85.67 | 33.77 | 1.34 | 0.00794371 | 0.312415402 |
| *SLC37A4* | 1158.59 | 742.25 | 0.64 | 0.007951669 | 0.312415402 |
| *LAMC1* | 254.88 | 136.90 | 0.90 | 0.007952214 | 0.312415402 |
| *SNAPC4* | 83.62 | 38.52 | 1.11 | 0.007979882 | 0.312415402 |
| *LOC125184504* | 162.86 | 304.54 | -0.90 | 0.007985548 | 0.312415402 |
| *LOC106036393* | 354.90 | 74.96 | 2.24 | 0.007987958 | 0.312415402 |
| *SRGN* | 98.07 | 39.46 | 1.32 | 0.008039841 | 0.313553781 |
| *LOC106040831* | 356.09 | 152.92 | 1.22 | 0.008093459 | 0.314009168 |
| *HPGD* | 1242.64 | 2102.71 | -0.76 | 0.008112667 | 0.314009168 |
| *CASZ1* | 27.79 | 6.17 | 2.19 | 0.008119944 | 0.314009168 |
| *CRIP1* | 208.15 | 125.14 | 0.74 | 0.008151255 | 0.314337051 |
| *MARS1* | 291.31 | 512.17 | -0.81 | 0.008292583 | 0.316558739 |
| *SWAP70* | 143.63 | 66.19 | 1.12 | 0.008295863 | 0.316558739 |
| *MBL* | 1119.27 | 2136.04 | -0.93 | 0.008318343 | 0.316558739 |
| *RPUSD1* | 588.15 | 322.97 | 0.86 | 0.008323125 | 0.316558739 |
| *PTPRE* | 15.66 | 1.52 | 3.35 | 0.008341324 | 0.316558739 |
| *DIPK1B* | 107.02 | 25.12 | 2.09 | 0.008359827 | 0.316558739 |
| *MYO1F* | 134.82 | 55.18 | 1.29 | 0.008392159 | 0.316558739 |
| *SLC25A13* | 330.56 | 535.18 | -0.70 | 0.008392819 | 0.316558739 |
| *ACAA2* | 2175.73 | 3763.00 | -0.79 | 0.00844216 | 0.317549767 |
| *HMGA2* | 45.89 | 10.32 | 2.16 | 0.00847041 | 0.317744249 |
| *CCDC17* | 1.02 | 11.70 | -3.51 | 0.00853992 | 0.318406211 |
| *IP6K2* | 362.82 | 601.24 | -0.73 | 0.008554528 | 0.318406211 |
| *PIK3IP1* | 46.67 | 15.37 | 1.60 | 0.008557442 | 0.318406211 |
| *MAVS* | 233.34 | 109.30 | 1.09 | 0.008604218 | 0.318806973 |
| *YPEL1* | 1358.86 | 814.29 | 0.74 | 0.008646031 | 0.318806973 |
| *LOC106041408* | 9.51 | 0.45 | 4.41 | 0.008704469 | 0.318806973 |
| *LOC106049927* | 44.76 | 10.95 | 2.04 | 0.008720338 | 0.318806973 |
| *TSPAN8* | 83.93 | 29.95 | 1.49 | 0.008751566 | 0.318806973 |
| *WDR54* | 334.08 | 141.68 | 1.24 | 0.008756112 | 0.318806973 |
| *LOC125182854* | 15.89 | 1.87 | 3.11 | 0.008807623 | 0.318806973 |
| *CROT* | 136.35 | 244.88 | -0.85 | 0.008811984 | 0.318806973 |
| *ILDR2* | 17.24 | 1.45 | 3.56 | 0.008846663 | 0.318806973 |
| *ARHGEF28* | 76.60 | 15.78 | 2.29 | 0.008868095 | 0.318806973 |
| *HCN3* | 30.28 | 4.99 | 2.61 | 0.008884312 | 0.318806973 |
| *RIPOR1* | 187.84 | 92.85 | 1.02 | 0.008893462 | 0.318806973 |
| *GNPDA2* | 133.76 | 234.92 | -0.82 | 0.008902748 | 0.318806973 |
| *NMD3* | 146.58 | 254.80 | -0.79 | 0.008907601 | 0.318806973 |
| *ANKFN1* | 1.22 | 15.11 | -3.69 | 0.008915572 | 0.318806973 |
| *TPD52* | 513.80 | 305.28 | 0.75 | 0.009008372 | 0.321290813 |
| *LOC106041242* | 2.09 | 17.58 | -3.12 | 0.009047377 | 0.321310767 |
| *ANKRD10* | 127.57 | 68.72 | 0.89 | 0.009058315 | 0.321310767 |
| *TMEM179B* | 9.16 | 35.69 | -1.93 | 0.009096497 | 0.321310767 |
| *MAP3K1* | 159.88 | 85.36 | 0.91 | 0.009102288 | 0.321310767 |
| *TOP1MT* | 53.05 | 149.51 | -1.49 | 0.009153585 | 0.322295153 |
| *LOC106045189* | 65.24 | 27.33 | 1.26 | 0.009278559 | 0.325862045 |
| *MGAT2* | 237.46 | 101.99 | 1.22 | 0.009393769 | 0.329068761 |
| *CEL* | 97.22 | 35.44 | 1.46 | 0.009594571 | 0.334922409 |
| *ERBB2* | 48.20 | 10.63 | 2.17 | 0.009609527 | 0.334922409 |
| *ETV4* | 8.06 | 0.19 | 4.93 | 0.009660887 | 0.335422638 |
| *EGF* | 7.32 | 32.01 | -2.14 | 0.009672607 | 0.335422638 |
| *LOC125183236* | 217.96 | 103.48 | 1.07 | 0.009901439 | 0.34197868 |
| *rna-NC_023832.1_4969..5041* | 653.40 | 380.65 | 0.78 | 0.009911345 | 0.34197868 |
| *HAUS3* | 22.89 | 4.76 | 2.25 | 0.009977267 | 0.343392577 |
| *LOC125182130* | 7.04 | 30.19 | -2.07 | 0.010022654 | 0.344094457 |
| *LOC106048144* | 31.93 | 10.44 | 1.63 | 0.010093111 | 0.345651402 |
| *LOC106030047* | 15.91 | 2.58 | 2.63 | 0.010125136 | 0.345887711 |
| *LOC125184406* | 242.06 | 124.40 | 0.96 | 0.010173457 | 0.34647922 |
| *DUSP1* | 129.83 | 58.87 | 1.14 | 0.010212333 | 0.34647922 |
| *LOC106033474* | 94.45 | 212.33 | -1.17 | 0.010217953 | 0.34647922 |
| *PSMC2* | 278.80 | 444.04 | -0.67 | 0.010263312 | 0.347162207 |
| *DUSP28* | 49.29 | 98.47 | -1.00 | 0.010358801 | 0.349533373 |
| *SORD* | 5532.08 | 8408.76 | -0.60 | 0.01041508 | 0.350573114 |
| *LOC106044243* | 8.91 | 0.00 | 5.78 | 0.010549678 | 0.352799202 |
| *GATA2* | 20.15 | 4.99 | 2.01 | 0.010553881 | 0.352799202 |
| *LOC106044181* | 63.82 | 25.72 | 1.30 | 0.010558093 | 0.352799202 |
| *PI4K2B* | 315.39 | 490.00 | -0.64 | 0.010602842 | 0.352805029 |
| *THBS4* | 6.88 | 0.00 | 5.41 | 0.010628192 | 0.352805029 |
| *EDC4* | 81.84 | 34.98 | 1.22 | 0.010644683 | 0.352805029 |
| *CAMK1D* | 176.50 | 90.61 | 0.96 | 0.010666827 | 0.352805029 |
| *CMTR1* | 143.69 | 64.25 | 1.16 | 0.010686402 | 0.352805029 |
| *DNMT3B* | 7.19 | 0.00 | 5.46 | 0.010731157 | 0.353435025 |
| *AGXT* | 333.15 | 547.63 | -0.72 | 0.010842203 | 0.355241 |
| *EIF2B5* | 110.39 | 199.59 | -0.86 | 0.010847644 | 0.355241 |
| *ALDOC* | 164.02 | 78.21 | 1.07 | 0.010863402 | 0.355241 |
| *SLC6A14* | 8.64 | 0.00 | 5.74 | 0.010901515 | 0.355642563 |
| *LOC106037882* | 7.28 | 0.00 | 5.49 | 0.010964808 | 0.356793106 |
| *FMNL1* | 83.61 | 40.58 | 1.05 | 0.011015555 | 0.356793106 |
| *UPB1* | 812.72 | 1236.72 | -0.61 | 0.011037423 | 0.356793106 |
| *LRRC3B* | 60.69 | 131.39 | -1.12 | 0.011040449 | 0.356793106 |
| *FZD9* | 688.41 | 343.83 | 1.00 | 0.011101491 | 0.357692162 |
| *CHMP2A* | 844.29 | 1231.63 | -0.55 | 0.011120233 | 0.357692162 |
| *PACS2* | 69.63 | 27.43 | 1.36 | 0.011183199 | 0.358600458 |
| *LOC106035060* | 10.67 | 0.38 | 4.65 | 0.011202246 | 0.358600458 |
| *ACE2* | 218.65 | 101.59 | 1.11 | 0.011226614 | 0.358600458 |
| *APELA* | 993.90 | 1665.62 | -0.75 | 0.011326884 | 0.360666112 |
| *CX3CR1* | 25.16 | 3.34 | 2.92 | 0.011343679 | 0.360666112 |
| *OSGIN1* | 982.78 | 1726.08 | -0.81 | 0.01139977 | 0.36161436 |
| *UAP1* | 249.59 | 139.86 | 0.84 | 0.011475296 | 0.362341346 |
| *PTGR3* | 266.69 | 453.64 | -0.77 | 0.011475327 | 0.362341346 |
| *CCL19* | 48.34 | 17.42 | 1.48 | 0.01154508 | 0.362987867 |
| *TLR7* | 7.67 | 0.23 | 4.85 | 0.011558032 | 0.362987867 |
| *MACF1* | 377.11 | 164.05 | 1.20 | 0.011574902 | 0.362987867 |
| *LOC106049663* | 747.72 | 494.75 | 0.60 | 0.011609629 | 0.363249456 |
| *SPATA13* | 195.12 | 324.87 | -0.73 | 0.01170214 | 0.363311196 |
| *PRPS2* | 10332.50 | 15574.44 | -0.59 | 0.011722152 | 0.363311196 |
| *LOC106047072* | 37.06 | 81.60 | -1.15 | 0.011737949 | 0.363311196 |
| *FAM234B* | 13.28 | 0.90 | 3.89 | 0.011760297 | 0.363311196 |
| *ATP1B1* | 120.67 | 60.33 | 1.00 | 0.01176863 | 0.363311196 |
| *FAM78A* | 60.59 | 18.69 | 1.71 | 0.011792503 | 0.363311196 |
| *RAD9A* | 79.84 | 36.36 | 1.13 | 0.011796332 | 0.363311196 |
| *LPCAT2* | 58.67 | 21.28 | 1.48 | 0.01182717 | 0.363447885 |
| *LOC125184519* | 0.67 | 11.53 | -4.30 | 0.011885438 | 0.363718015 |
| *LOC106049921* | 6.74 | 0.00 | 5.39 | 0.0118888 | 0.363718015 |
| *NAB2* | 14.48 | 1.89 | 2.92 | 0.012032431 | 0.367295958 |
| *SACM1L* | 291.07 | 477.99 | -0.72 | 0.012099045 | 0.36851227 |
| *PROC* | 1397.50 | 2071.26 | -0.57 | 0.012168989 | 0.369066779 |
| *LOC106044639* | 8.44 | 0.00 | 5.71 | 0.012198314 | 0.369066779 |
| *SLC39A13* | 57.43 | 22.09 | 1.39 | 0.012224191 | 0.369066779 |
| *BLMH* | 544.68 | 843.07 | -0.63 | 0.012224483 | 0.369066779 |
| *LOC106042084* | 3896.48 | 7059.27 | -0.86 | 0.012310609 | 0.36907713 |
| *GATA3* | 61.54 | 13.48 | 2.20 | 0.012328139 | 0.36907713 |
| *MIA2* | 202.18 | 110.83 | 0.87 | 0.012360412 | 0.36907713 |
| *ZNF710* | 121.02 | 67.45 | 0.85 | 0.012378497 | 0.36907713 |
| *DIMT1* | 119.07 | 207.11 | -0.80 | 0.012381604 | 0.36907713 |
| *LOC125180011* | 0.00 | 7.72 | -5.26 | 0.012431113 | 0.36907713 |
| *FPGS* | 887.29 | 1373.60 | -0.63 | 0.012435587 | 0.36907713 |
| *LOC125184893* | 0.84 | 17.48 | -4.28 | 0.012439296 | 0.36907713 |
| *TRAPPC4* | 141.79 | 250.82 | -0.83 | 0.012544051 | 0.371384833 |
| *CPN1* | 40.78 | 11.01 | 1.90 | 0.012622281 | 0.371869951 |
| *SYNC* | 13.49 | 1.24 | 3.42 | 0.012675667 | 0.371869951 |
| *NDUFA9* | 523.99 | 876.60 | -0.74 | 0.012685797 | 0.371869951 |
| *AMBRA1* | 139.03 | 76.39 | 0.86 | 0.012712754 | 0.371869951 |
| *LOC106046905* | 11310.01 | 6959.88 | 0.70 | 0.012730638 | 0.371869951 |
| *PLA1A* | 140.39 | 243.79 | -0.80 | 0.012730653 | 0.371869951 |
| *DACT1* | 78.10 | 28.86 | 1.44 | 0.01275072 | 0.371869951 |
| *ZNF821* | 94.37 | 46.90 | 1.01 | 0.012803084 | 0.371869951 |
| *LOC125181513* | 5.72 | 21.83 | -1.96 | 0.012824796 | 0.371869951 |
| *TNFRSF13B* | 8.53 | 0.00 | 5.72 | 0.012830553 | 0.371869951 |
| *AK1* | 57.19 | 131.15 | -1.19 | 0.012928622 | 0.373524972 |
| *KIAA0319L* | 132.05 | 60.59 | 1.12 | 0.01294192 | 0.373524972 |
| *ARFGEF2* | 216.50 | 372.74 | -0.79 | 0.012991335 | 0.374166765 |
| *CSF1R* | 96.04 | 47.43 | 1.03 | 0.013087609 | 0.37615264 |
| *POMT1* | 124.42 | 70.44 | 0.82 | 0.013144428 | 0.376998623 |
| *SERPIND1* | 3768.67 | 5696.54 | -0.60 | 0.013263167 | 0.379613346 |
| *TMEM150A* | 75.92 | 24.11 | 1.65 | 0.013322264 | 0.380146069 |
| *MPEG1* | 204.21 | 111.43 | 0.87 | 0.013337005 | 0.380146069 |
| *LOC125184412* | 185.97 | 80.71 | 1.20 | 0.01352948 | 0.384835432 |
| *RIPOR2* | 63.11 | 20.28 | 1.63 | 0.01360708 | 0.385639963 |
| *ECHS1* | 940.53 | 1440.30 | -0.62 | 0.013613788 | 0.385639963 |
| *PIM1* | 61.78 | 25.34 | 1.29 | 0.013731927 | 0.388109481 |
| *DIRAS3* | 0.00 | 9.45 | -5.56 | 0.013775623 | 0.388109481 |
| *ATP6V0D1* | 236.02 | 369.32 | -0.64 | 0.013785541 | 0.388109481 |
| *SLC2A13* | 79.99 | 31.71 | 1.33 | 0.013841741 | 0.388896438 |
| *DDRGK1* | 152.86 | 84.98 | 0.85 | 0.013912686 | 0.38968396 |
| *SLC23A1* | 298.00 | 136.62 | 1.13 | 0.013938339 | 0.38968396 |
| *LOC106042826* | 15.40 | 1.58 | 3.30 | 0.013954688 | 0.38968396 |
| *MAPK4* | 21.53 | 3.76 | 2.54 | 0.014166878 | 0.394790784 |
| *GRAMD2A* | 42.49 | 10.77 | 1.98 | 0.014194918 | 0.394790784 |
| *TMLHE* | 786.63 | 536.38 | 0.55 | 0.014333721 | 0.397469787 |
| *ND4L* | 73771.67 | 39790.85 | 0.89 | 0.014348986 | 0.397469787 |
| *FOSB* | 0.00 | 9.37 | -5.54 | 0.014392124 | 0.397612895 |
| *IMMT* | 252.98 | 403.67 | -0.67 | 0.014411915 | 0.397612895 |
| *DUSP5* | 68.67 | 29.95 | 1.20 | 0.014455969 | 0.398030661 |
| *LOC125180369* | 0.86 | 14.98 | -3.91 | 0.014507792 | 0.398134961 |
| *OAT* | 2052.39 | 2947.99 | -0.52 | 0.014517596 | 0.398134961 |
| *SLC39A1* | 207.82 | 83.50 | 1.31 | 0.014589425 | 0.39901944 |
| *NXPE3* | 14.00 | 1.72 | 3.09 | 0.014625455 | 0.39901944 |
| *GTF3C2* | 130.34 | 54.22 | 1.27 | 0.014636799 | 0.39901944 |
| *CCDC85C* | 75.14 | 132.65 | -0.83 | 0.014727894 | 0.399106681 |
| *LOC125180740* | 1560.53 | 2227.21 | -0.51 | 0.01474703 | 0.399106681 |
| *LOC125183309* | 7.90 | 0.00 | 5.62 | 0.014763985 | 0.399106681 |
| *CFAP53* | 33.52 | 9.16 | 1.89 | 0.014784709 | 0.399106681 |
| *DONSON* | 35.80 | 13.40 | 1.43 | 0.014807308 | 0.399106681 |
| *rna-NC_023832.1_11080..11150* | 295.33 | 140.49 | 1.07 | 0.01486093 | 0.399106681 |
| *EPHB3* | 36.67 | 10.95 | 1.74 | 0.014869701 | 0.399106681 |
| *LOC125181543* | 47.94 | 120.93 | -1.34 | 0.014884702 | 0.399106681 |
| *LOC106042682* | 282.69 | 463.22 | -0.71 | 0.01490091 | 0.399106681 |
| *RAB2A* | 804.13 | 1147.44 | -0.51 | 0.014944198 | 0.399488876 |
| *SCCPDH* | 505.60 | 807.00 | -0.67 | 0.015063984 | 0.401188814 |
| *BBS4* | 67.79 | 28.07 | 1.27 | 0.015066072 | 0.401188814 |
| *VPS28* | 1267.12 | 2222.95 | -0.81 | 0.015101547 | 0.401268651 |
| *LOC106037216* | 44.26 | 9.70 | 2.19 | 0.015127365 | 0.401268651 |
| *NUP205* | 83.35 | 156.33 | -0.91 | 0.015226855 | 0.403130993 |
| *APOF* | 1091.25 | 1976.97 | -0.86 | 0.015303915 | 0.403939795 |
| *LOC125180400* | 84.43 | 149.01 | -0.82 | 0.015316087 | 0.403939795 |
| *LGALS1* | 25.62 | 4.55 | 2.49 | 0.015367931 | 0.404532138 |
| *PPA2* | 284.82 | 425.87 | -0.58 | 0.015601769 | 0.409177565 |
| *ATP8* | 2652.83 | 1410.58 | 0.91 | 0.015603851 | 0.409177565 |
| *TSNARE1* | 18.05 | 4.04 | 2.17 | 0.015635516 | 0.409228414 |
| *TALDO1* | 351.70 | 547.75 | -0.64 | 0.015705998 | 0.410293108 |
| *BECN1* | 139.29 | 217.96 | -0.64 | 0.015798967 | 0.411940123 |
| *KIAA1522* | 264.84 | 155.21 | 0.77 | 0.015925667 | 0.413027907 |
| *NBEAL2* | 199.82 | 81.57 | 1.30 | 0.015948322 | 0.413027907 |
| *LOC125180944* | 12.22 | 1.13 | 3.46 | 0.015952066 | 0.413027907 |
| *NKX6-3* | 46.65 | 15.15 | 1.63 | 0.015960692 | 0.413027907 |
| *PMEPA1* | 295.97 | 150.37 | 0.98 | 0.016014543 | 0.413526721 |
| *TF* | 114369.36 | 165142.51 | -0.53 | 0.016055425 | 0.413526721 |
| *LOC125182356* | 0.34 | 8.24 | -4.64 | 0.016133527 | 0.413526721 |
| *YARS1* | 175.23 | 336.53 | -0.94 | 0.016159372 | 0.413526721 |
| *ATXN3* | 117.41 | 197.46 | -0.75 | 0.016165813 | 0.413526721 |
| *GFRA3* | 12.60 | 45.72 | -1.84 | 0.016173649 | 0.413526721 |
| *BTK* | 113.34 | 54.34 | 1.06 | 0.016197973 | 0.413526721 |
| *TAMM41* | 32.95 | 71.81 | -1.12 | 0.016220268 | 0.413526721 |
| *ZNF106* | 71.48 | 36.29 | 0.98 | 0.016295596 | 0.414679253 |
| *TAX1BP1* | 371.48 | 588.72 | -0.67 | 0.016437486 | 0.41650222 |
| *TAF5L* | 39.70 | 78.09 | -0.98 | 0.016488719 | 0.41650222 |
| *WHRN* | 7.19 | 0.23 | 4.75 | 0.01649718 | 0.41650222 |
| *LOC125179930* | 31.81 | 4.72 | 2.75 | 0.016505354 | 0.41650222 |
| *FOXL3* | 77.08 | 21.15 | 1.87 | 0.016557454 | 0.41650222 |
| *AMN* | 410.23 | 717.16 | -0.80 | 0.016563375 | 0.41650222 |
| *TTPA* | 3850.47 | 5953.95 | -0.63 | 0.016688619 | 0.41650222 |
| *VPS26B* | 103.08 | 53.09 | 0.96 | 0.016715979 | 0.41650222 |
| *TCTN2* | 32.11 | 8.71 | 1.89 | 0.016716098 | 0.41650222 |
| *GIGYF2* | 249.41 | 154.27 | 0.69 | 0.016748601 | 0.41650222 |
| *FANCG* | 89.18 | 40.54 | 1.14 | 0.016774666 | 0.41650222 |
| *PIK3R5* | 48.14 | 16.67 | 1.54 | 0.016802305 | 0.41650222 |
| *ZMAT2* | 206.94 | 340.59 | -0.72 | 0.016828571 | 0.41650222 |
| *STARD10* | 81.72 | 33.54 | 1.28 | 0.01683493 | 0.41650222 |
| *CLDN10* | 21.33 | 4.10 | 2.41 | 0.016844463 | 0.41650222 |
| *TYRO3* | 53.74 | 16.50 | 1.72 | 0.016961718 | 0.41650222 |
| *TMEM116* | 7.89 | 0.45 | 4.14 | 0.016980525 | 0.41650222 |
| *AFF2* | 105.02 | 41.95 | 1.33 | 0.016984036 | 0.41650222 |
| *CRYL1* | 1309.65 | 2059.54 | -0.65 | 0.01699704 | 0.41650222 |
| *CLIC6* | 96.27 | 27.01 | 1.84 | 0.016997517 | 0.41650222 |
| *AMDHD1* | 679.88 | 1042.91 | -0.62 | 0.01700256 | 0.41650222 |
| *LOC125182825* | 0.00 | 9.11 | -5.50 | 0.01706334 | 0.417248672 |
| *LOC106041184* | 101.72 | 181.89 | -0.84 | 0.017143703 | 0.417912627 |
| *LOC106041042* | 7.76 | 0.00 | 5.58 | 0.017189226 | 0.417912627 |
| *MND1* | 7.07 | 24.91 | -1.77 | 0.017210748 | 0.417912627 |
| *TLE1* | 72.18 | 34.75 | 1.05 | 0.017238999 | 0.417912627 |
| *LOC125183316* | 14.74 | 41.32 | -1.46 | 0.017242273 | 0.417912627 |
| *TMEM127* | 45.45 | 15.88 | 1.52 | 0.01732983 | 0.418706694 |
| *LOC106033756* | 1442.50 | 2405.44 | -0.74 | 0.017359113 | 0.418706694 |
| *MZB1* | 29.39 | 4.21 | 2.81 | 0.017393514 | 0.418706694 |
| *COL12A1* | 46.54 | 17.12 | 1.44 | 0.01739669 | 0.418706694 |
| *CSNK1G2* | 280.45 | 154.32 | 0.86 | 0.017471152 | 0.419764998 |
| *LOC106044017* | 11.13 | 1.16 | 3.30 | 0.017561126 | 0.421191673 |
| *TNKS1BP1* | 49.78 | 16.33 | 1.62 | 0.017635019 | 0.422228366 |
| *LOC106043403* | 63.10 | 17.06 | 1.89 | 0.017712379 | 0.422580548 |
| *BNIP1* | 210.06 | 336.65 | -0.68 | 0.017740896 | 0.422580548 |
| *TCP1* | 385.27 | 563.25 | -0.55 | 0.017777345 | 0.422580548 |
| *SLC1A1* | 24.99 | 7.34 | 1.76 | 0.017795247 | 0.422580548 |
| *CDKN1A* | 420.34 | 216.62 | 0.95 | 0.017816275 | 0.422580548 |
| *ACADM* | 1563.32 | 2607.13 | -0.74 | 0.01786247 | 0.422580548 |
| *ERMP1* | 75.64 | 140.59 | -0.89 | 0.017919313 | 0.422580548 |
| *VNN1* | 1027.62 | 1740.81 | -0.76 | 0.017959088 | 0.422580548 |
| *DEAF1* | 23.92 | 6.75 | 1.83 | 0.017961795 | 0.422580548 |
| *IPO11* | 61.86 | 109.83 | -0.82 | 0.01798349 | 0.422580548 |
| *NDUFS3* | 703.13 | 1052.22 | -0.58 | 0.017995853 | 0.422580548 |
| *MINDY4* | 24.02 | 5.47 | 2.13 | 0.018018071 | 0.422580548 |
| *DHRS3* | 185.86 | 300.47 | -0.69 | 0.018312573 | 0.427599513 |
| *GINS2* | 96.37 | 48.09 | 1.00 | 0.018321581 | 0.427599513 |
| *SPEN* | 124.42 | 61.67 | 1.01 | 0.018329888 | 0.427599513 |
| *IL20RA* | 14.25 | 1.16 | 3.57 | 0.018356309 | 0.427599513 |
| *ALDH3A2* | 730.69 | 1085.38 | -0.57 | 0.018604101 | 0.431948845 |
| *DHDDS* | 146.84 | 226.51 | -0.63 | 0.018605772 | 0.431948845 |
| *VWA8* | 448.31 | 669.36 | -0.58 | 0.018692883 | 0.43273415 |
| *KLHL5* | 94.07 | 165.75 | -0.81 | 0.018705944 | 0.43273415 |
| *ADAMTS1* | 28.93 | 6.92 | 2.07 | 0.018733897 | 0.43273415 |
| *ATL2* | 129.01 | 212.86 | -0.72 | 0.018826842 | 0.433918586 |
| *CTNNAL1* | 117.71 | 49.62 | 1.24 | 0.018884497 | 0.433918586 |
| *MLX* | 168.32 | 95.93 | 0.81 | 0.018903025 | 0.433918586 |
| *ANXA13* | 54.38 | 20.85 | 1.39 | 0.018911248 | 0.433918586 |
| *HSPH1* | 69.30 | 132.50 | -0.93 | 0.018973572 | 0.434624228 |
| *PMFBP1* | 5.75 | 0.23 | 4.43 | 0.019022461 | 0.43483575 |
| *LOC125179926* | 0.30 | 8.53 | -4.69 | 0.019045976 | 0.43483575 |
| *ZNF593* | 132.05 | 221.22 | -0.74 | 0.019237533 | 0.437602693 |
| *PTCD3* | 66.92 | 116.95 | -0.81 | 0.019253803 | 0.437602693 |
| *STEAP2* | 3.95 | 22.46 | -2.49 | 0.019262529 | 0.437602693 |
| *SPI1* | 335.77 | 202.70 | 0.73 | 0.019361904 | 0.439135642 |
| *LOC125183647* | 276.78 | 488.59 | -0.82 | 0.019467756 | 0.440810185 |
| *LOC106048412* | 20.03 | 4.09 | 2.31 | 0.019519616 | 0.44092205 |
| *LOC106048849* | 32.39 | 6.43 | 2.32 | 0.019539707 | 0.44092205 |
| *LOC106049428* | 372.20 | 99.68 | 1.90 | 0.019600078 | 0.44092205 |
| *LOC106029917* | 82.16 | 34.39 | 1.25 | 0.019600806 | 0.44092205 |
| *SHMT2* | 2201.67 | 3474.01 | -0.66 | 0.019661719 | 0.440929592 |
| *GOLPH3L* | 67.92 | 17.01 | 1.99 | 0.019679814 | 0.440929592 |
| *ARVCF* | 84.12 | 38.98 | 1.11 | 0.019697225 | 0.440929592 |
| *CCP110* | 23.95 | 5.23 | 2.17 | 0.019851108 | 0.443385886 |
| *PXN* | 176.53 | 104.77 | 0.75 | 0.019871366 | 0.443385886 |
| *RAC2* | 169.90 | 105.91 | 0.68 | 0.01998969 | 0.445304318 |
| *KPNA6* | 91.59 | 170.89 | -0.89 | 0.020057194 | 0.446086248 |
| *PIGT* | 153.27 | 72.16 | 1.09 | 0.020144119 | 0.446881457 |
| *MMUT* | 317.89 | 500.17 | -0.66 | 0.020259981 | 0.446881457 |
| *FOSL2* | 64.96 | 21.47 | 1.61 | 0.020274188 | 0.446881457 |
| *ZNF618* | 88.58 | 41.13 | 1.11 | 0.02030486 | 0.446881457 |
| *ABHD18* | 89.76 | 163.59 | -0.87 | 0.020307095 | 0.446881457 |
| *MRPS35* | 286.64 | 454.77 | -0.67 | 0.020307109 | 0.446881457 |
| *ZDHHC13* | 45.25 | 13.79 | 1.71 | 0.020343819 | 0.446881457 |
| *TMEM229B* | 68.15 | 29.80 | 1.19 | 0.020414297 | 0.446881457 |
| *FOXP4* | 55.11 | 20.03 | 1.45 | 0.020450653 | 0.446881457 |
| *LOC106048707* | 22.34 | 4.81 | 2.22 | 0.020498238 | 0.446881457 |
| *SHE* | 307.88 | 178.84 | 0.79 | 0.020505008 | 0.446881457 |
| *DDX28* | 47.92 | 95.31 | -1.00 | 0.020510069 | 0.446881457 |
| *SRSF1* | 1030.95 | 710.44 | 0.54 | 0.020514933 | 0.446881457 |
| *SREBF2* | 168.59 | 92.56 | 0.86 | 0.020649507 | 0.449102304 |
| *BAIAP2L1* | 64.10 | 117.59 | -0.87 | 0.02075415 | 0.449771837 |
| *PTP4A3* | 368.91 | 223.08 | 0.73 | 0.020777964 | 0.449771837 |
| *LOC106049871* | 7.44 | 30.90 | -2.04 | 0.020778302 | 0.449771837 |
| *MKLN1* | 258.84 | 393.55 | -0.61 | 0.020986241 | 0.453559783 |
| *JAML* | 28.09 | 8.34 | 1.75 | 0.021061084 | 0.45445193 |
| *ACSL4* | 102.18 | 54.36 | 0.92 | 0.021184181 | 0.45445193 |
| *MAN1C1* | 30.05 | 11.04 | 1.45 | 0.021211306 | 0.45445193 |
| *PRICKLE1* | 22.69 | 5.63 | 2.02 | 0.021229045 | 0.45445193 |
| *LCORL* | 70.83 | 32.94 | 1.10 | 0.021231953 | 0.45445193 |
| *PIP4P2* | 360.23 | 558.61 | -0.63 | 0.021246884 | 0.45445193 |
| *HIVEP2* | 72.43 | 34.45 | 1.08 | 0.021258593 | 0.45445193 |
| *PFKL* | 840.65 | 1351.00 | -0.69 | 0.021392206 | 0.455417531 |
| *MRPS30* | 469.31 | 754.70 | -0.69 | 0.021406568 | 0.455417531 |
| *CACNA1G* | 17.00 | 1.77 | 3.29 | 0.021413139 | 0.455417531 |
| *DDX18* | 272.92 | 402.58 | -0.56 | 0.021448753 | 0.455417531 |
| *SMG1* | 88.15 | 48.01 | 0.88 | 0.021469164 | 0.455417531 |
| *NR2F6* | 977.15 | 535.39 | 0.87 | 0.021532079 | 0.456049431 |
| *LOC106033295* | 136.48 | 74.64 | 0.86 | 0.021625322 | 0.457320744 |
| *LOC106048013* | 32.13 | 0.90 | 5.16 | 0.021709911 | 0.458405433 |
| *LOC125183365* | 4.33 | 19.74 | -2.18 | 0.021780629 | 0.459194371 |
| *EPCAM* | 33.77 | 8.74 | 1.95 | 0.021816627 | 0.459249996 |
| *KCNAB1* | 9.16 | 0.45 | 4.35 | 0.021862909 | 0.459521636 |
| *LOC106042369* | 26.92 | 55.21 | -1.04 | 0.021940341 | 0.459763687 |
| *LYPD6* | 5.94 | 0.00 | 5.21 | 0.021941218 | 0.459763687 |
| *TRMT1L* | 131.90 | 239.85 | -0.86 | 0.022033507 | 0.460620826 |
| *OSBPL5* | 45.34 | 17.15 | 1.41 | 0.022049039 | 0.460620826 |
| *LHFPL7* | 89.30 | 172.76 | -0.95 | 0.022097026 | 0.460923877 |
| *LOC106048101* | 99.28 | 53.43 | 0.89 | 0.022136338 | 0.461045336 |
| *SECISBP2L* | 182.05 | 301.87 | -0.73 | 0.022228095 | 0.461262009 |
| *LOC125184116* | 4.23 | 20.16 | -2.25 | 0.022240849 | 0.461262009 |
| *CYSTM1* | 300.96 | 176.25 | 0.77 | 0.022272061 | 0.461262009 |
| *ELMOD3* | 51.77 | 17.87 | 1.53 | 0.022280761 | 0.461262009 |
| *FBXL2* | 14.47 | 2.06 | 2.81 | 0.022449492 | 0.464057294 |
| *LOC106039669* | 133.24 | 274.67 | -1.03 | 0.022520983 | 0.464837139 |
| *FASTK* | 267.70 | 151.33 | 0.82 | 0.022607566 | 0.465925688 |
| *LOC106044997* | 6.75 | 25.09 | -1.88 | 0.0226828 | 0.466434175 |
| *TMEM242* | 214.25 | 327.77 | -0.61 | 0.022713098 | 0.466434175 |
| *NUDT19* | 100.05 | 173.30 | -0.79 | 0.022757493 | 0.466434175 |
| *NTM* | 5.73 | 0.00 | 5.15 | 0.022767761 | 0.466434175 |
| *UFL1* | 135.58 | 211.65 | -0.64 | 0.022881606 | 0.467668835 |
| *COL1A2* | 255.17 | 123.97 | 1.05 | 0.02291368 | 0.467668835 |
| *LOC106033442* | 6.56 | 0.23 | 4.63 | 0.022929939 | 0.467668835 |
| *OCEL1* | 35.51 | 6.80 | 2.42 | 0.022966869 | 0.467729112 |
| *ZFYVE28* | 8.56 | 0.62 | 3.75 | 0.023059503 | 0.468921969 |
| *ACER2* | 29.92 | 10.50 | 1.50 | 0.023100835 | 0.469069615 |
| *RBM12* | 85.87 | 148.49 | -0.79 | 0.023164537 | 0.469670366 |
| *YPEL2* | 158.35 | 86.70 | 0.86 | 0.023304666 | 0.471816662 |
| *RIOX2* | 52.07 | 102.77 | -0.99 | 0.023340095 | 0.471833404 |
| *LOC106034024* | 2026.49 | 3113.09 | -0.62 | 0.023374038 | 0.471833404 |
| *SEPSECS* | 201.93 | 313.43 | -0.64 | 0.023474361 | 0.473164749 |
| *HMGB3* | 700.05 | 995.75 | -0.51 | 0.023547106 | 0.473937152 |
| *CRIM1* | 36.67 | 11.40 | 1.69 | 0.023584786 | 0.474002551 |
| *LOC106029742* | 18.49 | 64.78 | -1.80 | 0.023678944 | 0.47499233 |
| *LOC106040319* | 5.53 | 0.00 | 5.10 | 0.023703038 | 0.47499233 |
| *RUVBL1* | 84.67 | 140.70 | -0.73 | 0.023738246 | 0.47500644 |
| *LOC106036455* | 5.54 | 0.00 | 5.11 | 0.023845101 | 0.476452105 |
| *ACP1* | 722.86 | 1092.29 | -0.59 | 0.023987892 | 0.477856802 |
| *MRPL16* | 818.60 | 458.29 | 0.84 | 0.024019467 | 0.477856802 |
| *LOC106038213* | 3.01 | 16.04 | -2.43 | 0.024050948 | 0.477856802 |
| *FBLN5* | 11.42 | 0.68 | 4.10 | 0.024054243 | 0.477856802 |
| *SELENOP* | 5195.21 | 7509.85 | -0.53 | 0.024115478 | 0.477907035 |
| *FBXW8* | 223.72 | 144.43 | 0.63 | 0.0241262 | 0.477907035 |
| *LOC106041672* | 574.91 | 373.10 | 0.62 | 0.024380942 | 0.481771083 |
| *GLOD4* | 279.32 | 420.24 | -0.59 | 0.024391258 | 0.481771083 |
| *LOC125181581* | 23.85 | 6.13 | 1.95 | 0.024594208 | 0.485083756 |
| *TIAM1* | 64.00 | 26.45 | 1.27 | 0.024663283 | 0.485572965 |
| *DHCR24* | 376.61 | 214.12 | 0.81 | 0.024689553 | 0.485572965 |
| *DAAM1* | 71.66 | 131.10 | -0.87 | 0.024807762 | 0.485606606 |
| *NDUFV1* | 1815.91 | 2650.65 | -0.55 | 0.024817498 | 0.485606606 |
| *MYLK* | 610.72 | 344.68 | 0.83 | 0.024903627 | 0.485606606 |
| *SCARF1* | 7.40 | 23.27 | -1.66 | 0.02494096 | 0.485606606 |
| *LOC106045670* | 30.90 | 7.50 | 2.02 | 0.024967625 | 0.485606606 |
| *SH3BP5* | 844.18 | 1208.33 | -0.52 | 0.024988791 | 0.485606606 |
| *EIF2S1* | 198.99 | 308.49 | -0.64 | 0.025008719 | 0.485606606 |
| *FAM126B* | 489.38 | 759.54 | -0.64 | 0.025018359 | 0.485606606 |
| *PSMD9* | 331.80 | 510.04 | -0.62 | 0.025033855 | 0.485606606 |
| *ABCA2* | 200.91 | 84.15 | 1.26 | 0.025043996 | 0.485606606 |
| *MTR* | 51.74 | 111.02 | -1.10 | 0.025137224 | 0.486316249 |
| *TTLL13* | 148.52 | 58.29 | 1.36 | 0.025204458 | 0.486316249 |
| *TRADD* | 16.29 | 2.60 | 2.64 | 0.025218548 | 0.486316249 |
| *LOC106047160* | 47.86 | 12.11 | 1.98 | 0.025221893 | 0.486316249 |
| *METTL26* | 143.42 | 234.88 | -0.71 | 0.02527315 | 0.486623006 |
| *TLDC2* | 6.68 | 0.00 | 5.38 | 0.025458871 | 0.488034813 |
| *RPS29* | 958.49 | 1696.81 | -0.82 | 0.025479375 | 0.488034813 |
| *SLC27A1* | 43.54 | 12.56 | 1.78 | 0.025505965 | 0.488034813 |
| *LOC106035885* | 54.70 | 22.93 | 1.25 | 0.025525972 | 0.488034813 |
| *LOC106029538* | 3022.66 | 4629.78 | -0.62 | 0.0255904 | 0.488034813 |
| *ASPA* | 38.53 | 82.17 | -1.10 | 0.025603675 | 0.488034813 |
| *LOC106031078* | 43.71 | 19.78 | 1.15 | 0.02561967 | 0.488034813 |
| *NANOS1* | 23.77 | 5.97 | 2.01 | 0.02563007 | 0.488034813 |
| *ANXA4* | 791.94 | 469.90 | 0.75 | 0.02570157 | 0.488720324 |
| *TDO2* | 2115.22 | 3172.12 | -0.58 | 0.025768409 | 0.488746063 |
| *CSTF1* | 82.33 | 135.35 | -0.71 | 0.025773926 | 0.488746063 |
| *SH3BGRL* | 719.26 | 471.93 | 0.61 | 0.025865741 | 0.489812458 |
| *PAAF1* | 47.21 | 98.82 | -1.06 | 0.025908142 | 0.48994148 |
| *SNAP25* | 9.14 | 0.52 | 4.32 | 0.026019372 | 0.491053593 |
| *EPB41L1* | 140.98 | 81.94 | 0.78 | 0.026109868 | 0.491053593 |
| *LOC106035746* | 47.07 | 17.05 | 1.45 | 0.026145485 | 0.491053593 |
| *BCL9L* | 78.49 | 39.32 | 1.00 | 0.026174761 | 0.491053593 |
| *CUNH4orf19* | 5.40 | 0.00 | 5.07 | 0.026178795 | 0.491053593 |
| *SLC16A5* | 127.52 | 218.51 | -0.77 | 0.02619903 | 0.491053593 |
| *NID1* | 94.66 | 44.43 | 1.09 | 0.026216633 | 0.491053593 |
| *VAV2* | 416.11 | 233.87 | 0.83 | 0.026263473 | 0.491262542 |
| *LOC106049553* | 203.04 | 132.80 | 0.61 | 0.026334477 | 0.49192232 |
| *APOH* | 13585.60 | 19902.52 | -0.55 | 0.026450863 | 0.493426864 |
| *CD82* | 665.92 | 459.60 | 0.53 | 0.026596678 | 0.495364838 |
| *IREB2* | 288.98 | 420.78 | -0.54 | 0.026662848 | 0.495364838 |
| *LOC106045192* | 6.85 | 0.00 | 5.42 | 0.026676669 | 0.495364838 |
| *PDRG1* | 56.74 | 102.44 | -0.86 | 0.026733288 | 0.495364838 |
| *NRXN1* | 202.93 | 120.17 | 0.76 | 0.026734661 | 0.495364838 |
| *SLC4A4* | 134.42 | 73.90 | 0.87 | 0.026834912 | 0.495680562 |
| *RIOK3* | 799.13 | 1250.19 | -0.65 | 0.026847432 | 0.495680562 |
| *LOC106047992* | 71.74 | 35.14 | 1.04 | 0.026860665 | 0.495680562 |
| *FIG4* | 56.10 | 26.50 | 1.08 | 0.026908014 | 0.495680562 |
| *LOC106037939* | 14078.93 | 22475.47 | -0.67 | 0.026931725 | 0.495680562 |
| *PGAP6* | 40.87 | 12.01 | 1.76 | 0.027021338 | 0.496476651 |
| *P3H3* | 100.91 | 43.34 | 1.22 | 0.027047105 | 0.496476651 |
| *ZNHIT3* | 63.14 | 141.52 | -1.16 | 0.027199319 | 0.497425772 |
| *EVI2B* | 19.20 | 5.12 | 1.93 | 0.027206437 | 0.497425772 |
| *NEU3* | 75.84 | 31.43 | 1.27 | 0.027240175 | 0.497425772 |
| *JMJD7* | 30.29 | 8.93 | 1.76 | 0.027243338 | 0.497425772 |
| *TGM4* | 29.24 | 4.17 | 2.81 | 0.027333066 | 0.498368515 |
| *LOC106043421* | 5.58 | 0.00 | 5.10 | 0.027371517 | 0.498368515 |
| *PISD* | 39.88 | 88.14 | -1.15 | 0.027448287 | 0.498368515 |
| *LOC106044594* | 810.23 | 1220.53 | -0.59 | 0.027450622 | 0.498368515 |
| *NPR2* | 26.42 | 5.12 | 2.39 | 0.027475972 | 0.498368515 |
| *BBS12* | 68.71 | 25.48 | 1.43 | 0.027541008 | 0.498890864 |
| *PDK3* | 365.78 | 210.24 | 0.80 | 0.027587795 | 0.499060313 |
| *RGS16* | 5.34 | 0.00 | 5.05 | 0.027622863 | 0.499060313 |
| *ASNS* | 56.21 | 125.92 | -1.16 | 0.02776262 | 0.500927901 |
| *UNK* | 103.44 | 56.45 | 0.88 | 0.027923134 | 0.50296586 |
| *CELSR3* | 15.98 | 2.97 | 2.45 | 0.027951532 | 0.50296586 |
| *FAM219B* | 18.26 | 3.17 | 2.53 | 0.027985171 | 0.50296586 |
| *GLO1* | 583.07 | 854.72 | -0.55 | 0.028081679 | 0.50403444 |
| *LAMA3* | 27.27 | 8.83 | 1.64 | 0.02815229 | 0.50403444 |
| *ADAMTS8* | 43.63 | 12.68 | 1.78 | 0.028154462 | 0.50403444 |
| *LOC106045475* | 436.30 | 672.41 | -0.62 | 0.028217192 | 0.504501403 |
| *LOC125183335* | 64.27 | 23.60 | 1.45 | 0.028332205 | 0.505900734 |
| *RTN4* | 1049.48 | 1516.20 | -0.53 | 0.028375968 | 0.506025851 |
| *DUS4L* | 181.46 | 268.73 | -0.57 | 0.028475826 | 0.50628376 |
| *FOXP1* | 316.40 | 186.65 | 0.77 | 0.028492869 | 0.50628376 |
| *AP4B1* | 63.17 | 32.12 | 0.98 | 0.028500756 | 0.50628376 |
| *DCLK1* | 0.00 | 4.82 | -4.58 | 0.028578441 | 0.507009541 |
| *TRMT10C* | 294.86 | 445.84 | -0.59 | 0.02869299 | 0.508267893 |
| *FTSJ3* | 226.37 | 363.51 | -0.68 | 0.028723209 | 0.508267893 |
| *TRIM3* | 229.80 | 118.63 | 0.96 | 0.028795793 | 0.508389619 |
| *ATF4* | 3106.24 | 4568.15 | -0.56 | 0.028835513 | 0.508389619 |
| *ABAT* | 614.05 | 383.81 | 0.68 | 0.028840873 | 0.508389619 |
| *EIF1* | 927.51 | 1467.12 | -0.66 | 0.02897246 | 0.510056081 |
| *TSPAN1* | 54.65 | 15.74 | 1.79 | 0.029063445 | 0.51100254 |
| *MEF2D* | 41.57 | 14.65 | 1.50 | 0.029100457 | 0.51100254 |
| *G6PC1* | 2616.24 | 3945.06 | -0.59 | 0.029206947 | 0.512075535 |
| *ABCD3* | 259.63 | 381.09 | -0.55 | 0.02926553 | 0.512075535 |
| *LOC106049872* | 10.11 | 0.45 | 4.47 | 0.029273149 | 0.512075535 |
| *DPYSL3* | 69.43 | 34.99 | 0.98 | 0.029374073 | 0.512908686 |
| *LOC125184249* | 191.30 | 87.40 | 1.14 | 0.02939529 | 0.512908686 |
| *KCNG3* | 0.00 | 5.87 | -4.87 | 0.029481728 | 0.513640187 |
| *PIERCE1* | 26.49 | 58.53 | -1.15 | 0.029511832 | 0.513640187 |
| *ASB6* | 107.69 | 60.66 | 0.83 | 0.0296301 | 0.513655462 |
| *DDX47* | 130.00 | 210.59 | -0.70 | 0.029644149 | 0.513655462 |
| *GPSM2* | 86.09 | 149.97 | -0.79 | 0.029646452 | 0.513655462 |
| *PPFIBP2* | 137.72 | 244.56 | -0.83 | 0.029661952 | 0.513655462 |
| *CCT7* | 408.90 | 615.02 | -0.59 | 0.029757509 | 0.514662847 |
| *FABP5* | 159.41 | 299.22 | -0.91 | 0.029843963 | 0.515510469 |
| *SCP2* | 7237.57 | 10348.05 | -0.52 | 0.029948421 | 0.516666564 |
| *TAPT1* | 92.40 | 162.29 | -0.82 | 0.030007025 | 0.517029671 |
| *SRC* | 184.96 | 111.77 | 0.72 | 0.030049276 | 0.517110477 |
| *LOC106045955* | 16.19 | 1.91 | 3.04 | 0.030151511 | 0.517534783 |
| *NAE1* | 41.23 | 80.37 | -0.97 | 0.030153292 | 0.517534783 |
| *ARHGAP9* | 41.31 | 14.41 | 1.53 | 0.030207895 | 0.517534783 |
| *CUNH6orf132* | 68.10 | 120.73 | -0.83 | 0.030224302 | 0.517534783 |
| *LOC106046849* | 11.59 | 34.38 | -1.55 | 0.030365858 | 0.517599382 |
| *SNAP91* | 30.72 | 8.00 | 1.96 | 0.030376023 | 0.517599382 |
| *RRP9* | 68.22 | 144.87 | -1.08 | 0.030401399 | 0.517599382 |
| *DNAJC28* | 173.27 | 292.66 | -0.76 | 0.03052605 | 0.517599382 |
| *TIMM9* | 90.56 | 166.93 | -0.88 | 0.030587019 | 0.517599382 |
| *LOC125183005* | 157.72 | 309.79 | -0.97 | 0.030623682 | 0.517599382 |
| *TMEM260* | 89.51 | 47.24 | 0.92 | 0.030634975 | 0.517599382 |
| *LOC106029497* | 20.29 | 4.77 | 2.10 | 0.030643605 | 0.517599382 |
| *EVI5L* | 502.92 | 323.97 | 0.63 | 0.030705389 | 0.517599382 |
| *COBLL1* | 379.80 | 561.25 | -0.57 | 0.030717243 | 0.517599382 |
| *BCL9* | 68.12 | 33.55 | 1.02 | 0.030749515 | 0.517599382 |
| *FAHD1* | 95.09 | 171.28 | -0.85 | 0.030749941 | 0.517599382 |
| *LOC125181024* | 0.00 | 7.33 | -5.18 | 0.030782666 | 0.517599382 |
| *B3GNT7* | 74.51 | 25.19 | 1.57 | 0.030818629 | 0.517599382 |
| *ZFPM1* | 97.37 | 47.56 | 1.03 | 0.03082551 | 0.517599382 |
| *RAN* | 839.97 | 1200.66 | -0.52 | 0.030883614 | 0.517599382 |
| *HMGCR* | 110.00 | 53.08 | 1.05 | 0.030928798 | 0.517599382 |
| *ATP6V1H* | 361.03 | 549.65 | -0.61 | 0.030934958 | 0.517599382 |
| *TRIM71* | 6.28 | 0.00 | 5.29 | 0.03094242 | 0.517599382 |
| *WWTR1* | 138.90 | 228.24 | -0.71 | 0.030995666 | 0.517860838 |
| *NSUN7* | 6.35 | 0.00 | 5.31 | 0.031068216 | 0.5184438 |
| *LOC125180033* | 3.62 | 15.97 | -2.13 | 0.031221619 | 0.519835929 |
| *ST3GAL1* | 133.57 | 66.37 | 1.02 | 0.031259468 | 0.519835929 |
| *GLRX2* | 284.12 | 406.37 | -0.52 | 0.03126492 | 0.519835929 |
| *TMEM104* | 54.67 | 25.01 | 1.13 | 0.03131165 | 0.51998491 |
| *IGF2BP2* | 566.60 | 333.28 | 0.77 | 0.031397871 | 0.52078854 |
| *LOC125182987* | 0.96 | 9.67 | -3.43 | 0.031440696 | 0.520871317 |
| *LOC125184687* | 0.00 | 5.82 | -4.85 | 0.031514576 | 0.521230621 |
| *SERPINE2* | 54.68 | 20.54 | 1.42 | 0.031564646 | 0.521230621 |
| *SLC50A1* | 32.42 | 9.63 | 1.75 | 0.031580641 | 0.521230621 |
| *MRPS18C* | 9.33 | 26.75 | -1.53 | 0.0316556 | 0.521230621 |
| *LOC106041754* | 189.50 | 106.84 | 0.83 | 0.031720154 | 0.521230621 |
| *DCTN1* | 281.53 | 188.60 | 0.58 | 0.031724294 | 0.521230621 |
| *TMEM273* | 67.04 | 26.53 | 1.35 | 0.031727411 | 0.521230621 |
| *ATP5ME* | 585.06 | 846.45 | -0.53 | 0.031906193 | 0.523531212 |
| *TWNK* | 324.45 | 161.24 | 1.01 | 0.031952422 | 0.523531212 |
| *ECI2* | 1056.70 | 1614.04 | -0.61 | 0.032051083 | 0.523531212 |
| *SLC25A42* | 34.96 | 81.16 | -1.23 | 0.032057377 | 0.523531212 |
| *CRTC1* | 70.05 | 32.87 | 1.10 | 0.032057588 | 0.523531212 |
| *LRRK1* | 27.93 | 7.16 | 1.96 | 0.032104111 | 0.523669789 |
| *CIAO1* | 80.93 | 144.42 | -0.84 | 0.032219452 | 0.524929226 |
| *USP3* | 258.02 | 373.90 | -0.54 | 0.032322489 | 0.525782552 |
| *LOC106035276* | 14.98 | 2.03 | 2.89 | 0.032368723 | 0.525782552 |
| *TMTC1* | 148.67 | 79.00 | 0.92 | 0.032386403 | 0.525782552 |
| *MBD2* | 133.16 | 81.13 | 0.71 | 0.032513233 | 0.527219881 |
| *LOC125183063* | 13.86 | 1.35 | 3.37 | 0.032674125 | 0.528196477 |
| *RFX3* | 20.13 | 6.19 | 1.72 | 0.032712544 | 0.528196477 |
| *THG1L* | 48.20 | 90.75 | -0.90 | 0.032778947 | 0.528196477 |
| *LOC125179635* | 6.13 | 0.23 | 4.51 | 0.03278281 | 0.528196477 |
| *TASP1* | 51.16 | 24.67 | 1.06 | 0.032819931 | 0.528196477 |
| *LOC106048794* | 143.01 | 80.24 | 0.84 | 0.032838553 | 0.528196477 |
| *LCAT* | 2198.79 | 3137.43 | -0.51 | 0.032842027 | 0.528196477 |
| *MFN1* | 196.02 | 300.59 | -0.62 | 0.032962041 | 0.52888567 |
| *BRMS1L* | 163.65 | 256.20 | -0.65 | 0.033006511 | 0.52888567 |
| *BTC* | 31.70 | 13.30 | 1.26 | 0.033049011 | 0.52888567 |
| *JUN* | 275.33 | 158.83 | 0.80 | 0.033050629 | 0.52888567 |
| *PIEZO1* | 40.77 | 8.62 | 2.24 | 0.033108569 | 0.52888567 |
| *LOC106039141* | 5.04 | 0.00 | 4.97 | 0.033122873 | 0.52888567 |
| *RAB23* | 98.89 | 162.19 | -0.72 | 0.033153798 | 0.52888567 |
| *LOC106030713* | 45.88 | 93.84 | -1.04 | 0.033282282 | 0.530320813 |
| *LOC106037779* | 31.09 | 10.89 | 1.51 | 0.033365474 | 0.531031769 |
| *TSGA10* | 11.20 | 1.28 | 3.12 | 0.033451523 | 0.531786504 |
| *TUBB3* | 6.29 | 0.00 | 5.28 | 0.033598924 | 0.532567672 |
| *LOC125181877* | 17.28 | 3.00 | 2.50 | 0.033627787 | 0.532567672 |
| *LOC125181749* | 36.39 | 11.10 | 1.71 | 0.033632661 | 0.532567672 |
| *HSD17B7* | 178.18 | 101.93 | 0.80 | 0.033655399 | 0.532567672 |
| *IFNGR1* | 228.37 | 151.28 | 0.60 | 0.033700594 | 0.532590848 |
| *CCDC137* | 42.30 | 79.29 | -0.90 | 0.033758972 | 0.532590848 |
| *LOC125183899* | 6.30 | 0.00 | 5.28 | 0.033772922 | 0.532590848 |
| *XPO4* | 108.61 | 172.94 | -0.67 | 0.033931998 | 0.534229031 |
| *KMT2A* | 118.10 | 55.50 | 1.10 | 0.034011915 | 0.534229031 |
| *RRP15* | 24.35 | 52.06 | -1.11 | 0.0341357 | 0.534229031 |
| *PTPN3* | 82.18 | 131.86 | -0.68 | 0.034138473 | 0.534229031 |
| *MIEF2* | 46.97 | 15.81 | 1.56 | 0.034148443 | 0.534229031 |
| *PRICKLE2* | 36.50 | 7.34 | 2.32 | 0.034164367 | 0.534229031 |
| *PDGFD* | 66.45 | 17.37 | 1.95 | 0.034175479 | 0.534229031 |
| *LOC106049228* | 48.80 | 16.61 | 1.57 | 0.034222198 | 0.534229031 |
| *RSRC1* | 20.95 | 46.71 | -1.16 | 0.034226048 | 0.534229031 |
| *MTMR7* | 3.19 | 15.24 | -2.24 | 0.034361277 | 0.535136561 |
| *CFAP97D1* | 0.00 | 5.74 | -4.84 | 0.034361932 | 0.535136561 |
| *LOC125179940* | 163.78 | 89.63 | 0.87 | 0.034466896 | 0.535186787 |
| *LMBRD1* | 41.01 | 85.66 | -1.06 | 0.034482101 | 0.535186787 |
| *ADAM33* | 34.81 | 9.80 | 1.84 | 0.034516728 | 0.535186787 |
| *ZNF462* | 2.16 | 15.63 | -2.90 | 0.034520656 | 0.535186787 |
| *LOC106047110* | 156.54 | 242.51 | -0.64 | 0.034671323 | 0.536491377 |
| *SLA* | 61.43 | 26.18 | 1.24 | 0.034704206 | 0.536491377 |
| *IMPDH2* | 599.28 | 897.43 | -0.58 | 0.034721713 | 0.536491377 |
| *BAK1* | 97.98 | 56.79 | 0.79 | 0.0348533 | 0.537920833 |
| *LOC106048085* | 123.91 | 203.97 | -0.72 | 0.034901384 | 0.538059741 |
| *HLCS* | 539.22 | 852.55 | -0.66 | 0.034999024 | 0.538961474 |
| *TMEM222* | 243.01 | 149.90 | 0.69 | 0.035177136 | 0.541099029 |
| *LOC125183589* | 0.00 | 5.56 | -4.79 | 0.035290194 | 0.542232261 |
| *P3H4* | 19.71 | 4.16 | 2.23 | 0.035422249 | 0.543467881 |
| *LOC125184742* | 5.86 | 20.21 | -1.83 | 0.035506836 | 0.543467881 |
| *PRDM4* | 185.34 | 122.28 | 0.60 | 0.035514456 | 0.543467881 |
| *ABHD5* | 558.09 | 873.94 | -0.65 | 0.035528517 | 0.543467881 |
| *CRYBG2* | 102.39 | 46.86 | 1.13 | 0.035828289 | 0.547337943 |
| *CSF3R* | 34.62 | 12.11 | 1.53 | 0.035861032 | 0.547337943 |
| *CUNH9orf64* | 65.31 | 29.81 | 1.13 | 0.035978718 | 0.548526035 |
| *N6AMT1* | 16.03 | 44.00 | -1.45 | 0.036326647 | 0.553163135 |
| *LOC106046816* | 58.58 | 24.09 | 1.29 | 0.036363234 | 0.553163135 |
| *KYAT3* | 420.78 | 612.24 | -0.54 | 0.036425088 | 0.553492477 |
| *DNLZ* | 33.21 | 67.79 | -1.01 | 0.036587281 | 0.554578036 |
| *FBXO32* | 100.61 | 48.64 | 1.05 | 0.036617377 | 0.554578036 |
| *RWDD4* | 111.68 | 197.94 | -0.82 | 0.036714712 | 0.555441148 |
| *POF1B* | 15.60 | 2.77 | 2.49 | 0.036889308 | 0.557385811 |
| *GJB2* | 61.63 | 30.25 | 1.03 | 0.037013098 | 0.557385811 |
| *NGEF* | 529.40 | 299.13 | 0.82 | 0.037034599 | 0.557385811 |
| *LOC125184277* | 0.00 | 6.85 | -5.10 | 0.03703716 | 0.557385811 |
| *UCHL5* | 98.59 | 156.99 | -0.67 | 0.03704569 | 0.557385811 |
| *DOLPP1* | 33.36 | 66.35 | -1.00 | 0.03722662 | 0.559496585 |
| *CLIC2* | 427.55 | 281.54 | 0.60 | 0.037513103 | 0.561698786 |
| *ALDH8A1* | 510.16 | 766.83 | -0.59 | 0.037519839 | 0.561698786 |
| *DAPK1* | 21.28 | 48.95 | -1.20 | 0.03753475 | 0.561698786 |
| *MBNL1* | 921.39 | 609.21 | 0.60 | 0.037536347 | 0.561698786 |
| *LOC106029611* | 4.89 | 0.00 | 4.93 | 0.037598739 | 0.561714739 |
| *CBLB* | 59.17 | 25.18 | 1.24 | 0.037682526 | 0.561714739 |
| *GNL2* | 339.84 | 508.84 | -0.58 | 0.037705131 | 0.561714739 |
| *LOC106042848* | 253.88 | 411.40 | -0.70 | 0.037727395 | 0.561714739 |
| *SPRED1* | 92.45 | 150.00 | -0.70 | 0.037814618 | 0.561714739 |
| *NSUN4* | 70.79 | 120.23 | -0.76 | 0.037825608 | 0.561714739 |
| *RNF128* | 239.86 | 360.46 | -0.58 | 0.037855135 | 0.561714739 |
| *LOC125181435* | 397.16 | 238.53 | 0.74 | 0.037863825 | 0.561714739 |
| *GRID1* | 4.08 | 0.00 | 4.66 | 0.037965787 | 0.562621088 |
| *SYTL2* | 70.61 | 27.14 | 1.38 | 0.038074208 | 0.563041529 |
| *PCNT* | 49.63 | 23.23 | 1.10 | 0.038075954 | 0.563041529 |
| *LOC106034806* | 63.28 | 110.00 | -0.79 | 0.038161182 | 0.563124181 |
| *OVOL2* | 19.65 | 5.53 | 1.82 | 0.038222393 | 0.563124181 |
| *FAM53B* | 50.14 | 19.40 | 1.38 | 0.038245146 | 0.563124181 |
| *DUOX2* | 5.88 | 0.00 | 5.19 | 0.038307801 | 0.563124181 |
| *ARSG* | 158.68 | 92.70 | 0.78 | 0.03833226 | 0.563124181 |
| *GPR27* | 4.84 | 0.00 | 4.91 | 0.038389709 | 0.563124181 |
| *LOC106031011* | 665.69 | 1094.68 | -0.72 | 0.038397667 | 0.563124181 |
| *CPSF6* | 73.62 | 121.97 | -0.73 | 0.038432547 | 0.563124181 |
| *WSB1* | 313.46 | 489.25 | -0.64 | 0.038580886 | 0.563124181 |
| *ITCH* | 173.88 | 251.55 | -0.53 | 0.038629879 | 0.563124181 |
| *CMTR2* | 74.25 | 36.55 | 1.03 | 0.038650537 | 0.563124181 |
| *KCNK1* | 79.78 | 129.50 | -0.70 | 0.038668581 | 0.563124181 |
| *LOC106048003* | 6.69 | 0.49 | 3.87 | 0.038689301 | 0.563124181 |
| *NUP214* | 109.14 | 60.08 | 0.85 | 0.038797433 | 0.563124181 |
| *BIN2* | 66.07 | 30.89 | 1.10 | 0.038798513 | 0.563124181 |
| *LOC125181711* | 11808.63 | 16576.09 | -0.49 | 0.038800391 | 0.563124181 |
| *KCNE2* | 0.00 | 6.65 | -5.05 | 0.038831421 | 0.563124181 |
| *CYGB* | 807.54 | 491.01 | 0.72 | 0.038862999 | 0.563124181 |
| *PICK1* | 32.67 | 13.76 | 1.25 | 0.038908132 | 0.563124181 |
| *ABLIM1* | 243.83 | 147.17 | 0.73 | 0.039023474 | 0.563124181 |
| *THOC2* | 556.34 | 802.05 | -0.53 | 0.039065323 | 0.563124181 |
| *RPP25L* | 133.19 | 226.58 | -0.77 | 0.039151922 | 0.563124181 |
| *PRPSAP1* | 100.91 | 56.90 | 0.82 | 0.039155048 | 0.563124181 |
| *FNIP1* | 151.12 | 72.69 | 1.06 | 0.039156126 | 0.563124181 |
| *P2RX7* | 13.38 | 1.58 | 3.08 | 0.039220463 | 0.563124181 |
| *LOC106032277* | 4.74 | 0.00 | 4.88 | 0.039267425 | 0.563124181 |
| *PLCB2* | 37.33 | 12.65 | 1.58 | 0.039270767 | 0.563124181 |
| *LOC106032520* | 38.31 | 12.49 | 1.62 | 0.039322879 | 0.563124181 |
| *LOC106042760* | 312.43 | 442.68 | -0.50 | 0.039405248 | 0.563124181 |
| *LOC106038418* | 103.08 | 57.46 | 0.84 | 0.039413543 | 0.563124181 |
| *NFIL3* | 239.44 | 158.69 | 0.59 | 0.039473344 | 0.563124181 |
| *GPI* | 674.81 | 1079.71 | -0.68 | 0.039504773 | 0.563124181 |
| *ALPK2* | 302.88 | 445.67 | -0.56 | 0.03955125 | 0.563124181 |
| *LOC125180351* | 3.73 | 21.39 | -2.52 | 0.039606992 | 0.563124181 |
| *PSMC4* | 529.75 | 771.97 | -0.54 | 0.039664982 | 0.563124181 |
| *SNX13* | 44.16 | 77.72 | -0.81 | 0.039673744 | 0.563124181 |
| *DECR2* | 1653.56 | 2482.95 | -0.59 | 0.03974328 | 0.563124181 |
| *LMTK2* | 63.47 | 27.94 | 1.20 | 0.039850318 | 0.563124181 |
| *UVRAG* | 143.84 | 220.89 | -0.61 | 0.0399006 | 0.563124181 |
| *LOC106037315* | 93.96 | 55.19 | 0.77 | 0.039927353 | 0.563124181 |
| *FAM3C* | 166.77 | 247.42 | -0.57 | 0.039934496 | 0.563124181 |
| *LOC106041910* | 10.80 | 1.14 | 3.18 | 0.039938097 | 0.563124181 |
| *PCYOX1L* | 44.26 | 17.93 | 1.31 | 0.039949506 | 0.563124181 |
| *LOC106042383* | 17.37 | 2.71 | 2.70 | 0.039963905 | 0.563124181 |
| *TGM2* | 722.91 | 483.88 | 0.58 | 0.040053387 | 0.563124181 |
| *TUBG1* | 135.04 | 84.81 | 0.67 | 0.04005475 | 0.563124181 |
| *LOC106039600* | 76.93 | 123.49 | -0.68 | 0.040134702 | 0.563124181 |
| *TNIP1* | 378.44 | 258.18 | 0.55 | 0.04018068 | 0.563124181 |
| *SOGA3* | 9.71 | 0.76 | 3.62 | 0.040241166 | 0.563124181 |
| *CCDC134* | 77.73 | 33.21 | 1.23 | 0.040321351 | 0.563124181 |
| *SDHA* | 660.15 | 975.60 | -0.57 | 0.040345571 | 0.563124181 |
| *SEMA4A* | 45.81 | 11.90 | 1.95 | 0.040361644 | 0.563124181 |
| *TNRC6C* | 131.39 | 66.40 | 0.99 | 0.04039939 | 0.563124181 |
| *LOC125182806* | 255.66 | 138.63 | 0.88 | 0.040400293 | 0.563124181 |
| *WASF1* | 0.00 | 4.45 | -4.47 | 0.040454086 | 0.563125787 |
| *LRRC34* | 7.30 | 0.45 | 4.01 | 0.040587278 | 0.563329654 |
| *SEC61A2* | 35.92 | 13.74 | 1.39 | 0.040598239 | 0.563329654 |
| *COL6A2* | 200.79 | 99.74 | 1.01 | 0.040702164 | 0.563329654 |
| *INAVA* | 9.53 | 1.20 | 3.03 | 0.040728168 | 0.563329654 |
| *LOC106034487* | 4.19 | 0.00 | 4.71 | 0.040732404 | 0.563329654 |
| *RHPN2* | 161.12 | 257.01 | -0.68 | 0.040765958 | 0.563329654 |
| *LOC125179694* | 2648.02 | 1848.66 | 0.52 | 0.040823367 | 0.563329654 |
| *STOM* | 57.04 | 25.53 | 1.15 | 0.040823722 | 0.563329654 |
| *LOC106048305* | 38.23 | 12.15 | 1.66 | 0.040857354 | 0.563329654 |
| *PKD1* | 41.08 | 14.16 | 1.54 | 0.040889247 | 0.563329654 |
| *VAPB* | 627.76 | 894.29 | -0.51 | 0.040918839 | 0.563329654 |
| *UROD* | 72.52 | 121.39 | -0.75 | 0.041008985 | 0.56400669 |
| *LOC125181830* | 22.72 | 7.38 | 1.61 | 0.04121516 | 0.566215989 |
| *FOXN4* | 4.82 | 0.00 | 4.89 | 0.041308121 | 0.566215989 |
| *TRIM66* | 7.03 | 0.41 | 4.01 | 0.041339305 | 0.566215989 |
| *MTRES1* | 114.64 | 173.30 | -0.60 | 0.041369286 | 0.566215989 |
| *GARS1* | 272.23 | 409.99 | -0.60 | 0.041375266 | 0.566215989 |
| *LOC106036305* | 135.32 | 71.39 | 0.92 | 0.041440615 | 0.566342824 |
| *ABCF2* | 149.76 | 223.89 | -0.58 | 0.04146681 | 0.566342824 |
| *BICC1* | 72.69 | 37.96 | 0.94 | 0.041536756 | 0.566735902 |
| *RTN4IP1* | 138.58 | 210.16 | -0.60 | 0.041784953 | 0.569557874 |
| *CD44* | 89.15 | 36.07 | 1.31 | 0.041950773 | 0.570512148 |
| *RAB30* | 276.77 | 432.66 | -0.65 | 0.04196661 | 0.570512148 |
| *NIBAN2* | 139.40 | 76.53 | 0.87 | 0.042066961 | 0.570580459 |
| *LOC106032457* | 13.03 | 1.56 | 3.08 | 0.042107068 | 0.570580459 |
| *CDK5RAP2* | 53.07 | 20.54 | 1.36 | 0.042108647 | 0.570580459 |
| *MCOLN3* | 0.00 | 5.18 | -4.69 | 0.042236993 | 0.571756811 |
| *RNF150* | 85.90 | 40.59 | 1.10 | 0.04241218 | 0.57356433 |
| *TUSC3* | 74.73 | 121.89 | -0.70 | 0.042473073 | 0.573824131 |
| *CCND3* | 66.58 | 33.93 | 0.97 | 0.042546621 | 0.574254247 |
| *NCF1* | 54.15 | 24.51 | 1.15 | 0.042691012 | 0.574592918 |
| *PRELID1* | 1355.07 | 907.13 | 0.58 | 0.042726181 | 0.574592918 |
| *ST8SIA4* | 27.62 | 8.32 | 1.73 | 0.042738661 | 0.574592918 |
| *ALAS1* | 202.82 | 125.10 | 0.69 | 0.042791753 | 0.574615159 |
| *GLB1L2* | 38.12 | 15.92 | 1.26 | 0.042823793 | 0.574615159 |
| *FOXO4* | 257.96 | 173.83 | 0.57 | 0.042917985 | 0.575318302 |
| *FBXW7* | 184.54 | 101.47 | 0.87 | 0.043039628 | 0.576276257 |
| *POLA1* | 83.64 | 190.96 | -1.20 | 0.043086103 | 0.576276257 |
| *TMEM61* | 23.98 | 6.48 | 1.87 | 0.043115025 | 0.576276257 |
| *LOC106029858* | 10.28 | 1.49 | 2.85 | 0.043229191 | 0.577241776 |
| *PTPRF* | 163.65 | 85.16 | 0.94 | 0.043418382 | 0.578437822 |
| *JUND* | 435.90 | 258.78 | 0.75 | 0.043425016 | 0.578437822 |
| *DLD* | 1175.47 | 1658.02 | -0.50 | 0.043444811 | 0.578437822 |
| *FAT1* | 78.61 | 44.68 | 0.81 | 0.04354594 | 0.579224113 |
| *CDK15* | 0.00 | 5.24 | -4.71 | 0.043651196 | 0.580050783 |
| *LOC106047166* | 149.43 | 68.83 | 1.12 | 0.043741372 | 0.580050783 |
| *ELF3* | 77.81 | 25.77 | 1.60 | 0.043823097 | 0.580050783 |
| *LOC125181497* | 5.58 | 0.00 | 5.11 | 0.043863261 | 0.580050783 |
| *CABP1* | 254.17 | 171.35 | 0.57 | 0.043865246 | 0.580050783 |
| *LOC106032013* | 184.09 | 295.24 | -0.68 | 0.043871697 | 0.580050783 |
| *PWP1* | 113.76 | 177.49 | -0.64 | 0.043937103 | 0.580050783 |
| *LOC106049750* | 126.98 | 56.39 | 1.17 | 0.044006158 | 0.580050783 |
| *COMMD5* | 152.22 | 232.32 | -0.61 | 0.044014109 | 0.580050783 |
| *LOC106038675* | 171.08 | 299.91 | -0.81 | 0.04411988 | 0.580050783 |
| *ABCA3* | 110.01 | 52.88 | 1.05 | 0.044120933 | 0.580050783 |
| *DPYSL2* | 86.75 | 42.24 | 1.04 | 0.044149381 | 0.580050783 |
| *ALDH7A1* | 1413.05 | 2132.49 | -0.59 | 0.044155823 | 0.580050783 |
| *LOC106049374* | 41.80 | 14.62 | 1.52 | 0.044287963 | 0.580959694 |
| *TRAPPC10* | 52.97 | 90.39 | -0.76 | 0.044309412 | 0.580959694 |
| *PPCDC* | 89.48 | 52.99 | 0.75 | 0.044367701 | 0.581170448 |
| *SLC12A9* | 45.73 | 15.12 | 1.60 | 0.044488152 | 0.582194284 |
| *CLTRN* | 620.93 | 343.40 | 0.86 | 0.044652876 | 0.583427532 |
| *ITIH4* | 1226.30 | 1773.46 | -0.53 | 0.044746839 | 0.583427532 |
| *COMMD2* | 245.01 | 344.83 | -0.49 | 0.044755781 | 0.583427532 |
| *LZTS1* | 5.56 | 0.00 | 5.11 | 0.044785411 | 0.583427532 |
| *MFSD4A* | 19.45 | 4.20 | 2.20 | 0.044794284 | 0.583427532 |
| *LOC106031104* | 8.50 | 0.68 | 3.67 | 0.044885905 | 0.584068288 |
| *LOC125183415* | 0.00 | 5.11 | -4.67 | 0.045050771 | 0.584875269 |
| *PLK3* | 14.31 | 3.93 | 1.86 | 0.045055088 | 0.584875269 |
| *QRFPR* | 13.54 | 2.12 | 2.68 | 0.045120714 | 0.584875269 |
| *SPIRE2* | 25.01 | 3.42 | 2.86 | 0.045154386 | 0.584875269 |
| *CRISPLD2* | 89.62 | 42.86 | 1.08 | 0.045169132 | 0.584875269 |
| *VOPP1* | 4.66 | 0.00 | 4.85 | 0.045245106 | 0.584875269 |
| *CRYAB* | 36.39 | 14.15 | 1.37 | 0.045254119 | 0.584875269 |
| *RNF41* | 154.90 | 90.36 | 0.78 | 0.045324219 | 0.584875269 |
| *GPAM* | 483.90 | 734.68 | -0.60 | 0.045330276 | 0.584875269 |
| *LOC106038582* | 43.40 | 12.26 | 1.82 | 0.045494608 | 0.586445942 |
| *LOC106032592* | 39.45 | 79.69 | -1.01 | 0.045635946 | 0.587717558 |
| *ZDHHC9* | 278.26 | 178.38 | 0.64 | 0.045768015 | 0.588238003 |
| *LOC125179653* | 9.06 | 0.61 | 3.88 | 0.045797273 | 0.588238003 |
| *IQSEC3* | 47.41 | 14.16 | 1.74 | 0.045854742 | 0.588238003 |
| *HUS1* | 88.03 | 142.04 | -0.68 | 0.045872684 | 0.588238003 |
| *SYCP1* | 14.28 | 3.55 | 1.99 | 0.045970492 | 0.588238003 |
| *LOC106031613* | 958.44 | 1405.58 | -0.55 | 0.045990413 | 0.588238003 |
| *KLHL32* | 7.23 | 0.45 | 3.98 | 0.045996008 | 0.588238003 |
| *TMEM25* | 4.50 | 0.00 | 4.80 | 0.046025236 | 0.588238003 |
| *ERCC3* | 72.85 | 126.19 | -0.79 | 0.046132477 | 0.588238003 |
| *UBR2* | 34.98 | 71.81 | -1.04 | 0.046196091 | 0.588238003 |
| *GALM* | 270.66 | 405.45 | -0.59 | 0.046233134 | 0.588238003 |
| *XRCC6* | 97.67 | 151.49 | -0.63 | 0.046337212 | 0.588238003 |
| *LOC106034885* | 136.48 | 215.08 | -0.66 | 0.046346122 | 0.588238003 |
| *RETSAT* | 959.66 | 529.27 | 0.86 | 0.046363117 | 0.588238003 |
| *SNX17* | 746.57 | 465.15 | 0.68 | 0.046461894 | 0.588238003 |
| *ZDHHC18* | 22.13 | 7.21 | 1.60 | 0.04646199 | 0.588238003 |
| *COL1A1* | 492.70 | 156.60 | 1.66 | 0.046473031 | 0.588238003 |
| *GEMIN8* | 93.60 | 146.07 | -0.64 | 0.046488193 | 0.588238003 |
| *AOX1* | 1363.29 | 1980.03 | -0.54 | 0.046546402 | 0.588433711 |
| *LOC125181834* | 15.80 | 2.84 | 2.45 | 0.046665965 | 0.588514147 |
| *LOC125182017* | 0.00 | 5.14 | -4.68 | 0.046714454 | 0.588514147 |
| *STX5* | 84.94 | 43.95 | 0.95 | 0.046735037 | 0.588514147 |
| *CMTM7* | 80.30 | 42.22 | 0.93 | 0.046779327 | 0.588514147 |
| *TXNDC11* | 185.43 | 298.53 | -0.69 | 0.046798078 | 0.588514147 |
| *LOC106033656* | 233.84 | 341.32 | -0.55 | 0.046899999 | 0.588514147 |
| *LOC106035751* | 7.11 | 0.52 | 3.97 | 0.046908912 | 0.588514147 |
| *STEAP4* | 123.87 | 193.19 | -0.64 | 0.046937498 | 0.588514147 |
| *DNAJC5G* | 271.12 | 143.49 | 0.92 | 0.047142957 | 0.589744939 |
| *PLCL1* | 19.53 | 45.54 | -1.21 | 0.047234911 | 0.589744939 |
| *LOC106042626* | 11.80 | 1.85 | 2.65 | 0.047253468 | 0.589744939 |
| *TNFSF4* | 5.43 | 0.00 | 5.08 | 0.047295261 | 0.589744939 |
| *TCTN3* | 112.87 | 66.62 | 0.76 | 0.047309056 | 0.589744939 |
| *ERBIN* | 180.04 | 265.88 | -0.56 | 0.047330818 | 0.589744939 |
| *PTGR1* | 1568.95 | 2773.80 | -0.82 | 0.047349791 | 0.589744939 |
| *ZSWIM5* | 55.75 | 28.42 | 0.96 | 0.047398357 | 0.589744939 |
| *ARHGAP35* | 327.89 | 216.94 | 0.60 | 0.047423593 | 0.589744939 |
| *LOC106033532* | 460.22 | 245.92 | 0.91 | 0.047547594 | 0.590250436 |
| *POFUT2* | 269.48 | 185.12 | 0.54 | 0.047621744 | 0.590638332 |
| *LYSMD1* | 156.29 | 72.81 | 1.10 | 0.04770592 | 0.591149781 |
| *ARRDC1* | 89.18 | 43.21 | 1.05 | 0.047914947 | 0.591229868 |
| *PODN* | 93.00 | 181.13 | -0.96 | 0.047919082 | 0.591229868 |
| *LOC106049427* | 118.07 | 72.74 | 0.71 | 0.047936645 | 0.591229868 |
| *LOC106031757* | 5.41 | 0.00 | 5.07 | 0.047969282 | 0.591229868 |
| *IL6R* | 517.12 | 307.62 | 0.75 | 0.048011186 | 0.591229868 |
| *SAMD13* | 12.67 | 2.27 | 2.46 | 0.048061396 | 0.591229868 |
| *BST1* | 62.58 | 111.23 | -0.82 | 0.048071443 | 0.591229868 |
| *PMVK* | 518.91 | 768.06 | -0.57 | 0.048073368 | 0.591229868 |
| *LOC106040417* | 143.12 | 225.73 | -0.66 | 0.048128147 | 0.591229868 |
| *WBP1* | 450.12 | 243.21 | 0.89 | 0.048141838 | 0.591229868 |
| *TRIM65* | 61.55 | 27.76 | 1.15 | 0.048189338 | 0.591285758 |
| *TNRC18* | 217.62 | 123.31 | 0.82 | 0.048301848 | 0.592138501 |
| *CLCN6* | 29.06 | 8.58 | 1.77 | 0.048394712 | 0.592418474 |
| *LGALS2* | 59.94 | 5.55 | 3.43 | 0.048452135 | 0.592418474 |
| *LOC106037228* | 0.62 | 9.35 | -3.97 | 0.048479723 | 0.592418474 |
| *PMPCB* | 291.30 | 451.30 | -0.63 | 0.048496813 | 0.592418474 |
| *UTRN* | 91.39 | 50.41 | 0.86 | 0.04862979 | 0.593516239 |
| *WDR53* | 186.52 | 280.19 | -0.59 | 0.04882058 | 0.59531703 |
| *ARID5A* | 68.41 | 35.48 | 0.95 | 0.048910498 | 0.595867645 |
| *HK3* | 22.58 | 7.89 | 1.53 | 0.048974923 | 0.595867645 |
| *DGCR2* | 89.02 | 49.42 | 0.85 | 0.049000155 | 0.595867645 |
| *DOHH* | 840.08 | 482.23 | 0.80 | 0.049038864 | 0.595867645 |
| *PAIP2B* | 47.64 | 19.29 | 1.29 | 0.049250529 | 0.59714401 |
| *RPL22L1* | 535.09 | 824.83 | -0.62 | 0.049359747 | 0.59714401 |
| *GCNT4* | 46.95 | 21.94 | 1.11 | 0.049368472 | 0.59714401 |
| *FAM177A1* | 158.88 | 92.56 | 0.78 | 0.049372678 | 0.59714401 |
| *SATB1* | 55.52 | 27.01 | 1.04 | 0.049424771 | 0.59714401 |
| *SIPA1L1* | 33.63 | 12.97 | 1.38 | 0.049474846 | 0.59714401 |
| *LOC106048967* | 7.89 | 0.57 | 3.71 | 0.049490907 | 0.59714401 |
| *RNF2* | 6.38 | 23.52 | -1.84 | 0.049681554 | 0.598919404 |
| *TTC7B* | 25.75 | 50.37 | -0.96 | 0.049779845 | 0.599135306 |
| *CTRL* | 5.75 | 0.00 | 5.15 | 0.049800453 | 0.599135306 |
| *DOCK2* | 63.37 | 30.46 | 1.06 | 0.049830023 | 0.599135306 |
| *IRF1* | 189.52 | 105.65 | 0.84 | 0.049931146 | 0.599827299 |
| *SRA1* | 686.11 | 443.93 | 0.63 | 0.049981825 | 0.599912628 |
