# Supplementary material for: Comprehensive analysis of histophysiology, transcriptomics and metabolomics in goslings exposed to gossypol acetate: unraveling hepatotoxic mechanisms
Source: Front Vet Sci. 2025 Jan 21;12:1527284. doi: 10.3389/fvets.2025.1527284 (PMC11792171; doi:10.3389/fvets.2025.1527284)
Supplement: Supplementary file 1 [file Data_Sheet_1.zip › supplementary materials/Table S5. Identifiable metabolites in the liver of goslings from the GA50 and control groups.docx]

**Table S5.** Identifiable metabolites in the liver of goslings from the GA50 and control groups.

| Metabolites | mz | FC | *P*-value | FDR | VIP | pos/neg |
| --- | --- | --- | --- | --- | --- | --- |
| 2-Pyrrolidinone | 100.0761 | 4.54 | 0.08085 | 0.289 | 1.16 | pos |
| Triethylamine | 101.0601 | 1.09 | 0.48138 | 0.712 | 0.60 | pos |
| Isovaleric acid | 102.1282 | 1.45 | 0.11107 | 0.337 | 1.15 | pos |
| Betaine aldehyde | 102.0912 | 0.68 | 0.18023 | 0.429 | 1.00 | pos |
| Valeric acid | 102.0554 | 0.9 | 0.14134 | 0.379 | 0.88 | pos |
| 1-Aminocyclopropanecarboxylic acid | 102.0552 | 1.16 | 0.00112 | 0.041 | 1.86 | pos |
| Succinic acid semialdehyde | 102.034 | 1.43 | 0.42669 | 0.673 | 0.56 | pos |
| Acetoacetic acid | 103.0393 | 0.93 | 0.22950 | 0.487 | 0.66 | pos |
| Dimethylglycine | 104.1071 | 0.94 | 0.53242 | 0.747 | 0.41 | pos |
| 2-Aminoisobutyric acid | 104.0709 | 0.7 | 0.14670 | 0.386 | 1.03 | pos |
| L-Serine | 106.0506 | 1.18 | 0.31423 | 0.575 | 0.80 | pos |
| Benzaldehyde | 107.0495 | 0.88 | 0.07636 | 0.282 | 1.15 | pos |
| m-Cresol | 109.0227 | 0.93 | 0.22781 | 0.485 | 0.86 | pos |
| Hydroquinone | 110.0203 | 0.41 | 0.24587 | 0.504 | 0.87 | pos |
| Cytosine | 112.051 | 1 | 0.79009 | 0.902 | 0.21 | pos |
| Uracil | 113.0348 | 1.15 | 0.71509 | 0.862 | 0.32 | pos |
| 1-Pyrroline-2-carboxylic acid | 114.0666 | 1.52 | 0.80973 | 0.912 | 0.22 | pos |
| 2-Heptanone | 113.9636 | 1.65 | 0.09191 | 0.309 | 1.17 | pos |
| Epsilon-caprolactam | 114.0916 | 1.25 | 0.49240 | 0.720 | 0.47 | pos |
| epsilon-Caprolactone | 114.0668 | 0.79 | 0.51821 | 0.737 | 0.43 | pos |
| L-Prolinamide | 115.0869 | 0.72 | 0.16402 | 0.409 | 0.93 | pos |
| 5-Aminopentanoic acid | 118.0862 | 1.19 | 0.14351 | 0.382 | 0.90 | pos |
| L-2,4-diaminobutyric acid | 118.0651 | 1.08 | 0.10728 | 0.332 | 1.20 | pos |
| Aminomalonic acid | 119.0164 | 2.54 | 0.00023 | 0.019 | 2.03 | pos |
| 2-Methylserine | 119.0494 | 0.78 | 0.01352 | 0.131 | 1.55 | pos |
| 5-Hydroxypentanoic acid | 119.0732 | 1.3 | 0.00147 | 0.046 | 1.87 | pos |
| L-Allothreonine | 120.0657 | 1.18 | 0.03150 | 0.188 | 1.42 | pos |
| 1-Methylnicotinamide | 120.0654 | 1.29 | 0.06943 | 0.269 | 1.26 | pos |
| L-Threonine | 120.0656 | 1.2 | 0.01687 | 0.144 | 1.58 | pos |
| Styrene Oxide | 121.0648 | 1.61 | 0.28787 | 0.549 | 0.80 | pos |
| (S)-1-Phenylethanol | 122.0713 | 2.64 | 0.00001 | 0.003 | 2.18 | pos |
| Niacinamide | 123.055 | 0.71 | 0.20306 | 0.456 | 0.85 | pos |
| Nicotinic acid | 124.0398 | 0.53 | 0.05784 | 0.246 | 1.30 | pos |
| Isonicotinic acid | 124.0399 | 1.25 | 0.06265 | 0.256 | 1.36 | pos |
| 3-Hydroxybenzyl alcohol glucoside | 124.0875 | 2.77 | 0.03315 | 0.192 | 1.34 | pos |
| Aminohydroquinone | 126.0549 | 0.8 | 0.62833 | 0.808 | 0.40 | pos |
| Thymine | 125.9865 | 0.39 | 0.01221 | 0.126 | 1.62 | pos |
| 4-Aminocatechol | 126.0547 | 0.36 | 0.31580 | 0.576 | 0.51 | pos |
| Imidazoleacetic acid | 127.0504 | 0.99 | 0.73685 | 0.874 | 0.27 | pos |
| 1,2,3-Trihydroxybenzene | 127.0399 | 0.9 | 0.65606 | 0.825 | 0.27 | pos |
| Maltol | 127.0385 | 0.59 | 0.12282 | 0.353 | 1.02 | pos |
| 5-Hydroxymethyl-2-furancarboxaldehyde | 127.0389 | 1.39 | 0.24709 | 0.506 | 0.73 | pos |
| Dihydrothymine | 128.0711 | 1.35 | 0.65668 | 0.826 | 0.31 | pos |
| (S)-2,3,4,5-tetrahydropyridine-2-carboxylate | 128.0703 | 1.11 | 0.96672 | 0.985 | 0.06 | pos |
| D-1-Piperideine-2-carboxylic acid | 128.0702 | 1.24 | 0.91575 | 0.963 | 0.17 | pos |
| L-Glutamine | 129.0655 | 0.84 | 0.31123 | 0.572 | 0.84 | pos |
| (S)-Piperidine-2-carboxamide | 129.1024 | 0.86 | 0.47168 | 0.705 | 0.51 | pos |
| Pipecolic acid | 130.0854 | 1.31 | 0.09045 | 0.307 | 1.24 | pos |
| Pyrrolidonecarboxylic acid | 130.0511 | 0.71 | 0.49742 | 0.723 | 0.39 | pos |
| Adipate semialdehyde | 130.0491 | 1.1 | 0.75225 | 0.882 | 0.21 | pos |
| Pyrroline hydroxycarboxylic acid | 130.049 | 0.63 | 0.06062 | 0.252 | 1.23 | pos |
| 5-Amino-2-oxopentanoic acid | 131.0541 | 0.62 | 0.01397 | 0.133 | 1.49 | pos |
| cis-4-Hydroxy-D-proline | 131.1627 | 1.26 | 0.89374 | 0.953 | 0.15 | pos |
| Heptanoic acid | 130.9663 | 1.78 | 0.09478 | 0.312 | 1.10 | pos |
| 7-Methyladenine | 132.0615 | 1.16 | 0.04894 | 0.228 | 1.27 | pos |
| 5-Aminolevulinic acid | 132.0616 | 0.92 | 0.85344 | 0.935 | 0.04 | pos |
| L-Ribulose | 133.0504 | 0.94 | 0.56574 | 0.768 | 0.45 | pos |
| Ornithine | 133.0972 | 22.7 | 0.26065 | 0.520 | 0.81 | pos |
| Chavicol | 135.0805 | 0.96 | 0.74718 | 0.879 | 0.12 | pos |
| Dopamine | 136.0746 | 0.88 | 0.62865 | 0.809 | 0.28 | pos |
| 4-Hydroxyphenylacetaldehyde | 136.0624 | 1.3 | 0.00057 | 0.029 | 1.98 | pos |
| p-Aminobenzoic acid | 137.0452 | 1.34 | 0.00070 | 0.032 | 1.96 | pos |
| Trigonelline | 138.0544 | 0.92 | 0.79870 | 0.907 | 0.20 | pos |
| 2-Aminobenzoic acid | 138.0548 | 1 | 0.73087 | 0.870 | 0.14 | pos |
| (3Z,6Z)-3,6-Nonadienal | 138.1026 | 1.54 | 0.01207 | 0.126 | 1.68 | pos |
| Acetylphosphate | 139.9876 | 0.83 | 0.21484 | 0.471 | 0.95 | pos |
| 6-Oxo-1,4,5,6-tetrahydronicotinate | 142.0532 | 1.58 | 0.04914 | 0.228 | 1.24 | pos |
| 5-Hydroxymethyluracil | 141.958 | 2.89 | 0.00118 | 0.041 | 1.90 | pos |
| O-Phosphoethanolamine | 141.9588 | 1.2 | 0.83407 | 0.925 | 0.15 | pos |
| 5-Methylbarbiturate | 143.0487 | 0.8 | 0.28468 | 0.545 | 0.69 | pos |
| N-methyl-L-glutamic Acid | 144.065 | 0.49 | 0.00348 | 0.071 | 1.73 | pos |
| 5-(2-Hydroxyethyl)-4-methylthiazole | 144.048 | 0.6 | 0.01126 | 0.121 | 1.58 | pos |
| 2-Naphthol | 144.0658 | 1.01 | 0.76370 | 0.888 | 0.14 | pos |
| 2-Keto-glutaramic acid | 145.0505 | 0.54 | 0.22272 | 0.480 | 0.90 | pos |
| 3-Hydroxymethylglutaric acid | 145.0486 | 1.08 | 0.74356 | 0.877 | 0.24 | pos |
| 2-Hydroxyadipic acid | 145.0497 | 0.73 | 0.32938 | 0.589 | 0.57 | pos |
| Anabasine | 144.9817 | 0.69 | 0.01055 | 0.119 | 1.52 | pos |
| 2-Keto-6-aminocaproate | 146.0817 | 1.12 | 0.43513 | 0.679 | 0.51 | pos |
| 1-Deoxynojirimycin | 146.0807 | 1.3 | 0.23275 | 0.490 | 0.79 | pos |
| 1H-Indole-3-carboxaldehyde | 146.0594 | 1.86 | 0.03167 | 0.189 | 1.49 | pos |
| (S)-5-Amino-3-oxohexanoate | 146.0819 | 1.83 | 0.00000 | 0.002 | 2.22 | pos |
| (S)-2-amino-6-oxohexanoate | 146.081 | 0.87 | 0.41877 | 0.667 | 0.59 | pos |
| L-Lysine | 147.1128 | 0.99 | 0.96553 | 0.985 | 0.19 | pos |
| m-Coumaric acid | 147.0441 | 1.12 | 0.76651 | 0.890 | 0.15 | pos |
| (2R,5S)-2,5-Diaminohexanoate | 147.113 | 1.05 | 0.91134 | 0.961 | 0.11 | pos |
| Oxoglutaric acid | 147.0309 | 0.79 | 0.21857 | 0.475 | 0.76 | pos |
| 4-Hydroxycinnamic acid | 146.9803 | 0.41 | 0.10742 | 0.332 | 1.05 | pos |
| L-Methionine (R)-S-oxide | 148.0426 | 1.5 | 0.08313 | 0.294 | 1.29 | pos |
| O-Acetylserine | 148.0601 | 1.28 | 0.51812 | 0.737 | 0.50 | pos |
| D-beta-Phenylalanine | 148.0757 | 0.97 | 0.90079 | 0.956 | 0.02 | pos |
| (S)-2-Methylmalate | 148.0273 | 0.94 | 0.77497 | 0.895 | 0.16 | pos |
| Racemethionine | 150.0569 | 1.31 | 0.51484 | 0.735 | 0.41 | pos |
| L-Arabinose | 151.0978 | 23.09 | 0.16298 | 0.408 | 1.02 | pos |
| Guanine | 152.0563 | 0.93 | 0.47999 | 0.711 | 0.43 | pos |
| Xanthine | 153.0415 | 1.19 | 0.00059 | 0.029 | 1.85 | pos |
| D-Arabitol | 152.9949 | 1.37 | 0.01405 | 0.133 | 1.49 | pos |
| 3-Hydroxyphenylacetic acid | 152.9943 | 0.92 | 0.04519 | 0.220 | 1.34 | pos |
| N-Acetylhistamine | 154.0976 | 0.98 | 0.74675 | 0.879 | 0.16 | pos |
| p-Octopamine | 154.0862 | 1.03 | 0.73424 | 0.873 | 0.34 | pos |
| 2,3-Butanediol | 155.0704 | 1.43 | 0.34434 | 0.603 | 0.64 | pos |
| Gentisic acid | 154.9881 | 1.01 | 0.75643 | 0.884 | 0.22 | pos |
| (1R,6S)-6-Amino-5-oxocyclohex-2-ene-1-carboxylate | 156.0656 | 0.94 | 0.05486 | 0.240 | 1.39 | pos |
| (3S,5S)-Carbapenam-3-carboxylic acid | 156.0655 | 1.07 | 0.81660 | 0.915 | 0.09 | pos |
| Uracil 5-carboxylate | 156.0141 | 0.95 | 0.79106 | 0.903 | 0.35 | pos |
| Phosphoglycolic acid | 156.9907 | 1.19 | 0.22322 | 0.480 | 0.88 | pos |
| Pelargonic acid | 158.1545 | 1.16 | 0.45815 | 0.696 | 0.59 | pos |
| Serotonin | 159.0917 | 0.72 | 0.00851 | 0.108 | 1.64 | pos |
| Pimelic acid | 159.9685 | 1.5 | 0.22512 | 0.482 | 0.77 | pos |
| Indoleacetaldehyde | 160.0758 | 1.38 | 0.23153 | 0.489 | 0.95 | pos |
| 4-Acetamido-2-aminobutanoic acid | 160.0975 | 0.81 | 0.46882 | 0.703 | 0.61 | pos |
| L-Carnitine | 162.1123 | 0.77 | 0.11935 | 0.348 | 1.14 | pos |
| Dulcin | 163.0867 | 1.12 | 0.66837 | 0.832 | 0.26 | pos |
| Acetylcysteine | 164.0366 | 1.47 | 0.00124 | 0.043 | 1.89 | pos |
| Enol-phenylpyruvate | 165.0547 | 1.57 | 0.79628 | 0.906 | 0.21 | pos |
| L-Fucose | 165.1128 | 1.92 | 0.04111 | 0.210 | 1.50 | pos |
| 2,6-Diamino-4-hydroxy-5-N-methylformamidopyrimidine | 166.0724 | 2.26 | 0.03340 | 0.193 | 1.38 | pos |
| N,N-Dimethylhistidine | 166.0974 | 1 | 0.90854 | 0.960 | 0.19 | pos |
| L-Methionine S-oxide | 166.0533 | 0.98 | 0.97588 | 0.990 | 0.02 | pos |
| L-beta-Phenylalanine | 166.0864 | 1.14 | 0.56703 | 0.769 | 0.44 | pos |
| D-Phenyllactic acid | 167.0704 | 1.26 | 0.01156 | 0.122 | 1.75 | pos |
| Desaminotyrosine | 167.0128 | 1.28 | 0.01168 | 0.123 | 1.65 | pos |
| Phenylephrine | 168.1019 | 1.44 | 0.02659 | 0.175 | 1.47 | pos |
| Pyridoxamine | 169.097 | 0.58 | 0.25430 | 0.514 | 0.71 | pos |
| (R)-2-O-Sulfolactate | 169.9772 | 0.86 | 0.18015 | 0.429 | 0.92 | pos |
| 2-Keto-6-acetamidocaproate | 170.0813 | 2.16 | 0.00664 | 0.096 | 1.84 | pos |
| 1-Methylhistidine | 170.0923 | 1.93 | 0.00000 | 0.002 | 2.20 | pos |
| Gabapentin | 172.1333 | 1.42 | 0.19140 | 0.443 | 1.04 | pos |
| 3-Dehydroshikimate | 171.9931 | 1.04 | 0.63441 | 0.812 | 0.45 | pos |
| (2S,5S)-trans-Carboxymethylproline | 174.0874 | 2.67 | 0.01365 | 0.132 | 1.52 | pos |
| N-Acetylornithine | 175.1078 | 0.47 | 0.00137 | 0.045 | 1.96 | pos |
| Citrulline | 176.103 | 1.3 | 0.06533 | 0.262 | 1.22 | pos |
| Neocnidilide | 177.1274 | 0.24 | 0.04144 | 0.211 | 1.38 | pos |
| Cysteinylglycine | 179.0485 | 0.95 | 0.99854 | 0.999 | 0.02 | pos |
| L-Homophenylalanine | 180.1015 | 1 | 0.58601 | 0.782 | 0.35 | pos |
| myo-Inositol | 181.0141 | 0.89 | 0.53754 | 0.751 | 0.34 | pos |
| Ecgonine methyl ester | 182.1178 | 2.28 | 0.02901 | 0.182 | 1.40 | pos |
| Se-Methylselenocysteine | 182.9837 | 2.27 | 0.07900 | 0.286 | 1.15 | pos |
| Homovanillic acid | 182.9853 | 0.73 | 0.39964 | 0.651 | 0.56 | pos |
| 2-Hydroxy-3-(4-hydroxyphenyl)propanoic acid | 182.985 | 0.64 | 0.32174 | 0.582 | 0.64 | pos |
| Antiarol | 184.0734 | 0.49 | 0.83664 | 0.926 | 0.03 | pos |
| 3,5-Dihydroxy-phenylglycine | 184.0605 | 0.74 | 0.16809 | 0.414 | 0.85 | pos |
| Epinephrine | 184.0966 | 1.07 | 0.01132 | 0.122 | 1.61 | pos |
| Phosphorylcholine | 184.0733 | 1.16 | 0.16055 | 0.405 | 0.87 | pos |
| Sebacic acid | 185.1174 | 1.27 | 0.74883 | 0.880 | 0.20 | pos |
| Phosphohydroxypyruvic acid | 184.986 | 1.11 | 0.32244 | 0.582 | 0.76 | pos |
| N5-(L-1-Carboxyethyl)-L-ornithine | 187.1077 | 0.96 | 0.90795 | 0.960 | 0.01 | pos |
| Undecanoic acid | 186.9562 | 1.04 | 0.78173 | 0.899 | 0.35 | pos |
| Pantothenol | 188.1282 | 1.5 | 0.03195 | 0.189 | 1.37 | pos |
| (Z)-But-1-ene-1,2,4-tricarboxylate | 188.0377 | 0.69 | 0.49437 | 0.721 | 0.41 | pos |
| N6-Acetyl-L-lysine | 189.1232 | 0.93 | 0.46818 | 0.703 | 0.50 | pos |
| N6,N6,N6-Trimethyl-L-lysine | 189.1596 | 0.91 | 0.68281 | 0.842 | 0.25 | pos |
| N-Acetylglutamic acid | 190.0709 | 0.86 | 0.94264 | 0.975 | 0.21 | pos |
| Kynurenic acid | 190.0502 | 1.28 | 0.00024 | 0.019 | 2.09 | pos |
| Diaminopimelic acid | 191.1026 | 1.22 | 0.61943 | 0.802 | 0.35 | pos |
| Isocitric acid | 192.0326 | 2.55 | 0.00097 | 0.038 | 1.93 | pos |
| 5,6-Dihydroxy-3-methyl-2-oxo-1,2,5,6-tetrahydroquinoline | 193.0683 | 1.29 | 0.22237 | 0.479 | 1.04 | pos |
| Orciprenaline | 194.1174 | 1.24 | 0.04595 | 0.222 | 1.44 | pos |
| Caffeine | 195.0878 | 1.74 | 0.17495 | 0.423 | 0.99 | pos |
| Tyrosine methylester | 196.0967 | 1.83 | 0.16001 | 0.404 | 0.93 | pos |
| N-Acetylhistidine | 198.0876 | 1.08 | 0.51376 | 0.735 | 0.45 | pos |
| Dodecanoic acid | 199.9881 | 2.08 | 0.08542 | 0.298 | 1.29 | pos |
| Spermine | 203.2229 | 0.87 | 0.68159 | 0.841 | 0.20 | pos |
| L-Tryptophan | 205.0971 | 1.07 | 0.95758 | 0.982 | 0.15 | pos |
| Indolelactic acid | 206.0814 | 0.92 | 0.98007 | 0.991 | 0.19 | pos |
| Homocitric acid | 207.0506 | 0.99 | 0.94002 | 0.974 | 0.01 | pos |
| N-Acetyl-L-phenylalanine | 208.097 | 1.51 | 0.63130 | 0.810 | 0.41 | pos |
| (+)-7-Isojasmonic acid | 211.133 | 2.27 | 0.12399 | 0.354 | 1.00 | pos |
| Dodecanedioic acid | 213.1486 | 0.55 | 0.04561 | 0.221 | 1.32 | pos |
| Dethiobiotin | 213.9829 | 1.13 | 0.02517 | 0.171 | 1.48 | pos |
| beta-Alanyl-L-lysine | 217.1548 | 0.94 | 0.53538 | 0.749 | 0.33 | pos |
| Propionylcarnitine | 218.1387 | 1.26 | 0.55926 | 0.764 | 0.40 | pos |
| Gamma-glutamyl-L-putrescine | 218.1501 | 0.83 | 0.64380 | 0.818 | 0.20 | pos |
| D-Lysopine | 219.1339 | 53.7 | 0.09863 | 0.318 | 1.11 | pos |
| N-Acetylserotonin | 219.1129 | 1.68 | 0.11173 | 0.338 | 1.03 | pos |
| Capsidiol | 219.1742 | 1 | 0.63473 | 0.812 | 0.31 | pos |
| N-Acetyl-D-glucosaminate | 220.0816 | 1.55 | 0.12481 | 0.355 | 1.10 | pos |
| (-)-threo-Iso(homo)2-citrate | 220.0642 | 1.4 | 0.85361 | 0.935 | 0.25 | pos |
| 5-Hydroxy-L-tryptophan | 221.0921 | 0.91 | 0.73023 | 0.870 | 0.13 | pos |
| N-Acetyl-D-galactosamine | 222.0972 | 1.06 | 0.93131 | 0.970 | 0.14 | pos |
| Metaxalone | 222.1128 | 0.68 | 0.10702 | 0.331 | 1.21 | pos |
| Allocystathionine | 222.0796 | 0.96 | 0.89898 | 0.956 | 0.19 | pos |
| Sinapic acid | 224.0739 | 1.44 | 0.04923 | 0.228 | 1.26 | pos |
| Methyl jasmonate | 224.0119 | 1.53 | 0.00169 | 0.049 | 1.79 | pos |
| Hydroxykynurenine | 225.0871 | 1.11 | 0.42077 | 0.669 | 0.65 | pos |
| Cytidine | 226.0823 | 0.79 | 0.95901 | 0.982 | 0.08 | pos |
| Porphobilinogen | 226.1803 | 1.25 | 0.19797 | 0.451 | 0.82 | pos |
| Biotin | 227.0848 | 1.06 | 0.15394 | 0.396 | 0.97 | pos |
| Myristoleic acid | 227.2004 | 1.52 | 0.07900 | 0.286 | 1.24 | pos |
| Benz[a]anthracene | 229.1005 | 1.4 | 0.05436 | 0.239 | 1.29 | pos |
| Ergothioneine | 231.0979 | 3.08 | 0.03408 | 0.195 | 1.40 | pos |
| Butyryl-L-carnitine | 232.1542 | 2.63 | 0.00096 | 0.038 | 1.90 | pos |
| N(omega)-Nitro-L-arginine methyl ester | 233.1134 | 0.94 | 0.69193 | 0.848 | 0.17 | pos |
| N-[(2S)-2-Amino-2-carboxyethyl]-L-glutamate | 234.0795 | 0.77 | 0.04455 | 0.219 | 1.25 | pos |
| Biopterin | 238.0898 | 2.16 | 0.03969 | 0.207 | 1.33 | pos |
| 4a-Carbinolamine tetrahydrobiopterin | 239.1027 | 1.05 | 0.49111 | 0.719 | 0.63 | pos |
| 1-Hexadecanol | 243.1833 | 1.1 | 0.31821 | 0.579 | 0.63 | pos |
| Lumichrome | 243.0874 | 1.73 | 0.33776 | 0.597 | 0.75 | pos |
| Juvenile hormone III | 249.1851 | 0.91 | 0.38737 | 0.641 | 0.55 | pos |
| OPEO | 251.2003 | 0.81 | 0.20385 | 0.457 | 0.88 | pos |
| Citrinin | 251.0918 | 0.39 | 0.07968 | 0.287 | 1.13 | pos |
| 16-Oxopalmitate | 253.2161 | 0.52 | 0.11136 | 0.338 | 1.02 | pos |
| Methyl hexadecanoic acid | 253.2524 | 0.51 | 0.08945 | 0.305 | 1.14 | pos |
| (9Z)-Hexadecenoic acid | 255.2321 | 0.64 | 0.25208 | 0.512 | 0.73 | pos |
| Glycerophosphocholine | 258.113 | 0.77 | 0.00856 | 0.108 | 1.70 | pos |
| Linatine | 260.1247 | 1.33 | 0.08056 | 0.289 | 1.29 | pos |
| Pantetheine | 261.1263 | 1.71 | 0.01803 | 0.148 | 1.43 | pos |
| Mannitol 1-phosphate | 262.038 | 1.37 | 0.08687 | 0.301 | 1.22 | pos |
| Linoleic acid | 263.2367 | 0.2 | 0.00096 | 0.038 | 1.98 | pos |
| Subaphylline | 265.1546 | 1.45 | 0.41559 | 0.664 | 0.71 | pos |
| Adenosine | 268.1043 | 0.7 | 0.59960 | 0.790 | 0.20 | pos |
| Inosine | 269.0879 | 1.99 | 0.05220 | 0.234 | 1.23 | pos |
| Retinol | 269.2265 | 1.34 | 0.28312 | 0.543 | 0.67 | pos |
| Medicarpin | 270.0914 | 0.76 | 0.54817 | 0.757 | 0.39 | pos |
| Genistein | 271.06 | 1.58 | 0.00322 | 0.067 | 1.87 | pos |
| Aurin | 273.0841 | 3.17 | 0.00601 | 0.091 | 1.69 | pos |
| Nandrolone | 275.2005 | 1.37 | 0.17650 | 0.425 | 1.05 | pos |
| Saccharopine | 276.1186 | 0.93 | 0.54737 | 0.757 | 0.52 | pos |
| 4-Hydroxycinnamoylagmatine | 276.1441 | 1.54 | 0.11972 | 0.348 | 1.18 | pos |
| (5-L-Glutamyl)-L-glutamate | 277.1013 | 2.15 | 0.01076 | 0.120 | 1.67 | pos |
| Alpha-dimorphecolic acid | 279.2319 | 0.37 | 0.04441 | 0.219 | 1.22 | pos |
| 13S-hydroxyoctadecadienoic acid | 279.2317 | 0.27 | 0.01012 | 0.117 | 1.55 | pos |
| Dibutyl phthalate | 279.159 | 1.41 | 0.80042 | 0.908 | 0.19 | pos |
| 3-Ketosphingosine | 280.2639 | 0.98 | 0.74769 | 0.879 | 0.20 | pos |
| Cyclopeptine | 281.1136 | 0.88 | 0.77243 | 0.894 | 0.07 | pos |
| 1-(3,4-Dihydroxyphenyl)-5-hydroxy-3-decanone | 281.1494 | 1.78 | 0.14028 | 0.377 | 0.98 | pos |
| Stearolic acid | 281.2474 | 1.13 | 0.28749 | 0.548 | 0.70 | pos |
| Oleamide | 282.279 | 0.81 | 0.66732 | 0.832 | 0.20 | pos |
| Sphingosine | 282.2788 | 0.88 | 0.74126 | 0.876 | 0.24 | pos |
| Vaccenic acid | 282.2511 | 1.05 | 0.79231 | 0.904 | 0.23 | pos |
| Oleic acid | 283.2604 | 0.76 | 0.41419 | 0.663 | 0.60 | pos |
| Octadecanamide | 284.2946 | 1.51 | 0.07924 | 0.287 | 1.08 | pos |
| (10S)-Juvenile hormone III diol | 285.2058 | 0.77 | 0.23892 | 0.497 | 0.83 | pos |
| 9-cis-Retinal | 285.2211 | 1.13 | 0.32567 | 0.586 | 0.84 | pos |
| Neoabietic acid | 285.2211 | 1.2 | 0.93135 | 0.970 | 0.08 | pos |
| Retinal | 285.221 | 0.49 | 0.05613 | 0.243 | 1.33 | pos |
| Dihydrobiochanin A | 286.0862 | 1.46 | 0.26263 | 0.522 | 0.77 | pos |
| Aspartame | 295.1287 | 0.77 | 0.37680 | 0.630 | 0.49 | pos |
| 13(S)-HOT | 295.2267 | 0.88 | 0.64176 | 0.817 | 0.24 | pos |
| (2'E,4'Z,8E)-Colneleic acid | 295.2269 | 1.18 | 0.74828 | 0.880 | 0.25 | pos |
| Exemestane | 297.1851 | 2 | 0.21872 | 0.475 | 0.89 | pos |
| 9,10-DHOME | 297.2418 | 1.12 | 0.45803 | 0.696 | 0.53 | pos |
| 12,13-DHOME | 297.2419 | 1.26 | 0.97834 | 0.991 | 0.00 | pos |
| Tridemorph | 298.3105 | 1.09 | 0.56811 | 0.770 | 0.52 | pos |
| D-4'-Phosphopantothenate | 300.0839 | 1.09 | 0.72203 | 0.866 | 0.29 | pos |
| (R)-10-Hydroxystearate | 300.2609 | 0.33 | 0.00759 | 0.103 | 1.61 | pos |
| Palmitoylethanolamide | 300.2893 | 1.07 | 0.79472 | 0.905 | 0.18 | pos |
| Sphinganine | 301.2933 | 1.41 | 0.15009 | 0.390 | 1.05 | pos |
| Isotretinoin | 301.2165 | 0.83 | 0.32374 | 0.584 | 0.71 | pos |
| 11alpha,17beta-Dihydroxy-17-methylandrost-4-en-3-one | 301.2162 | 0.62 | 0.18677 | 0.438 | 0.79 | pos |
| 9-cis-Retinoic acid | 301.2165 | 0.58 | 0.01383 | 0.133 | 1.60 | pos |
| 19(S)-HETE | 303.2318 | 0.95 | 0.47314 | 0.706 | 0.45 | pos |
| 8-HETE | 303.2318 | 0.86 | 0.56785 | 0.770 | 0.40 | pos |
| 11(R)-HETE | 303.2318 | 0.98 | 0.81062 | 0.912 | 0.01 | pos |
| N(6)-[(Indol-3-yl)acetyl]-L-lysine | 304.1615 | 1.2 | 0.65310 | 0.824 | 0.37 | pos |
| Arachidonic acid | 305.2471 | 1.5 | 0.08370 | 0.295 | 1.07 | pos |
| Oleoylethanolamide | 308.295 | 1.01 | 0.77430 | 0.895 | 0.22 | pos |
| 2,3-Dinor-8-iso prostaglandin F2alpha | 309.2056 | 1.97 | 0.05837 | 0.247 | 1.22 | pos |
| N-Acetylneuraminic acid | 310.1125 | 0.83 | 0.86827 | 0.942 | 0.10 | pos |
| 9(S)-HPOT | 311.2213 | 1.5 | 0.79668 | 0.906 | 0.20 | pos |
| Methoprene | 311.2576 | 0.8 | 0.58008 | 0.778 | 0.31 | pos |
| 9,10-12,13-Diepoxyoctadecanoate | 313.2377 | 0.43 | 0.02796 | 0.179 | 1.40 | pos |
| 8(R)-Hydroperoxylinoleic acid | 313.237 | 0.41 | 0.02960 | 0.183 | 1.37 | pos |
| 15-Deoxy-d-12,14-PGJ2 | 317.2114 | 0.61 | 0.05161 | 0.233 | 1.33 | pos |
| 9,10-Dihydroxystearate | 317.269 | 1.45 | 0.19558 | 0.448 | 0.94 | pos |
| 5a-Pregnane-3,20-dione | 317.2476 | 0.76 | 0.20875 | 0.463 | 0.85 | pos |
| Menthyl pyrrolidone carboxylate | 319.211 | 9.2 | 0.17877 | 0.428 | 0.89 | pos |
| 12-KETE | 319.2272 | 1.67 | 0.06000 | 0.251 | 1.26 | pos |
| 5-KETE | 319.2269 | 2.11 | 0.49170 | 0.719 | 0.51 | pos |
| 5(S)-HpETE | 319.2266 | 0.2 | 0.00000 | 0.001 | 2.20 | pos |
| 12-Keto-tetrahydro-leukotriene B4 | 319.2264 | 1.23 | 0.25951 | 0.519 | 0.64 | pos |
| Prostaglandin B1 | 319.2272 | 1.14 | 0.98210 | 0.992 | 0.03 | pos |
| Bitertanol | 320.1677 | 0.85 | 0.65930 | 0.827 | 0.45 | pos |
| 8,9-DiHETrE | 321.242 | 0.82 | 0.80113 | 0.908 | 0.13 | pos |
| gamma-L-Glutamyl-L-cysteinyl-beta-alanine | 322.0765 | 2.07 | 0.17020 | 0.417 | 0.95 | pos |
| Zeranol | 323.1833 | 0.78 | 0.96499 | 0.985 | 0.16 | pos |
| Cellobiose | 325.1121 | 1.1 | 0.97532 | 0.990 | 0.04 | pos |
| UMP | 325.0426 | 1.63 | 0.06096 | 0.253 | 1.30 | pos |
| (S)-Reticuline | 329.1495 | 1.84 | 0.00029 | 0.020 | 1.95 | pos |
| 2,3-Dinor-8-iso prostaglandin F1alpha | 329.2321 | 3.24 | 0.02177 | 0.161 | 1.43 | pos |
| Docosahexaenoic acid | 329.2476 | 0.95 | 0.83906 | 0.928 | 0.28 | pos |
| Cannabielsoin | 330.227 | 0.97 | 0.65949 | 0.827 | 0.47 | pos |
| 17alpha,21-Dihydroxypregnenolone | 331.2267 | 1.13 | 0.97727 | 0.991 | 0.06 | pos |
| Ethyl icosapentate | 331.2626 | 2.15 | 0.00324 | 0.067 | 1.84 | pos |
| Spectinomycin | 332.1522 | 1.76 | 0.14429 | 0.383 | 0.96 | pos |
| 21-Hydroxypregnenolone | 333.242 | 0.23 | 0.02471 | 0.170 | 1.40 | pos |
| 9S-hydroxy-11,15-dioxo-5Z,13E-prostadienoic acid | 333.2061 | 1.96 | 0.00773 | 0.103 | 1.54 | pos |
| Andrographolide | 333.2061 | 0.7 | 0.82487 | 0.920 | 0.10 | pos |
| Prostaglandin-c2 | 334.2045 | 3.36 | 0.00236 | 0.058 | 1.83 | pos |
| Delta-12-Prostaglandin J2 | 334.2091 | 1.81 | 0.08083 | 0.289 | 1.21 | pos |
| Nicotinamide ribotide | 334.2945 | 11.16 | 0.02113 | 0.159 | 1.37 | pos |
| Prostaglandin I2 | 335.2212 | 1.51 | 0.40966 | 0.658 | 0.61 | pos |
| Prostaglandin H2 | 335.2211 | 3.31 | 0.37365 | 0.627 | 0.61 | pos |
| Senecionine | 336.1918 | 2.04 | 0.00244 | 0.059 | 1.92 | pos |
| Isopentenyl adenosine | 336.1656 | 1.15 | 0.55508 | 0.762 | 0.51 | pos |
| Bufadienolide | 337.2485 | 0.68 | 0.15604 | 0.399 | 0.93 | pos |
| Prostaglandin E1 | 337.2376 | 0.57 | 0.63570 | 0.813 | 0.32 | pos |
| Prostaglandin F2b | 337.2377 | 1.56 | 0.16252 | 0.407 | 1.02 | pos |
| Kyotorphin | 337.1705 | 22.76 | 0.00155 | 0.047 | 1.83 | pos |
| Erucic acid | 338.3418 | 0.5 | 0.17151 | 0.418 | 1.09 | pos |
| (4Z,7Z,10Z,13Z,16Z,19Z)-Docosahexaenoic acid ethyl ester | 339.2684 | 0.42 | 0.00516 | 0.085 | 1.85 | pos |
| 11,12-DiHETrE | 339.2526 | 0.67 | 0.18470 | 0.435 | 0.83 | pos |
| 5,6-DHET | 339.2525 | 0.8 | 0.16624 | 0.411 | 1.01 | pos |
| D-Maltose | 342.14 | 0.81 | 0.66989 | 0.834 | 0.18 | pos |
| Corticosterone | 346.3307 | 0.19 | 0.09557 | 0.313 | 1.07 | pos |
| Quinestrol | 347.2424 | 0.29 | 0.00956 | 0.114 | 1.58 | pos |
| 3'-AMP | 348.07 | 1.26 | 0.00238 | 0.058 | 1.81 | pos |
| AMP | 348.07 | 1.65 | 0.00142 | 0.046 | 1.91 | pos |
| S-(Formylmethyl)glutathione | 350.1016 | 0.63 | 0.03922 | 0.206 | 1.24 | pos |
| 6-Ketoprostaglandin E1 | 351.2161 | 0.57 | 0.69681 | 0.851 | 0.19 | pos |
| Lipoxin B4 | 352.2304 | 1.13 | 0.80122 | 0.908 | 0.21 | pos |
| Prostaglandin F3a | 353.2296 | 0.42 | 0.00827 | 0.107 | 1.63 | pos |
| Prostaglandin E2 | 353.2314 | 0.36 | 0.04381 | 0.217 | 1.31 | pos |
| Trioxilin A3 | 355.2479 | 1.22 | 0.87915 | 0.947 | 0.17 | pos |
| Prostaglandin F2a | 355.2478 | 1.17 | 0.06638 | 0.264 | 1.18 | pos |
| Nitrendipine | 361.1387 | 2.08 | 0.11256 | 0.339 | 1.15 | pos |
| 1-Arachidonoylglycerol | 361.2734 | 1.03 | 0.93842 | 0.974 | 0.08 | pos |
| Aldosterone | 361.2216 | 0.84 | 0.44677 | 0.688 | 0.55 | pos |
| Thyrotropin releasing hormone | 362.3258 | 2.13 | 0.00094 | 0.038 | 1.81 | pos |
| GMP | 364.0649 | 0.83 | 0.38218 | 0.637 | 0.64 | pos |
| N-Acetyllactosamine | 366.1327 | 1.01 | 0.83847 | 0.927 | 0.21 | pos |
| Misoprostol | 368.2595 | 1.53 | 0.02033 | 0.156 | 1.59 | pos |
| 11-Dehydro-thromboxane B2 | 369.2238 | 0.28 | 0.00665 | 0.096 | 1.70 | pos |
| Carboprost | 369.2632 | 0.76 | 0.52532 | 0.742 | 0.42 | pos |
| Cholesterol | 369.3514 | 0.95 | 0.74434 | 0.878 | 0.26 | pos |
| 6-Keto-prostaglandin F1a | 371.2425 | 0.65 | 0.13985 | 0.377 | 0.96 | pos |
| 3-Geranylgeranylindole | 372.2957 | 1.34 | 0.13517 | 0.370 | 1.02 | pos |
| Riboflavin | 377.1451 | 1.15 | 0.57453 | 0.774 | 0.47 | pos |
| Resolvin D2 | 377.2302 | 0.4 | 0.05117 | 0.232 | 1.21 | pos |
| Riboflavin reduced | 378.1663 | 1.13 | 0.54243 | 0.753 | 0.55 | pos |
| S-Lactoylglutathione | 380.1121 | 0.64 | 0.57892 | 0.777 | 0.28 | pos |
| 7-Dehydrodesmosterol | 383.3303 | 1.16 | 0.12162 | 0.351 | 1.15 | pos |
| S-Adenosylhomocysteine | 385.1264 | 1.16 | 0.38293 | 0.637 | 0.69 | pos |
| Zymosterol intermediate 2 | 385.3456 | 1.11 | 0.72101 | 0.865 | 0.43 | pos |
| 7-Dehydrocholesterol | 385.3466 | 1.64 | 0.02368 | 0.167 | 1.54 | pos |
| Cholestenone | 385.3455 | 2.38 | 0.00194 | 0.053 | 1.79 | pos |
| Vitamin D3 | 385.3468 | 0.54 | 0.47808 | 0.709 | 0.59 | pos |
| Bufalin | 386.2531 | 0.3 | 0.01781 | 0.147 | 1.66 | pos |
| Ursodeoxycholic acid | 393.2083 | 1.66 | 0.16164 | 0.407 | 1.04 | pos |
| Yamogenin | 397.31 | 0.97 | 0.74214 | 0.876 | 0.28 | pos |
| Palmitoyl-L-carnitine | 400.3408 | 1.39 | 0.01242 | 0.127 | 1.67 | pos |
| Myriocin | 402.2847 | 0.57 | 0.59089 | 0.785 | 0.35 | pos |
| Allocholic acid | 408.3681 | 1.22 | 0.03277 | 0.192 | 1.42 | pos |
| Coleonol | 411.2356 | 0.48 | 0.12941 | 0.362 | 1.04 | pos |
| Testosterone cypionate | 412.3054 | 1.56 | 0.11396 | 0.341 | 1.16 | pos |
| 4-Methylamino-4-de(dimethylamino)anhydrotetracycline | 413.1402 | 1.32 | 0.42719 | 0.673 | 0.55 | pos |
| Sodium deoxycholate | 414.3213 | 1.99 | 0.05129 | 0.232 | 1.45 | pos |
| Calcitriol | 417.3365 | 0.79 | 0.26997 | 0.530 | 0.77 | pos |
| Benazepril | 425.2135 | 1.08 | 0.71141 | 0.859 | 0.33 | pos |
| Alpha-Tocotrienol | 425.3048 | 1.35 | 0.05128 | 0.232 | 1.31 | pos |
| ADP | 428.0359 | 0.83 | 0.21674 | 0.472 | 0.90 | pos |
| alpha-Tocopherol | 430.2422 | 0.9 | 0.82787 | 0.921 | 0.15 | pos |
| Hecogenin | 430.3161 | 0.54 | 0.19162 | 0.443 | 0.84 | pos |
| Biochanin A-beta-D-glucoside | 447.1367 | 1.61 | 0.12477 | 0.355 | 1.05 | pos |
| Baicalin | 447.0916 | 1.19 | 0.74452 | 0.878 | 0.23 | pos |
| Glutathionylaminopropylcadaverine | 448.2554 | 1.55 | 0.08257 | 0.293 | 1.24 | pos |
| 5-Methyltetrahydrofolic acid | 460.1902 | 1.27 | 0.19676 | 0.450 | 0.82 | pos |
| 3-Dehydroecdysone | 462.2651 | 0.99 | 0.70993 | 0.859 | 0.40 | pos |
| Adenylsuccinic acid | 464.0805 | 1.07 | 0.81496 | 0.914 | 0.25 | pos |
| 3-Epiecdysone | 464.2816 | 1.96 | 0.85144 | 0.934 | 0.19 | pos |
| Deoxyuridine-5'-triphosphate | 468.3879 | 0.44 | 0.00301 | 0.066 | 1.80 | pos |
| Retinoyl b-glucuronide | 476.2767 | 5.6 | 0.00088 | 0.036 | 1.84 | pos |
| Cytochalasin B | 480.276 | 1.33 | 0.88473 | 0.949 | 0.10 | pos |
| 26-Hydroxyecdysone | 480.3087 | 0.63 | 0.06343 | 0.257 | 1.22 | pos |
| Taurochenodesoxycholic acid | 482.2938 | 1.73 | 0.23871 | 0.496 | 0.89 | pos |
| Antibiotic JI-20A | 482.3239 | 0.82 | 0.28333 | 0.544 | 0.66 | pos |
| Citicoline | 489.1134 | 0.93 | 0.80073 | 0.908 | 0.14 | pos |
| 1-palmitoylglycerophosphocholine | 496.3396 | 3.4 | 0.25243 | 0.512 | 0.83 | pos |
| Taurocholic acid | 498.288 | 2.28 | 0.05828 | 0.247 | 1.35 | pos |
| Deltaline | 508.3025 | 1.27 | 0.76729 | 0.890 | 0.22 | pos |
| Taurohyocholate | 516.2983 | 1.93 | 0.20790 | 0.462 | 0.83 | pos |
| beta-Carotene | 536.1635 | 1.76 | 0.08309 | 0.294 | 1.28 | pos |
| Protoporphyrin IX | 563.2631 | 1.77 | 0.06292 | 0.257 | 1.23 | pos |
| 5-Oxoavermectin ''1b'' aglycone | 568.3369 | 0.34 | 0.03544 | 0.198 | 1.34 | pos |
| Avermectin B1b aglycone | 570.3511 | 1.94 | 0.58013 | 0.778 | 0.46 | pos |
| Mesobilirubinogen | 593.3303 | 1.58 | 0.11255 | 0.339 | 1.15 | pos |
| Rutin | 610.1817 | 1.16 | 0.59463 | 0.787 | 0.27 | pos |
| NAD | 664.1195 | 0.89 | 0.61625 | 0.800 | 0.27 | pos |
| Stachyose | 667.2253 | 0.73 | 0.75725 | 0.885 | 0.20 | pos |
| Quercetin 3-(2G-xylosylrutinoside) | 742.2078 | 1.74 | 0.03609 | 0.199 | 1.41 | pos |
| Ethylmethylacetic acid | 101.024 | 0.71 | 0.58407 | 0.774 | 0.45 | neg |
| L-Erythrulose | 101.0241 | 1.36 | 0.31390 | 0.555 | 0.82 | neg |
| (R)-3-Hydroxybutyric acid | 103.04 | 0.64 | 0.27857 | 0.522 | 0.70 | neg |
| Pyrrole-2-carboxylic acid | 110.0246 | 0.57 | 0.01657 | 0.134 | 1.56 | neg |
| Dihydrouracil | 112.986 | 1.59 | 0.18558 | 0.421 | 0.83 | neg |
| Creatinine | 112.9861 | 0.89 | 0.88624 | 0.945 | 0.17 | neg |
| L-Proline | 114.0558 | 2.01 | 0.71745 | 0.859 | 0.21 | neg |
| trans-1,2-Cyclohexanediol | 114.934 | 1.65 | 0.21427 | 0.455 | 0.84 | neg |
| Fumaric acid | 115.0033 | 0.7 | 0.20247 | 0.442 | 0.86 | neg |
| L-Valine | 116.0703 | 1.12 | 0.81569 | 0.910 | 0.18 | neg |
| L-Aspartate-semialdehyde | 116.0352 | 1.08 | 0.71241 | 0.856 | 0.39 | neg |
| Guanidoacetic acid | 116.9282 | 0.88 | 0.33438 | 0.573 | 0.71 | neg |
| Betaine | 116.9281 | 0.91 | 0.51429 | 0.726 | 0.45 | neg |
| Succinic acid | 118.0225 | 0.65 | 0.23445 | 0.476 | 0.83 | neg |
| 3-Methylthiopropionic acid | 119.0347 | 0.73 | 0.41058 | 0.643 | 0.54 | neg |
| Erythritol | 121.0294 | 1.02 | 0.67663 | 0.835 | 0.25 | neg |
| Phenylethylamine | 121.029 | 0.93 | 0.42790 | 0.658 | 0.46 | neg |
| Taurine | 124.0073 | 0.54 | 0.38375 | 0.620 | 0.50 | neg |
| 1-Naphthylamine | 123.9018 | 0.93 | 0.70266 | 0.850 | 0.33 | neg |
| Caprylic acid | 124.9878 | 1.93 | 0.33790 | 0.577 | 0.68 | neg |
| Pyroglutamic acid | 128.0359 | 0.64 | 0.99671 | 0.998 | 0.08 | neg |
| Cyanuric acid | 129.0191 | 1.21 | 0.95494 | 0.980 | 0.21 | neg |
| 3-Methyl-2-oxovaleric acid | 129.0556 | 1.76 | 0.12445 | 0.344 | 0.95 | neg |
| Ketoleucine | 129.0553 | 1.81 | 0.07439 | 0.267 | 1.21 | neg |
| Citramalic acid | 129.0191 | 1.86 | 0.09494 | 0.302 | 1.04 | neg |
| Leucine | 130.0866 | 2.29 | 0.01908 | 0.143 | 1.39 | neg |
| L-Isoleucine | 130.0858 | 1.8 | 0.11704 | 0.333 | 0.97 | neg |
| Creatine | 130.059 | 1.23 | 0.20282 | 0.442 | 0.93 | neg |
| Aminocaproic acid | 130.088 | 2.24 | 0.01482 | 0.127 | 1.42 | neg |
| L-Leucine | 130.088 | 1.35 | 0.40655 | 0.640 | 0.53 | neg |
| Beta-Leucine | 130.0858 | 2.33 | 0.00016 | 0.013 | 2.03 | neg |
| D-Ornithine | 131.0822 | 1.52 | 0.00468 | 0.074 | 1.88 | neg |
| (-)-Isopiperitenone | 131.0823 | 0.99 | 0.91088 | 0.958 | 0.01 | neg |
| Glutaric acid | 131.0346 | 0.75 | 0.54718 | 0.750 | 0.42 | neg |
| L-Aspartic acid | 132.0299 | 1.05 | 0.90932 | 0.957 | 0.13 | neg |
| L-Malic acid | 133.0141 | 1.09 | 0.41181 | 0.644 | 0.51 | neg |
| Adenine | 134.0466 | 1.17 | 0.25055 | 0.495 | 0.83 | neg |
| Hypoxanthine | 135.031 | 0.73 | 0.21153 | 0.452 | 0.88 | neg |
| Phenyl acetate | 134.8953 | 1.3 | 0.88580 | 0.945 | 0.03 | neg |
| Threonic acid | 135.0298 | 1.12 | 0.85661 | 0.931 | 0.07 | neg |
| Phenylacetic acid | 135.9714 | 1.27 | 0.74925 | 0.877 | 0.19 | neg |
| Urocanic acid | 137.0348 | 1.25 | 0.61778 | 0.797 | 0.29 | neg |
| 3-Hydroxypicolinic acid | 138.0194 | 1 | 0.98600 | 0.993 | 0.06 | neg |
| Salicylic acid | 137.9865 | 1.33 | 0.20654 | 0.446 | 0.94 | neg |
| Spermidine | 143.9154 | 1.13 | 0.58327 | 0.774 | 0.38 | neg |
| L-Glutamic acid | 146.0454 | 1.12 | 0.94105 | 0.974 | 0.07 | neg |
| trans-Cinnamate | 147.0451 | 0.52 | 0.00918 | 0.103 | 1.61 | neg |
| L-Methionine | 148.0429 | 1.14 | 0.26807 | 0.512 | 0.80 | neg |
| D-Ribose | 149.0454 | 1.84 | 0.18287 | 0.419 | 0.84 | neg |
| Mandelic acid | 151.0407 | 1.56 | 0.01468 | 0.127 | 1.52 | neg |
| (R)-mandelic Acid | 151.0407 | 1.53 | 0.00007 | 0.008 | 2.03 | neg |
| 3-Hydroxyanthranilic acid | 152.0351 | 0.89 | 0.13179 | 0.353 | 0.92 | neg |
| L-Histidine | 154.0627 | 2.4 | 0.00392 | 0.068 | 1.68 | neg |
| Oxoadipic acid | 158.9783 | 0.73 | 0.18319 | 0.419 | 0.87 | neg |
| Aminoadipic acid | 160.0611 | 0.97 | 0.63047 | 0.805 | 0.30 | neg |
| D-Glucose | 161.045 | 2.66 | 0.00007 | 0.008 | 2.04 | neg |
| Nicotine | 160.9355 | 1.04 | 0.75004 | 0.877 | 0.31 | neg |
| Phenylpyruvic acid | 163.0397 | 0.91 | 0.41340 | 0.645 | 0.59 | neg |
| L-Phenylalanine | 164.1273 | 1.22 | 0.64633 | 0.815 | 0.36 | neg |
| Phthalic acid | 165.0408 | 0.5 | 0.19389 | 0.430 | 0.73 | neg |
| 6-Methylmercaptopurine | 166.0179 | 0.82 | 0.59712 | 0.784 | 0.43 | neg |
| Uric acid | 167.0208 | 1.1 | 0.42115 | 0.653 | 0.60 | neg |
| Phosphoenolpyruvic acid | 167.9782 | 2.8 | 0.16442 | 0.398 | 0.94 | neg |
| Pyridoxine | 169.0617 | 0.75 | 0.55306 | 0.754 | 0.39 | neg |
| Beta-Glycerophosphoric acid | 171.0061 | 1.11 | 0.64437 | 0.814 | 0.35 | neg |
| N-Acetylleucine | 172.0987 | 1.37 | 0.24129 | 0.485 | 0.73 | neg |
| L-Arginine | 173.1039 | 1.01 | 0.89220 | 0.948 | 0.10 | neg |
| Amino acid(Arg-) | 173.1039 | 0.94 | 0.97768 | 0.991 | 0.15 | neg |
| 4-Quinolinecarboxylic acid | 173.118 | 1.09 | 0.26027 | 0.504 | 0.91 | neg |
| N-Acetyl-L-aspartic acid | 174.0404 | 1.54 | 0.21560 | 0.457 | 0.71 | neg |
| Ascorbate | 175.0243 | 0.54 | 0.54758 | 0.750 | 0.42 | neg |
| Guanidinosuccinic acid | 174.9571 | 1.13 | 0.90597 | 0.955 | 0.07 | neg |
| Gluconolactone | 178.051 | 0.67 | 0.00714 | 0.091 | 1.64 | neg |
| 2-Hydroxy-3-(4-hydroxyphenyl)propenoic acid | 179.035 | 0.65 | 0.05088 | 0.223 | 1.23 | neg |
| L-Tyrosine | 180.0653 | 1.79 | 0.29228 | 0.534 | 0.63 | neg |
| Fructose-1P | 180.0644 | 1.71 | 0.25306 | 0.497 | 0.64 | neg |
| (R)-3-(4-Hydroxyphenyl)lactate | 181.0505 | 0.66 | 0.07802 | 0.274 | 1.14 | neg |
| Sorbitol | 181.006 | 0.75 | 0.86249 | 0.934 | 0.11 | neg |
| 4-Pyridoxic acid | 182.0453 | 1.25 | 0.28235 | 0.525 | 0.67 | neg |
| Ecgonine | 184.0977 | 1.9 | 0.55588 | 0.756 | 0.35 | neg |
| Glycylleucine | 187.1082 | 1.16 | 0.35863 | 0.597 | 0.81 | neg |
| 10-Hydroxydecanoic acid | 187.1337 | 0.48 | 0.01164 | 0.114 | 1.51 | neg |
| Azelaic acid | 187.0976 | 1.18 | 0.44742 | 0.673 | 0.66 | neg |
| (2S)-2-{[1-(R)-Carboxyethyl]amino}pentanoate | 188.0926 | 1.32 | 0.58844 | 0.777 | 0.30 | neg |
| Citric acid | 191.0197 | 0.66 | 0.13962 | 0.364 | 1.04 | neg |
| 5-Hydroxyindoleacetic acid | 191.1074 | 0.88 | 0.62326 | 0.800 | 0.28 | neg |
| Gluconic acid | 195.0508 | 0.59 | 0.07060 | 0.261 | 1.17 | neg |
| 3,7-Dimethyluric acid | 195.0509 | 0.96 | 0.90790 | 0.957 | 0.12 | neg |
| Asymmetric dimethylarginine | 201.1352 | 0.47 | 0.03384 | 0.186 | 1.38 | neg |
| D-Erythritol 4-phosphate | 201.0223 | 1.31 | 0.16236 | 0.396 | 0.97 | neg |
| Tryptophanamide | 203.1119 | 1.08 | 0.88731 | 0.945 | 0.03 | neg |
| L-Kynurenine | 207.0771 | 1.64 | 0.05532 | 0.232 | 1.16 | neg |
| L-Arogenate | 208.0612 | 2.09 | 0.00007 | 0.008 | 2.06 | neg |
| D-Glycero-D-galacto-heptitol | 211.082 | 1.28 | 0.41790 | 0.649 | 0.64 | neg |
| Ribose 1-phosphate | 211.001 | 1.11 | 0.56092 | 0.760 | 0.37 | neg |
| Deoxyribose 5-phosphate | 213.0152 | 17.39 | 0.07554 | 0.269 | 1.18 | neg |
| 12-Hydroxydodecanoic acid | 215.1651 | 0.82 | 0.96539 | 0.984 | 0.11 | neg |
| Pantothenic acid | 219.106 | 0.74 | 0.03308 | 0.184 | 1.29 | neg |
| N-Acetylmannosamine | 221.0927 | 0.53 | 0.03340 | 0.185 | 1.38 | neg |
| N-Acetyl-D-glucosamine | 221.1543 | 1.09 | 0.00856 | 0.099 | 1.49 | neg |
| 6-Acetyl-D-glucose | 222.0769 | 1.1 | 0.74929 | 0.877 | 0.12 | neg |
| Thymidine | 223.0277 | 1.58 | 0.00288 | 0.057 | 1.77 | neg |
| Carnosine | 225.0991 | 1.44 | 0.59974 | 0.786 | 0.24 | neg |
| Deoxyuridine | 227.0678 | 1.1 | 0.60965 | 0.791 | 0.32 | neg |
| Myristic acid | 227.2016 | 0.48 | 0.06813 | 0.258 | 1.26 | neg |
| Pentadecanoic acid | 241.2171 | 0.78 | 0.72496 | 0.863 | 0.45 | neg |
| Uridine | 243.0619 | 1.41 | 0.10031 | 0.310 | 1.12 | neg |
| Gemfibrozil | 249.149 | 1.8 | 0.33647 | 0.575 | 0.60 | neg |
| gamma-Glutamylcysteine | 248.9598 | 6.84 | 0.00410 | 0.069 | 1.83 | neg |
| Deoxyinosine | 252.091 | 0.85 | 0.85839 | 0.932 | 0.02 | neg |
| 5-L-Glutamyl-taurine | 253.0493 | 1.03 | 0.75174 | 0.878 | 0.40 | neg |
| Daidzein | 254.054 | 0.89 | 0.13594 | 0.359 | 0.93 | neg |
| Palmitic acid | 255.2302 | 0.81 | 0.62671 | 0.802 | 0.27 | neg |
| Pentostatin | 267.1121 | 2.47 | 0.00173 | 0.043 | 1.89 | neg |
| Formononetin | 267.1958 | 1.18 | 0.31306 | 0.555 | 0.66 | neg |
| Apigenin | 269.0452 | 1.04 | 0.86515 | 0.935 | 0.08 | neg |
| Dehydroepiandrosterone | 269.2117 | 1.05 | 0.98924 | 0.995 | 0.05 | neg |
| Naringenin | 271.0608 | 1.91 | 0.00447 | 0.073 | 1.74 | neg |
| 16-Hydroxy hexadecanoic acid | 271.2281 | 1.72 | 0.00002 | 0.004 | 2.10 | neg |
| Epiandrosterone | 271.2281 | 5.9 | 0.00315 | 0.060 | 1.78 | neg |
| [8]-Shogaol | 275.1649 | 0.71 | 0.16166 | 0.395 | 0.91 | neg |
| Stearidonic acid | 275.2013 | 0.76 | 0.63094 | 0.805 | 0.42 | neg |
| 6-Phosphogluconic acid | 276.0202 | 1.07 | 0.42569 | 0.656 | 0.42 | neg |
| Gamma-Linolenic acid | 277.2169 | 0.87 | 0.12252 | 0.341 | 0.99 | neg |
| Alpha-Linolenic acid | 277.2166 | 0.95 | 0.65661 | 0.821 | 0.29 | neg |
| Bovinic acid | 279.2326 | 1.46 | 0.02375 | 0.157 | 1.47 | neg |
| (6Z)-Octadecenoic acid | 282.2517 | 0.98 | 0.97667 | 0.990 | 0.00 | neg |
| Xanthosine | 283.0679 | 1.02 | 0.35766 | 0.596 | 0.68 | neg |
| Glycitein | 283.0606 | 0.98 | 0.76610 | 0.886 | 0.32 | neg |
| Guanosine | 283.2639 | 2.06 | 0.00014 | 0.012 | 1.92 | neg |
| Stearic acid | 283.2611 | 1.46 | 0.17185 | 0.406 | 0.89 | neg |
| Hexadecanedioate | 285.2064 | 0.8 | 0.29509 | 0.537 | 0.81 | neg |
| 9-OxoODE | 293.2118 | 0.92 | 0.69125 | 0.844 | 0.32 | neg |
| 13-L-Hydroperoxylinoleic acid | 293.2119 | 1.15 | 0.74120 | 0.872 | 0.08 | neg |
| 5'-Methylthioadenosine | 296.0821 | 1.68 | 0.18519 | 0.421 | 0.95 | neg |
| 9,10-Epoxyoctadecenoic acid | 296.2307 | 1.6 | 0.32383 | 0.563 | 0.73 | neg |
| 5'-S-Methyl-5'-thioinosine | 297.0653 | 1.02 | 0.51967 | 0.730 | 0.43 | neg |
| Nonadecanoic acid | 297.2424 | 0.66 | 0.91746 | 0.961 | 0.06 | neg |
| all-trans-Retinoic acid | 299.2584 | 0.52 | 0.09981 | 0.309 | 1.07 | neg |
| EPA (d5) | 301.2169 | 1.25 | 0.28717 | 0.530 | 0.75 | neg |
| Glutathione | 306.076 | 1.35 | 0.68437 | 0.840 | 0.36 | neg |
| (-)-Epigallocatechin | 306.0759 | 1.1 | 0.81849 | 0.912 | 0.18 | neg |
| 8,11,14-Eicosatrienoic acid | 306.2511 | 0.88 | 0.86739 | 0.935 | 0.14 | neg |
| Ribose 1,5-bisphosphate | 309.1737 | 3.51 | 0.42207 | 0.653 | 0.48 | neg |
| 9,10,13-TriHOME | 311.2226 | 0.95 | 0.50049 | 0.716 | 0.40 | neg |
| 9(S)-HPODE | 311.2226 | 0.54 | 0.02645 | 0.165 | 1.45 | neg |
| Arachidic acid | 311.1679 | 0.33 | 0.01496 | 0.127 | 1.68 | neg |
| 16(R)-HETE | 319.2272 | 1.32 | 0.36682 | 0.604 | 0.59 | neg |
| 11-Dehydrocorticosterone | 325.184 | 0.36 | 0.03914 | 0.197 | 1.28 | neg |
| Docosapentaenoic acid (22n-3) | 329.2477 | 1.09 | 0.32862 | 0.568 | 0.66 | neg |
| Prostaglandin E3 | 331.1909 | 0.22 | 0.51666 | 0.728 | 0.58 | neg |
| Adrenic acid | 331.2636 | 3.55 | 0.00151 | 0.040 | 1.86 | neg |
| 12-Keto-leukotriene B4 | 333.2061 | 1.29 | 0.74110 | 0.872 | 0.19 | neg |
| 8-Isoprostane | 335.2222 | 0.75 | 0.03774 | 0.194 | 1.29 | neg |
| Acebutolol | 335.1888 | 3.15 | 0.26603 | 0.510 | 0.77 | neg |
| 11b-PGF2a | 335.2223 | 0.81 | 0.62391 | 0.800 | 0.17 | neg |
| (5Z,9E,14Z)-(8xi,11R,12S)-11,12-epoxy-8-hydroxyicosa-5,9,14-trienoic Acid | 335.2251 | 1.21 | 0.27016 | 0.514 | 0.74 | neg |
| Prostaglandin A1 | 335.2222 | 0.91 | 0.44081 | 0.668 | 0.49 | neg |
| Fructose 1,6-bisphosphate | 339.2002 | 0.72 | 0.06052 | 0.243 | 1.13 | neg |
| 2-Hydroxy-6-pentadecylbenzoic acid | 347.2581 | 0.58 | 0.06306 | 0.248 | 1.25 | neg |
| Pirenzepine | 350.1633 | 4.47 | 0.00001 | 0.002 | 2.14 | neg |
| (13E)-11a-Hydroxy-9,15-dioxoprost-13-enoic acid | 351.2173 | 1.13 | 0.20913 | 0.449 | 0.71 | neg |
| Prostaglandin F1a | 355.2484 | 0.5 | 0.03253 | 0.182 | 1.36 | neg |
| Rosmarinic acid | 359.0774 | 1.09 | 0.43073 | 0.660 | 0.63 | neg |
| Tetracosanoic acid | 367.2119 | 0.39 | 0.03170 | 0.180 | 1.42 | neg |
| Thromboxane B2 | 369.2278 | 0.77 | 0.74622 | 0.876 | 0.13 | neg |
| Sphingosine 1-phosphate | 378.232 | 0.59 | 0.34805 | 0.587 | 0.78 | neg |
| Chenodeoxycholic acid | 391.2843 | 1.13 | 0.34466 | 0.584 | 0.53 | neg |
| Deoxycholic acid | 392.2104 | 1.17 | 0.32515 | 0.564 | 0.68 | neg |
| 25-Hydroxycholesterol | 401.0866 | 0.73 | 0.06469 | 0.251 | 1.10 | neg |
| 1-palmitoyl-dihydroxyacetone-phosphate | 407.219 | 0.52 | 0.03602 | 0.190 | 1.35 | neg |
| LysoPA(16_0_0_0) | 409.2345 | 0.55 | 0.13078 | 0.352 | 0.99 | neg |
| Lupulone | 414.2676 | 0.87 | 0.47970 | 0.699 | 0.50 | neg |
| Paxilline | 434.2362 | 1.21 | 0.49512 | 0.712 | 0.52 | neg |
| Glycochenodeoxycholic acid | 448.3044 | 1.32 | 0.80825 | 0.907 | 0.19 | neg |
| FMN | 455.0949 | 0.79 | 0.62299 | 0.800 | 0.32 | neg |
| Cholesterol sulfate | 465.3024 | 1.26 | 0.80115 | 0.903 | 0.21 | neg |
| Leukotriene D4 | 477.2485 | 0.46 | 0.03388 | 0.186 | 1.39 | neg |
| Mupirocin | 499.2778 | 0.71 | 0.13927 | 0.364 | 1.01 | neg |
| ATP | 507.1189 | 1.07 | 0.80871 | 0.907 | 0.12 | neg |
| Probucol | 515.3034 | 2.49 | 0.10219 | 0.312 | 1.20 | neg |
| Adenosine diphosphate ribose | 540.0468 | 1.33 | 0.26685 | 0.511 | 0.77 | neg |
| Uridine diphosphate glucose | 565.0464 | 0.98 | 0.93671 | 0.972 | 0.06 | neg |
| Leukotriene C4 | 624.2931 | 0.56 | 0.11073 | 0.325 | 1.07 | neg |
| Maltotetraose | 665.2081 | 0.82 | 0.20890 | 0.449 | 0.76 | neg |
| Cellopentaose | 827.2606 | 0.65 | 0.15668 | 0.387 | 1.05 | neg |
| Maltohexaose | 989.3159 | 0.64 | 0.67604 | 0.835 | 0.28 | neg |
