# Supplementary material for: Comprehensive analysis of histophysiology, transcriptomics and metabolomics in goslings exposed to gossypol acetate: unraveling hepatotoxic mechanisms
Source: Front Vet Sci. 2025 Jan 21;12:1527284. doi: 10.3389/fvets.2025.1527284 (PMC11792171; doi:10.3389/fvets.2025.1527284)
Supplement: Supplementary file 1 [file Data_Sheet_1.zip › supplementary materials/Table S3. Data production of RNA-seq.docx]

**Table S3.** Data production of RNA-seq.

| Samples^1^ | Raw reads | Raw bases^2^ | Clean reads | Clean bases | Error rate (%) | Q30 (%) | GC content (%) | Mapped reads | Unique map | Multi map |
| --- | --- | --- | --- | --- | --- | --- | --- | --- | --- | --- |
| GA1_14 | 45662388 | 6.85G | 43649048 | 6.55G | 0.03 | 87.33 | 47.87 | 32953737  (75.50%) | 31940773  (73.18%) | 1012964  (2.32%) |
| GA2_14 | 42747600 | 6.41G | 41641978 | 6.25G | 0.03 | 88.26 | 48.22 | 33753343  (81.06%) | 32749960 (78.65%) | 1003383  (2.41%) |
| GA3_14 | 46163350 | 6.92G | 44854284 | 6.73G | 0.03 | 88.33 | 47.38 | 35533701  (79.22%) | 34445687  (76.79%) | 1088014  (2.43%) |
| GA4_14 | 46098904 | 6.91G | 44668162 | 6.7G | 0.03 | 87.53 | 47.27 | 34250830  (76.68%) | 33278174  (74.50%) | 972656  (2.18%) |
| GC1_14 | 39571998 | 5.94G | 38037932 | 5.71G | 0.03 | 87.92 | 47.58 | 28222812  (74.20%) | 27402357  (72.04%) | 820455  (2.16%) |
| GC2_14 | 40008544 | 6.00G | 38866762 | 5.83G | 0.03 | 87.86 | 48.69 | 29430931  (75.72%) | 28381681 (73.02%) | 1049250  (2.70%) |
| GC3_14 | 43622648 | 6.54G | 42413228 | 6.36G | 0.03 | 87.75 | 47.52 | 33703959  (79.47%) | 32486817  (76.60%) | 1217142  (2.87%) |
| GC4_14 | 39542614 | 5.93G | 38197474 | 5.73G | 0.03 | 87.57 | 47.4 | 30312777  (79.36%) | 29360335  (76.86%) | 952442  (2.49%) |

^1^GA1_14, GA2_14, GA3_14 and GA4_14 were the control group samples, and GC1_14, GC2_14, GC3_14, and GC4_14 were the GA50 group samples.

^2^1G of data is equal to 1 billion bases.
