# Supplementary material for: Comprehensive analysis of histophysiology, transcriptomics and metabolomics in goslings exposed to gossypol acetate: unraveling hepatotoxic mechanisms
Source: Front Vet Sci. 2025 Jan 21;12:1527284. doi: 10.3389/fvets.2025.1527284 (PMC11792171; doi:10.3389/fvets.2025.1527284)
Supplement: Supplementary file 1 [file Data_Sheet_1.zip › supplementary materials/Table S1. Composition and nutrient levels of the experimental diets (as-fed basis).docx]

**Table S1.** Composition and nutrient levels of the experimental diets (as-fed basis).

| Items | Contents |
| --- | --- |
| Ingredient, % |  |
| Corn | 60.50 |
| Soybean meal | 29.00 |
| Wheat bran | 6.80 |
| Limestone | 1.00 |
| Calcium hydrogen phosphate | 1.30 |
| DL-Methionine^1^ | 0.10 |
| Salt | 0.30 |
| Premix^2^ | 1.00 |
| Total | 100.00 |
| Nutrient level^3^, % |  |
| Metabolizable Energy (MJ/kg) | 11.49 |
| Crude protein | 18.75 |
| Crude fiber | 3.54 |
| Calcium | 0.80 |
| Total phosphorus | 0.63 |
| Available phosphorus | 0.40 |
| Lysine | 0.97 |
| Methionine | 0.40 |

^1^DL-methionine is a racemic mixture that consists of 50% D-methionine and 50% L-methionine.

^2^Provided per kilogram of complete diet：vitamin A 9 000 IU, vitamin D 3 000 IU, vitamin E 18 IU, vitamin K 1.5 mg, vitamin B_1_ 0.9 mg, vitamin B_2_ 8 mg, vitamin B_6_ 3.2 mg, vitamin B_12_ 0.01 mg, nicotinic acid 45 mg, pantothenic acid 11 mg, folic acid 0.65 mg, biotin 0.05 mg, choline 0.35 g, Fe (as ferrous sulphate) 60 mg, Cu (as copper sulphate) 10 mg, Mn (as manganese sulphate) 95 mg, Zn (as zinc sulphate) 90 g, I (as potassium iodide) 0.5 mg, Se (as sodium selenite) 0.3 mg.

^3^Nutrient levels were calculated values.
